# Worldwide Burden, Risk Factors, and Temporal Trends of Ovarian Cancer: A Global Study

**DOI:** 10.3390/cancers14092230

**Published:** 2022-04-29

**Authors:** Junjie Huang, Wing Chung Chan, Chun Ho Ngai, Veeleah Lok, Lin Zhang, Don Eliseo Lucero-Prisno, Wanghong Xu, Zhi-Jie Zheng, Edmar Elcarte, Mellissa Withers, Martin C. S. Wong, on behalf of NCD Global Health Research Group of Association of Pacific Rim Universities (APRU)

**Affiliations:** 1 The Jockey Club School of Public Health and Primary Care, Faculty of Medicine, Chinese University of Hong Kong, Hong Kong 999077, China; junjie_huang@link.cuhk.edu.hk (J.H.); wingchungchan@cuhk.edu.hk (W.C.C.); alfonse.ngai@gmail.com (C.H.N.); 2 Department of Global Public Health, Karolinska Institute, Karolinska University Hospital, 171 77 Stockholm, Sweden; veeleahlok@gmail.com; 3 School of Population and Global Health, The University of Melbourne, Melbourne, VIC 3000, Australia; tony1982110@gmail.com; 4 School of Public Health, The Chinese Academy of Medical Sciences and Peking Union Medical College, Beijing 100005, China; 5 Department of Global Health and Development, London School of Hygiene and Tropical Medicine, London WC1E 7HT, UK; don-eliseo.lucero-prisno@lshtm.ac.uk; 6 School of Public Health, Fudan University, Shanghai 200433, China; wanghong.xu@fudan.edu.cn; 7 Department of Global Health, School of Public Health, Peking University, Beijing 100191, China; zhengzj@bjmu.edu.cn; 8 College of Nursing, University of the Philippines, Pedro Gil, Manila 1101, Philippines; eselcarte@up.edu.ph; 9 Department of Preventive Medicine, Institute for Global Health, University of Southern California, Los Angeles, CA 90007, USA

**Keywords:** incidence, mortality, risk factors, temporal trends, ovarian cancer

## Abstract

**Simple Summary:**

Ovarian cancer was the third most common gynecological cancer globally in 2020. Ovarian carcinoma is the most common type of ovarian cancer, comprising over 90% of all ovarian cancer cases. The risk of ovarian cancer increases in females with age, along with having a family history, having a family cancer syndrome, and breast cancer susceptibility gene (BRCA) mutations. Investigation of the latest disease burden, risk factors, and temporal trends of ovarian cancer is important for the reduction of its associated mortality globally. The global incidence and mortality rates of ovarian cancer for 185 countries in 2020 were retrieved from the Global Cancer Observatory (GLOBOCAN) database established by the International Agency for Research on Cancer (IARC, WHO, Lyon, France). The incidence of ovarian cancer has been increasing substantially among younger females, probably caused by the increasing prevalence of obesity, metabolic syndrome, estrogen exposure and nulliparity.

**Abstract:**

This study aimed to investigate the most updated worldwide incidence and mortality, risk factors, and epidemiologic trend of ovarian cancer in different countries, regions, and age groups. The *Global Cancer Observatory* database was used for incidence and mortality rates of ovarian cancer in 2020. Data from *Cancer Incidence in Five Continents* and the *WHO mortality database* was accessed for trend analysis. Age-standardized rates (ASRs, per 100,000 persons) were calculated for incidence and mortality. The 10-year annual average percent change (AAPC) was estimated by Joinpoint regression analysis. There was an overall decreasing trend of ovarian cancer, yet its burden has been increasing in lower-income countries and among younger females in some countries. Intensive lifestyle modifications are warranted, especially for the populations at high risk for ovarian cancer, including smoking cessation, alcohol use reduction, physical activity, weight control, and treatment of metabolic diseases.

## 1. Introduction

Ovarian cancer was the third most common gynecological cancer globally in 2020 [1]. Ovarian carcinoma is the most common type of ovarian cancer, comprising over 90% of all ovarian cancer cases. There are five main histological types of ovarian carcinoma, of which high-grade serous carcinoma is the most common [2]. Ovarian cancer is often diagnosed at a late stage, making this malignancy the most lethal gynecological cancer. The prognosis of ovarian cancer is usually poor, with a 5-year survival rate of only 17% for a patient at an advanced stage [3]. Therefore, it is imperative to understand the etiology of ovarian cancer and identify its risk factors and populations at high risk in order to prevent this disease.

The risk of ovarian cancer increases in females with age, along with having a family history, having a family cancer syndrome, and breast cancer susceptibility gene (BRCA) mutations [4]. The reproductive-related risk factors for ovarian cancer include having children later or never having a full-term pregnancy, reaching menopause at an older age, and taking hormone therapy after menopause [5]. Other common modifiable lifestyle and metabolic risk factors include smoking, alcohol use, physical inactivity, unhealthy diet, obesity, and diabetes [6]. A comprehensive understanding of the disease burden attributable to the preventable risk factors for ovarian cancer is particularly important in the development of prevention and treatment strategies. 

Investigation of the latest disease burden, risk factors, and temporal trends of ovarian cancer is important for the reduction of its associated mortality globally. However, previous studies have mainly focused on single countries, or have used relatively old data [7,8,9]. The associations between common modifiable risk factors and ovarian cancer burden have not been comprehensively examined at a country level. This study aimed to investigate the most updated worldwide incidence and mortality, risk factors, and epidemiologic trend of ovarian cancer in different countries, regions, and age groups. 

## 2. Methods

### 2.1. Data Source

The global incidence and mortality rates of ovarian cancer for 185 countries in 2020 were retrieved from the Global Cancer Observatory (GLOBOCAN) database established by the International Agency for Research on Cancer (IARC, WHO) [1,10,11]. The gross domestic products (GDP) per capita and Human Development Index (HDI) were derived from the World Bank and United Nations, respectively. The Global Health Data Exchange (GHDx) [12] database was retrieved for the prevalence of smoking, alcohol use, unhealthy diet, physical inactivity, hypertension, diabetes, and lipid disorders in each country. Figures on the yearly incidence of ovarian cancer were collected from the Cancer Incidence in Five Continents I-X plus (CI5Plus) [13] for trend analysis. The CI5 is a global cancer database where the age and sex-specific cancer incidence are available for different countries to facilitate direct comparison between different countries [13]. Figures on mortality due to ovarian cancer were retrieved from the WHO Mortality Database [14]. The Nordic Cancer Registries (NORDCAN) [15,16] and the Surveillance, Epidemiology, and End Results (SEER) [17] were also accessed to obtain the most updated incidence and mortality of ovarian cancer for Northern European countries and the United States. A total of 48 countries/economies included for trend analysis were grouped into Asia, Oceania, North America, South America, Northern Europe, Western Europe, Southern Europe, Eastern Europe, and Africa. To confirm the data were correctly categorized, all extracted data of incidence and mortality rates for ovarian cancer in this study followed the International Classification of Disease and Related Health Problems’ 10th revision’s codes of ovarian cancer: “Malignant neoplasm of the ovary” (ICD-10, C56) [18]. To compare the data between different populations, all incidence and mortality rates were calculated using the age-standardized rates (ASRs) from the Segi–Doll standard population [19]. 

### 2.2. Statistical Analysis

Choropleth maps were plotted to present the global incidence and mortality of ovarian cancer. Associations between GDP per capita, HDI, the prevalence of smoking, alcohol use, unhealthy diet, physical inactivity, hypertension, diabetes, and lipid disorders and the burden of ovarian cancer were examined by univariable linear regression analysis. Beta coefficients (*β*) and the corresponding 95% confidence intervals (CI) were calculated from the regression. The *β* estimates refer to the degree of change in ASR of incidence or mortality of ovarian cancer per unit increase in the risk factors. For trend analysis of incidence and mortality rates for ovarian cancer, the data from the past 10 years were analyzed by joinpoint regression [20]. The ASRs and corresponding standard errors were transformed with the natural logarithm and calculated with binomial approximation. The annual percentage change (APC) was calculated by transforming ASRs and related SEs with joinpoint regression as percentage change rate by the assumption of a constant rate during the time interval [21]. For the calculation of the annual average percentage change (AAPC), the changepoint value was determined for the software to break the entire time interval. Each segment was weighted by the entire time interval proportion length. A maximum of one Joinpoint was used in the analysis. The temporal estimation outlined the incidence and mortality rates for ovarian cancer in the past decade. The AAPC was estimated to figure out the trend direction and degree by considering the slope and weighting of each segment. A positive value of AAPC indicated the upward inclination of the trend, and the significance of the trend was determined by its 95% C.I. (the trend was not significant if the 95% C.I. overlapped with zero) [21]. The epidemiological trends of ovarian cancer were analyzed in females aged 0–85+ years, and also in different age groups (≥50 years, <50 years, and <40 years). All *p*-values ≤ 0.05 were regarded as statistically significant.

## 3. Results

### 3.1. Global Incidence of Ovarian Cancer in 2020

**Figure 1:**
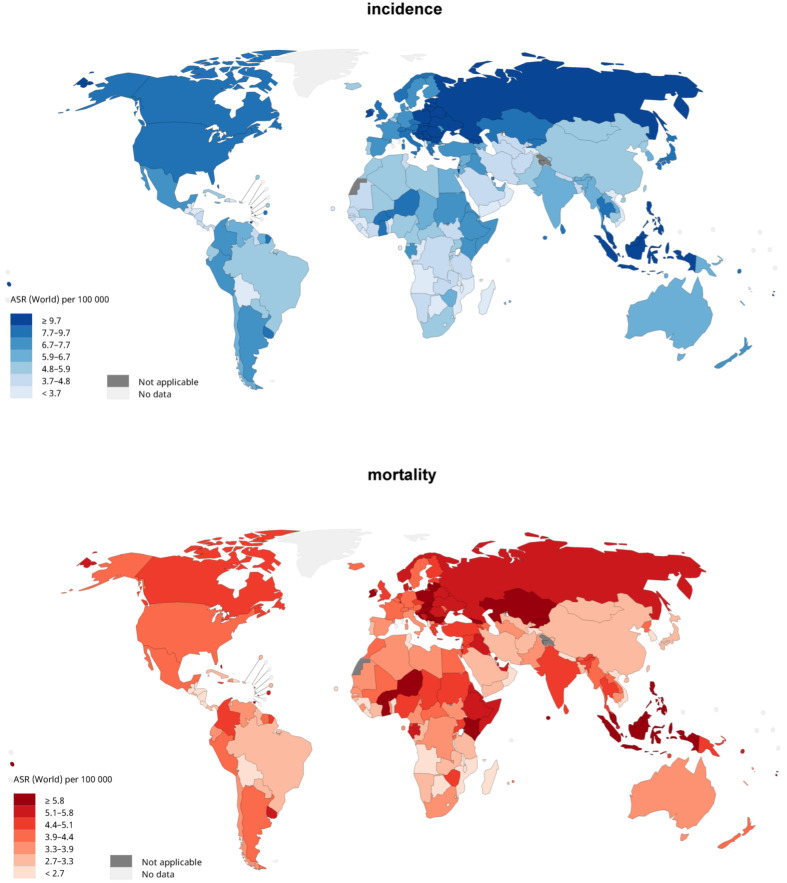
Global incidence and mortality of ovarian cancer, all ages, in 2020; Data source: https://gco.iarc.fr/today (accessed on 10 May 2021).

In 2020, a total of 313,959 new cases of ovarian cancer were recorded globally, with an ASR incidence of 6.6 per 100,000 (Figure 1). The highest incidence was found in Central and Eastern Europe (ASR = 10.7), followed by Northern Europe (ASR = 8.8), Polynesia (ASR = 8.8), North America (ASR = 8.1), and South East Asia (ASR = 8.1). The lowest incidence was observed in Central Africa (ASR = 4.4), the Caribbean (ASR = 4.6), and Southern Africa (ASR = 4.9). The highest incidence of ovarian cancer was observed in countries with a high-income level (ASR = 8.0), followed by countries with an upper–middle-income (ASR = 6.3), low–middle-income (ASR = 6.1), and low income (ASR = 5.3) levels. 

### 3.2. Global Mortality of Ovarian Cancer in 2020

In 2020, a total of 207,252 new deaths due to ovarian cancer were reported globally, with an ASR mortality of 4.2 per 100,000. The highest mortality was observed in Micronesia (ASR = 7.3), followed by Polynesia (ASR = 6.6), Central and Eastern Europe (ASR = 5.6), South East Asia (ASR = 5.2), and Melanesia (ASR = 5.2). The lowest mortality was observed in the Caribbean (ASR = 3.2), East Asia (ASR = 3.3), and Southern Africa (ASR = 3.3). The highest mortality was found in countries with a low–middle-income level (ASR = 4.3), followed by countries with high-income level (ASR = 4.1), low-income level (ASR = 4.1), and upper–middle-income level (ASR = 3.9).

### 3.3. Associations between Risk Factors and Incidence

**Figure 2:**
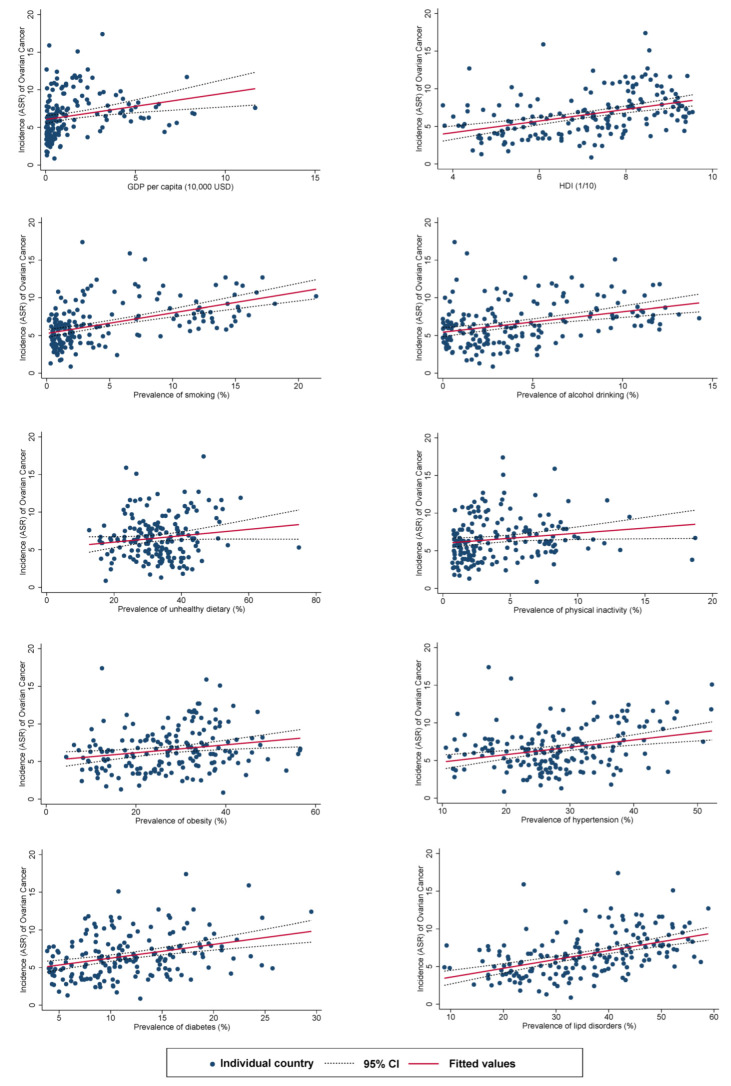
Associations between risk factors and the incidence of ovarian cancer.

The associations between risk factors and the incidence of ovarian cancer are presented in Figure 2. Higher ASRs in the incidence of ovarian cancer was associated with a higher GDP per capita (*β* = 0.35, 95% C.I. 0.14 to 0.56, *p* = 0.001), HDI (*β* = 0.77, 95% C.I. 0.52 to 1.03, *p* < 0.001), and a higher prevalence of smoking (*β* = 0.27, 95% C.I. 0.20 to 0.35, *p* < 0.001), alcohol use (*β* = 0.27, 95% C.I. 0.16 to 0.38, *p* < 0.001), physical inactivity (*β* = 0.14, 95% C.I. 0.01 to 0.26, *p* = 0.036), obesity (*β* = 0.05, 95% C.I. 0.02 to 0.09, *p* = 0.005), hypertension (*β* = 0.10, 95% C.I. 0.05 to 0.14, *p* < 0.001), diabetes (*β* = 0.18, 95% C.I. 0.11 to 0.26, *p* < 0.001), and lipid disorders (*β* = 0.12, 95% C.I. 0.09 to 0.15, *p* < 0.001).

### 3.4. Associations between Risk Factors and Mortality

**Figure 3:**
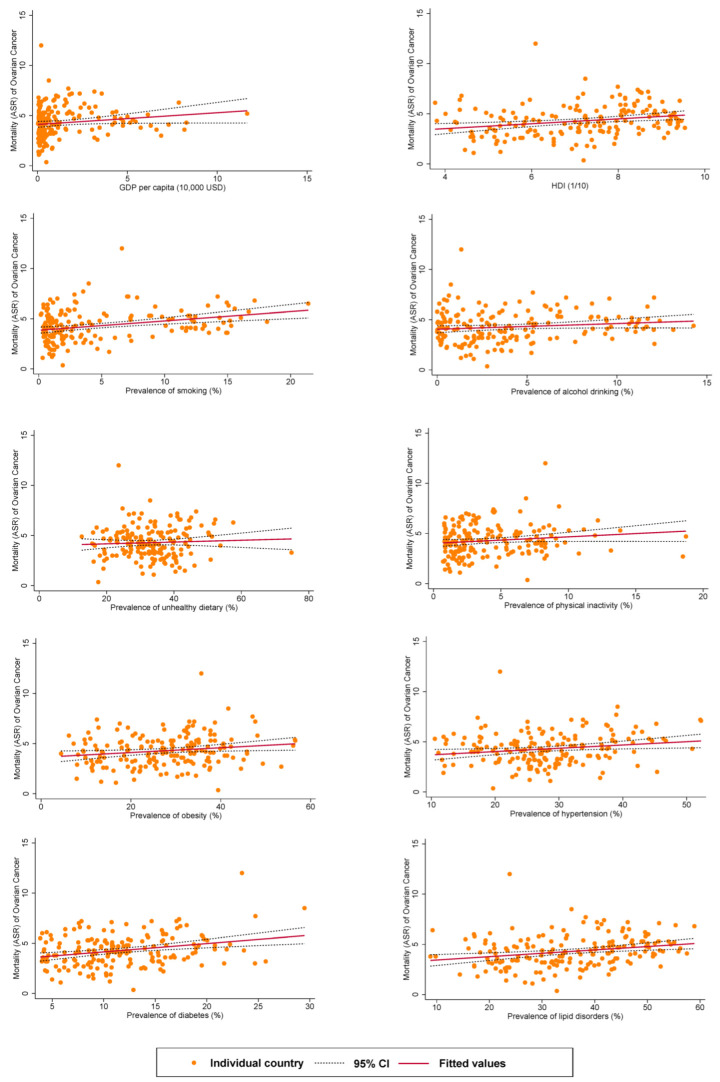
Associations between risk factors and mortality of ovarian cancer.

The associations between risk factors and mortality related to ovarian cancer are presented in Figure 3. Higher ASRs of mortality of ovarian cancer was associated with a higher HDI (*β* = 0.24, 95% C.I. 0.09 to 0.39, *p* < 0.001), and higher prevalence of smoking (*β* = 0.09, 95% C.I. 0.05 to 0.14, *p* < 0.001), obesity (*β* = 0.02, 95% C.I. 0.003 to 0.04, *p* = 0.023), hypertension (*β* = 0.03, 95% C.I. 0.01 to 0.06, *p* = 0.015), diabetes (*β* = 0.08, 95% C.I. 0.04 to 0.13, *p* < 0.001), and lipid disorders (*β* = 0.03, 95% C.I. 0.01 to 0.05, *p* = 0.001). 

### 3.5. Temporal Trends of Ovarian Cancer

The incidence and mortality trends of ovarian cancer between 1980 to 2018 are displayed in Figure S1. The joinpoint regression analysis results are displayed in Figure S2. Overall, there was a decreasing trend in the incidence and mortality rates of ovarian cancer globally. However, a substantial increase in incidence of ovarian cancer was observed in younger females. 

### 3.6. Overall Incidence Trends of Ovarian Cancer

**Figure 4:**
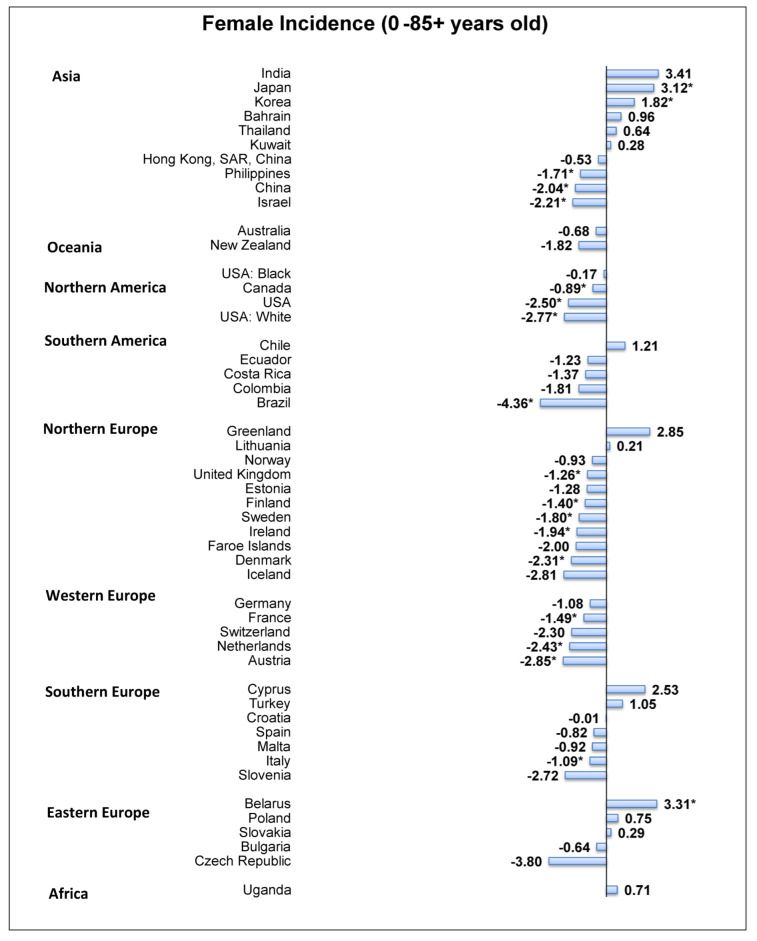
AAPC of incidence of ovarian cancer aged 0–85+. * Statistically significant.

Of the 48 countries, 17 showed a decreased trend of ovarian cancer incidence from the past ten years up to 2018 (Figure 4). Among these countries, the lowest AAPC was from Brazil (AAPC = −4.36, 95% C.I. −6.37 to −2.20, *p* = 0.002) followed by the Czech Republic (AAPC = −3.80, 95% C.I. −5.86 to −1.70, *p* < 0.001), Austria (AAPC = −2.85, 95% C.I. −3.92 to −1.76, *p* < 0.001), the United States (AAPC = −2.50, 95% C.I. −3.45 to −1.55, *p* < 0.001), and the Netherlands (AAPC = −2.43, 95% C.I. −3.93 to −0.91, *p* = 0.006). By contrast, three countries had an increase in ovarian cancer incidence, including Belarus (AAPC = 3.31, 95% C.I. 2.38 to 4.26, *p* < 0.001), Japan (AAPC = 3.12, 95% C.I. 2.12 to 4.13, *p* < 0.001), and Korea (AAPC = 1.82, 95% C.I. 0.50 to 3.16, *p* = 0.013). 

### 3.7. Overall Mortality Trends of Ovarian Cancer

**Figure 5:**
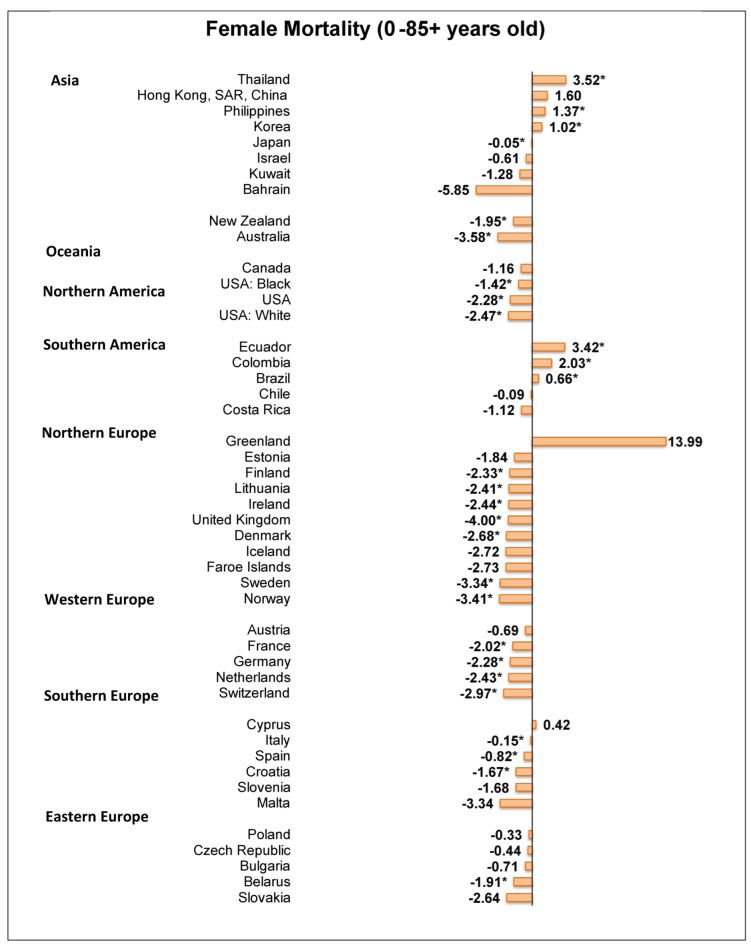
AAPC of mortality of ovarian cancer aged 085+. * Statistically significant.

Of the 48 countries, 21 showed a decrease in the mortality of ovarian cancer (Figure 5). Among these countries, the lowest AAPC was from Australia (AAPC = −3.58, 95% C.I. −5.28 to −1.86, *p* < 0.001), followed by Norway (AAPC = −3.41, 95% C.I −4.42 to −0.24, *p* < 0.001), Sweden (AAPC = −3.34, 95% C.I −5.26 to −1.38, *p* = 0.005), Switzerland (AAPC = −2.97, 95% C.I. −4.52 to −1.39, *p* = 0.003), and Denmark (AAPC = −2.68, 95% C.I. −4.43 to −0.89, *p* = 0.009). By contrast, six countries had an increase in mortality due to ovarian cancer, including Thailand (AAPC = 3.52, 95% C.I. 2.77 to 4.27, *p* < 0.001), Ecuador (AAPC = 3.42, 95% C.I. 1.41 to 5.46, *p* = 0.004), Colombia (AAPC = 2.03, 95% C.I. 1.30 to 2.76, *p* < 0.001), the Philippines (AAPC = 1.37, 95% C.I. 0.71 to 2.03, *p* = 0.001), Korea (AAPC = 1.02, 95% C.I. 0.10 to 1.95, *p* = 0.033), and Brazil (AAPC = 0.66, 95% C.I. 0.19 to 1.13, *p* = 0.011).

### 3.8. Incidence Trends of Ovarian Cancer in Females Aged ≥ 50

In the 48 countries, 21 showed a decrease in incidence of ovarian cancer (Figure S3), with Brazil having highest decline in incidence (AAPC = −4.02, 95% C.I. −6.56 to −1.41, *p* = 0.008), followed by Slovenia (AAPC = −3.86, 95% C.I. −6.51 to −1.14, *p* = 0.012), the United States (AAPC = −3.01, 95% C.I. −4.23 to −2.22, *p* < 0.001), Switzerland (AAPC = −2.93, 95% C.I. −5.36 to −0.44, *p* = 0.027), and Austria (AAPC = −2.60, 95% C.I. −3.41 to −1.78, *p* < 0.001). On the contrary, only two countries had an increase in the incidence of ovarian cancer, including Japan (AAPC = 2.35, 95% C.I. 1.15 to 3.55, *p* = 0.002), and Belarus (AAPC = 1.88, 95% C.I. 1.21 to 2.54, *p* < 0.001).

### 3.9. Incidence Trends of Ovarian Cancer in Females Aged < 50

The decreasing trend was much less evident in females aged < 50 (Figure S4). Only four countries demonstrated a decrease in the incidence of ovarian cancer, including the Czech Republic (AAPC = −6.47, 95% C.I. −9.10 to −3.77, *p* < 0.001), Brazil (AAPC = −5.18, 95% C.I. −7.97 to −2.30, *p* = 0.003), Austria (AAPC = −3.43, 95% C.I. −6.27 to −0.50, 0.027), and the Netherlands (AAPC = −2.20, 95% C.I. −3.50 to −0.87, *p* = 0.005). By contrast, three countries showed an increase in the incidence of ovarian cancer including Belarus (AAPC = 5.61, 95% C.I. 2.90 to 8.39, *p* = 0.001), Japan (AAPC = 4.06, 95% C.I. 2.06 to 6.10, *p* = 0.001), and Korea (AAPC = 2.36, 95% C.I. 0.49 to 4.26, *p* = 0.019).

### 3.10. Incidence Trends of Ovarian Cancer in Females Aged < 40

Notably, no country showed a decreasing trend of ovarian cancer in females aged < 40 (Supplementary Figure S5). However, there were four countries showing an increasing trend of ovarian cancer, including India (AAPC = 6.89, 95% C.I. 0.95 to 13.18, *p* = 0.028), Belarus (AAPC = 6.69, 95% C.I. 2.48 to 11.08, *p* = 0.002), African Americans in the United States: (AAPC = 4.90, 95% C.I. 0.50 to 9.48, *p* = 0.033), and Japan (AAPC = 4.00, 95% C.I. 0.88 to 7.21, *p* = 0.018).

## 4. Discussion

### 4.1. Summary of Main Findings

There were several major findings: (1) the highest mortality rates due to ovarian cancer were observed in low–middle-income countries, and its incidence was highest in countries with high-income levels; (2) higher incidence of ovarian cancer was associated with a higher GDP per capita, HDI, prevalence of smoking, alcohol use, physical inactivity, obesity, hypertension, diabetes, and lipid disorders; (3) although there was an overall decreasing trend of incidence and mortality of ovarian cancer over the past decade, a substantial increase in incidence was observed in younger females. 

### 4.2. Explanation of Findings and Relationship with Literature

The higher mortality rates of ovarian cancer in countries with lower-income levels found in this study are of concern. Although the highest incidence of ovarian cancer was observed in high-income countries, mortality was highest in countries with a lower-income level. In this analysis, incidence of ovarian cancer was associated with GDP per capita and HDI at the country level. The highest incidence rates in high-income countries may be driven by the higher prevalence of environmental, lifestyle, metabolic risk factors, nulliparity, menopausal hormone therapy use, and familial predisposition [22]. However, the largest mortality and mortality-to-incidence ratio were found in countries with lower-incomes. This could be due to the suboptimal resources available for early detection, treatment, and surveillance for cancer patients in these regions [23]. Based on this analysis, mortality rates due to ovarian cancer have been increasing for the past decade in some less developed countries, including Thailand, Ecuador, Colombia, the Philippines, and Brazil. These trends will likely continue in the future, suggesting that ovarian cancer is a significant health threat for these populations. More resources and targeted prevention strategies for ovarian cancer are needed in countries undergoing rapid economic development.

This study identified some preventable lifestyle and metabolic risk factors associated with the burden of ovarian cancer at a country level. These findings are generally consistent with previous observational studies using data from individuals. For example, a study using individual participant data for 28,114 women with ovarian cancer found that women who had smoked in the past had a 6% higher risk of ovarian cancer than those who had never smoked [24]. Consumption of at least one glass of wine per day, compared to no wine in the year before baseline, was associated with a 57% increased risk of developing ovarian cancer [25]. Women with the highest level of physical activity had an odds ratio of 0.73 (95% CI 0.56, 0.94) for ovarian cancer, compared with women with the lowest level of activity [26]. Overweight women had a 7% higher risk of ovarian cancer, and obese women had a 28% higher risk [27]. The presence of metabolic syndrome was also associated with ovarian cancer (relative risk (RR) = 3.42; 95% CI, 2.84 to 4.11) [28]

An overall decreasing trend of ovarian cancer was found, yet an increase in incidence was observed among younger females in some countries. Several factors may have contributed to this favorable trend, including the declining smoking prevalence, increasing use of oral contraceptives, and improvement in diagnosis and treatment. From 1980 to 2012, the prevalence of smoking among females decreased in North America, Australia, and New Zealand [29]. The use of oral contraceptives was usually higher in Western countries, which may have a protective effect for ovarian cancer [30]. The declining mortality rates for ovarian cancer might be attributed to the recent improvement in diagnosis and treatment. However, the increasing trend of incidence in younger females might be driven by the increasing prevalence of obesity, metabolic syndrome, estrogen exposure, and nulliparity among younger females. From 1985–2014, younger subjects (aged 15–40 years) showed a more drastic rise in prevalence (16.3 to 33.9%) than subjects aged  >40 years (43.6 to 57.9%) [31]. The prevalence of metabolic syndrome also rose more rapidly in women and the younger population from 1991–2015 [32]. The increasing incidence may also be related to the rising presence of BRCA gene mutations in younger females. According to a recent study, rates of genetic testing for abnormal BRCA1 and BRCA2 genes have increased in females aged 40 or younger, which will increase their risk of ovarian cancer [33]

## 5. Limitation

Several limitations need to be mentioned in the current study. Firstly, there could be under-reporting of the incidence and mortality of ovarian cancer in the less developed countries with the underdevelopment in infrastructure and mechanism of cancer registries. Secondly, figures might have been overestimated for some countries as their incidence and mortality rates were represented by cancer registries in major cities. Thirdly, a direct comparison of the temporal trends of ovarian cancer between different countries may be problematic, as the cancer registries’ infrastructure may have changed over time. Nevertheless, this limitation is of less concern when comparing the trends of ovarian cancer between different age groups within the same country. Lastly, there was a lack of analysis on the incidence, mortality, risk factors, and epidemiological trends of ovarian cancer by different histological subtypes. This information is also important for the prevention of ovarian cancer as the epidemiological patterns could vary by different histological subtypes of ovarian cancer.

## 6. Conclusions

The incidence of ovarian cancer has been increasing substantially among younger females, probably caused by the increasing prevalence of obesity, metabolic syndrome, estrogen exposure and nulliparity. These trends are expected to continue due to economic growth and lifestyle transitions, particularly among countries with lower-income levels. Intensive lifestyle modifications are warranted, especially for the populations at high risk for ovarian cancer, including smoking cessation, alcohol use reduction, physical activity, weight control, and treatment of metabolic diseases. It is also important to improve early diagnosis, treatment, surveillance, and quality of life for patients with ovarian cancer. Longitudinal studies are needed to further confirm the drivers of these epidemiologic trends and provide more insight into the specific etiology and prognosis of ovarian cancer by histological subtypes.

## Data Availability

The data used for the analyses are publicly available from the World Health Organization websites (https://gco.iarc.fr/, https://apps.who.int/gho/data/node.main, accessed on 10 May 2021).

## Supplementary Materials

The following are available online at https://www.mdpi.com/article/10.3390/cancers14092230/s1, Figure S1: The plots of incidence and mortality trends for each country, Figure S2: The graphs of the joinpoint regression output, Figure S3: AAPC of incidence of ovarian cancer aged 50 years and older, Figure S4: AAPC of incidence of ovarian cancer aged <50 years old, Figure S5: AAPC of incidence of ovarian cancer aged <40 years old.

## Abbreviations

AAPC	Average annual percent change
APC	Annual percentage change
ASR	Age-standardized rates
CI	Confidence interval
CI5	Cancer Incidence in Five Continents
GDP	Gross domestic products
GHDx	Global Health Data Exchange
HDI	Human Development Index
IARC	International Agency for Research on Cancer
NORDCAN	Nordic Cancer Registries
RR	Relative risk
SEER	Surveillance, Epidemiology, and End Results
