# Supplementary material for: Worldwide Burden, Risk Factors, and Temporal Trends of Ovarian Cancer: A Global Study
Source: Cancers (Basel). 2022 Apr 29;14(9):2230. doi: 10.3390/cancers14092230 (PMC9102475; doi:10.3390/cancers14092230)

Supplementary Legends

Supplementary Figure S1: The plots of incidence and mortality trends for each country

Supplementary Figure S2: The graphs of the joinpoint regression output

Supplementary Figure S3. AAPC of incidence of ovarian cancer aged 50 years and older

Supplementary Figure S4. AAPC of incidence of ovarian cancer aged < 50 years old

Supplementary Figure S5. AAPC of incidence of ovarian cancer aged < 40 years old

Supplementary Figure S1: The plots of incidence and mortality trends for each country

Female

Asia

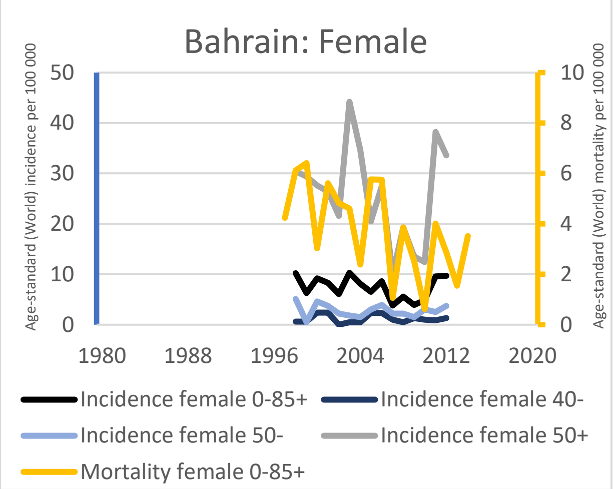

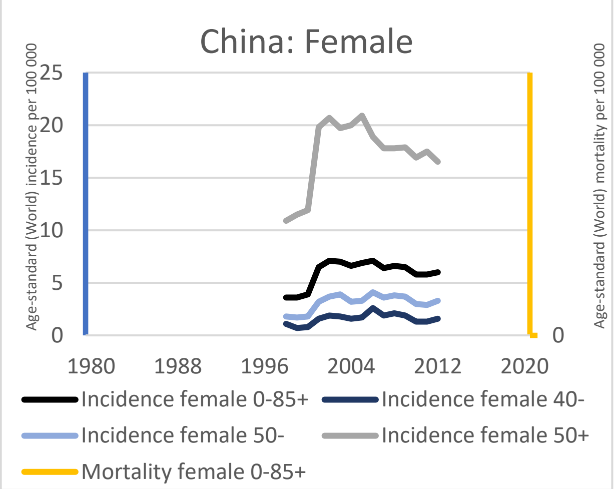

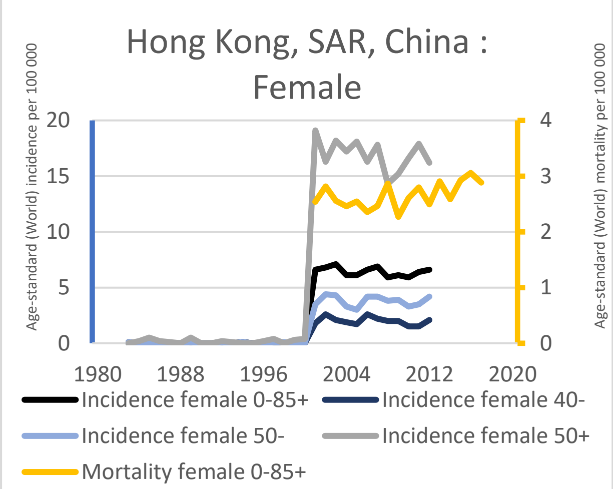

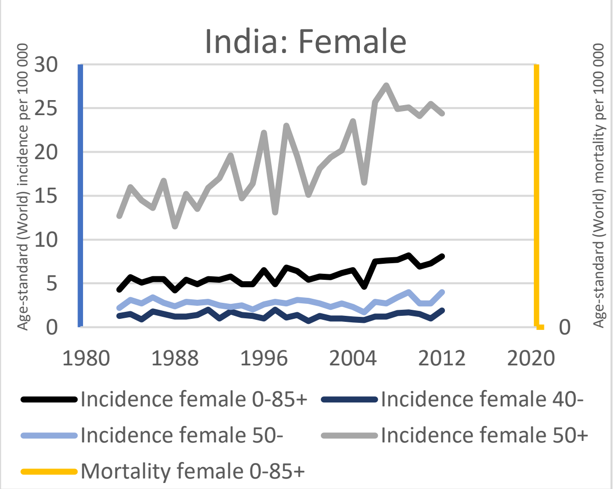

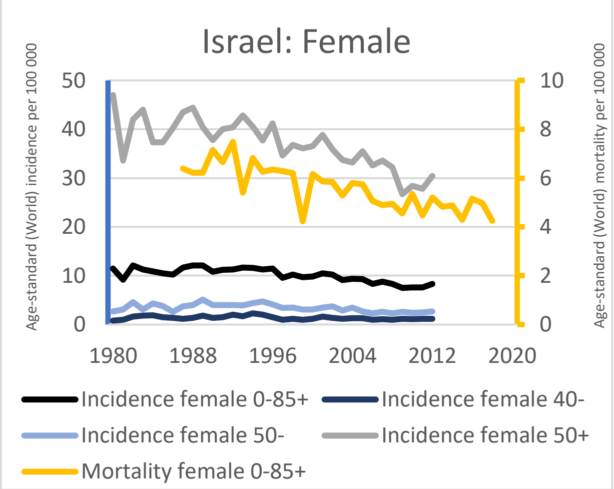

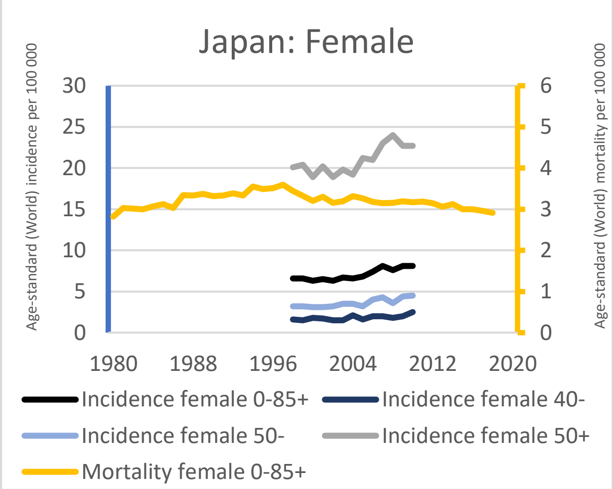

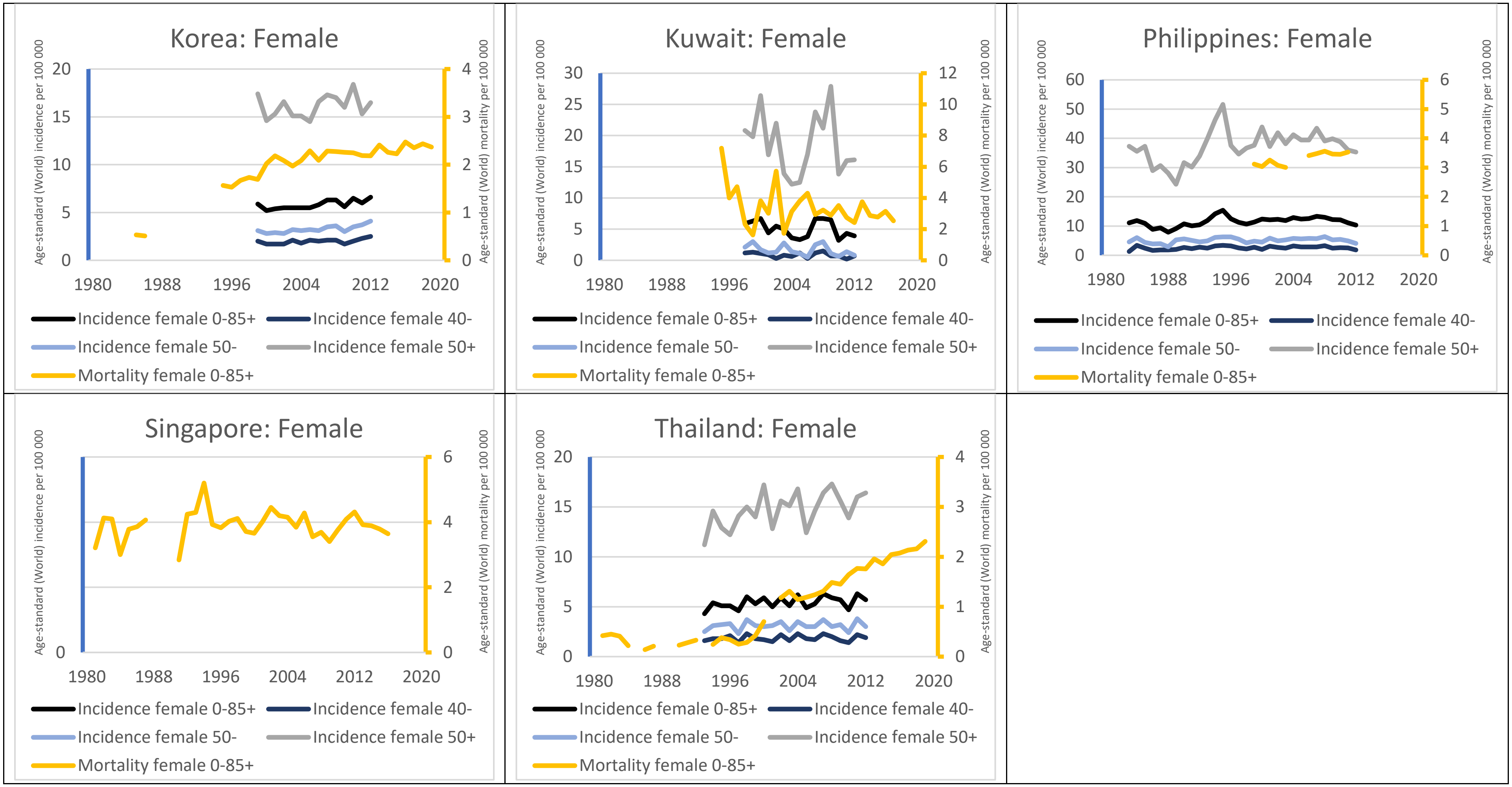
Korea: Female
Age-standard (World) incidence per 100 000
Age-standard (World) mortality per 100 000
0
5
10
15
20
0
1
2
3
4
1980
1988
1996
2004
2012
2020
Incidence female 0-85+
Incidence female 40-
Incidence female 50-
Incidence female 50+
Mortality female 0-85+
Kuwait: Female
Age-standard (World) incidence per 100 000
Age-standard (World) mortality per 100 000
0
5
10
15
20
25
30
0
2
4
6
8
10
12
1980
1988
1996
2004
2012
2020
Incidence female 0-85+
Incidence female 40-
Incidence female 50-
Incidence female 50+
Mortality female 0-85+
Philippines: Female
Age-standard (World) incidence per 100 000
Age-standard (World) mortality per 100 000
0
10
20
30
40
50
60
0
1
2
3
4
5
6
1980
1988
1996
2004
2012
2020
Incidence female 0-85+
Incidence female 40-
Incidence female 50-
Incidence female 50+
Mortality female 0-85+
Singapore: Female
Age-standard (World) incidence per 100 000
Age-standard (World) mortality per 100 000
0
0
2
4
6
1980
1988
1996
2004
2012
2020
Incidence female 0-85+
Incidence female 40-
Incidence female 50-
Incidence female 50+
Mortality female 0-85+
Thailand: Female
Age-standard (World) incidence per 100 000
Age-standard (World) mortality per 100 000
0
5
10
15
20
0
1
2
3
4
1980
1988
1996
2004
2012
2020
Incidence female 0-85+
Incidence female 40-
Incidence female 50-
Incidence female 50+
Mortality female 0-85+

Oceania

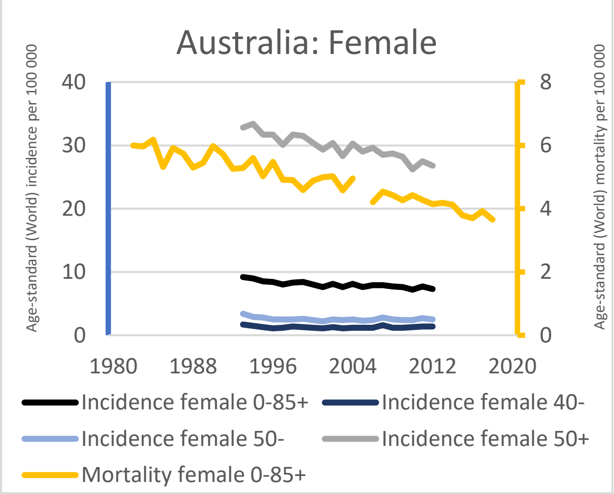

Australia: Female
Age-standard (World) incidence per 100 000
Age-standard (World) mortality per 100 000
40
30
20
10
0
8
6
4
2
0
1980
1988
1996
2004
2012
2020
Incidence female 0-85+
Incidence female 40-
Incidence female 50-
Incidence female 50+
Mortality female 0-85+

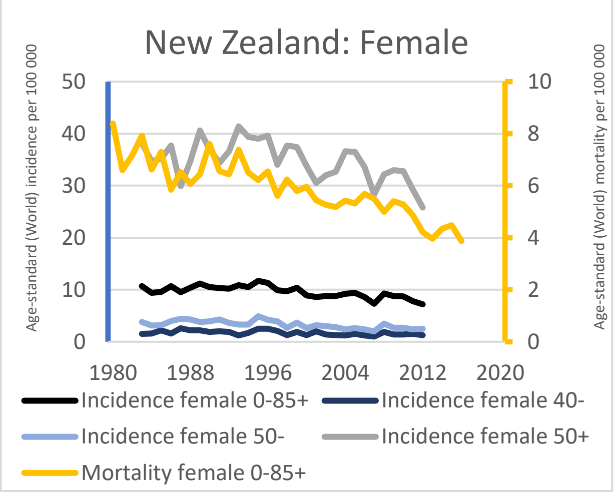

New Zealand: Female
Age-standard (World) incidence per 100 000
Age-standard (World) mortality per 100 000
50
40
30
20
10
0
10
8
6
4
2
0
1980
1988
1996
2004
2012
2020
Incidence female 0-85+
Incidence female 40-
Incidence female 50-
Incidence female 50+
Mortality female 0-85+

Northern America

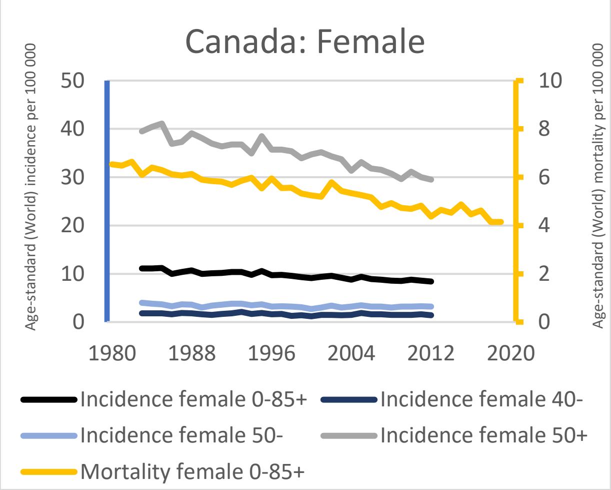

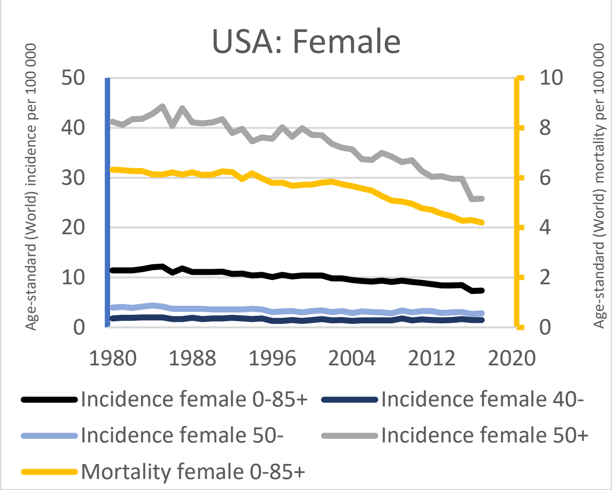

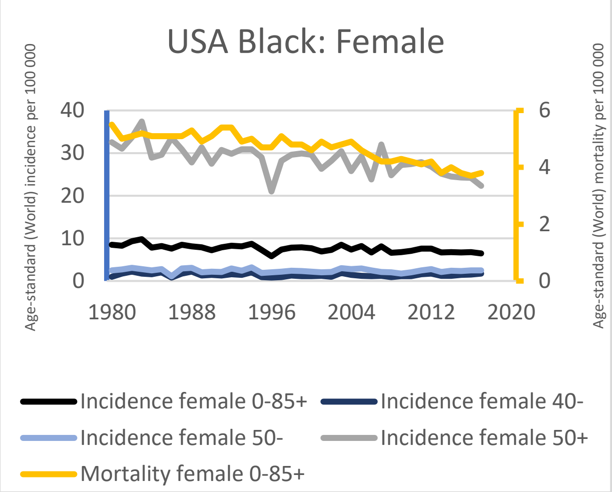

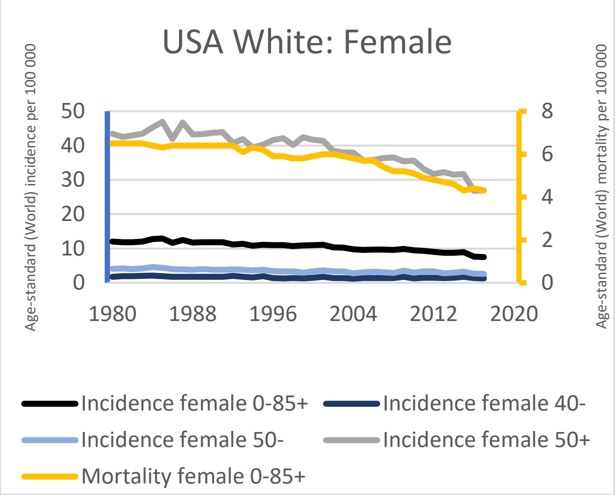

Southern America

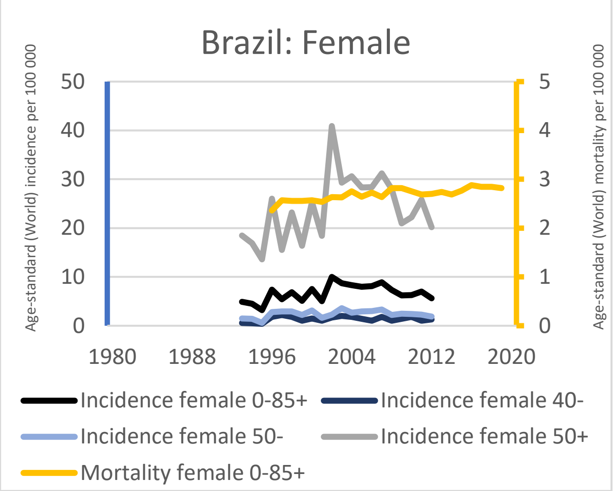

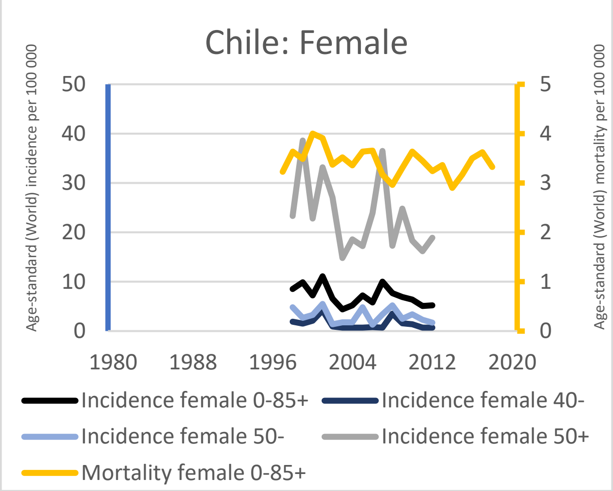

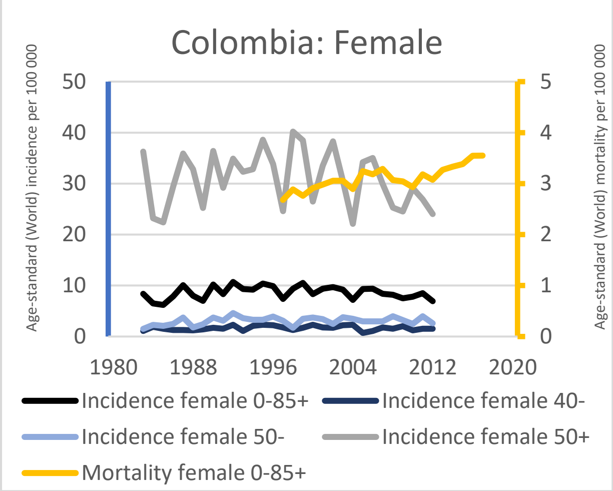

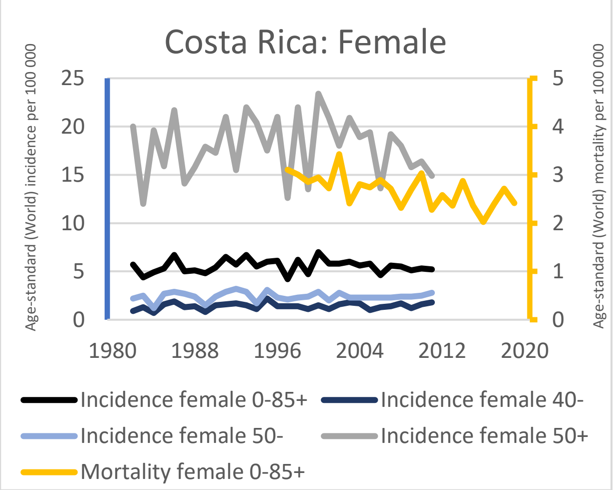

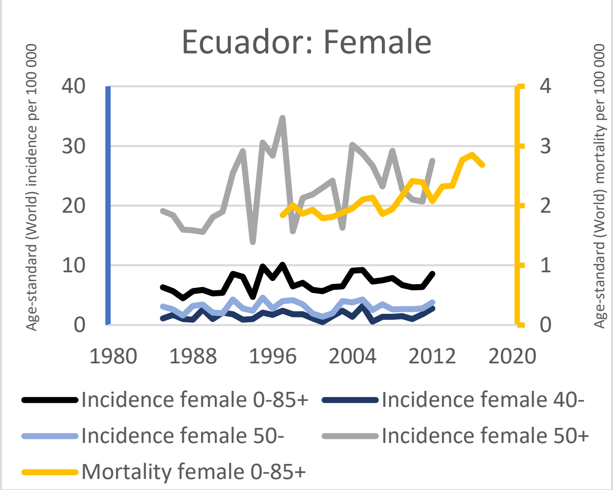

Northern Europe

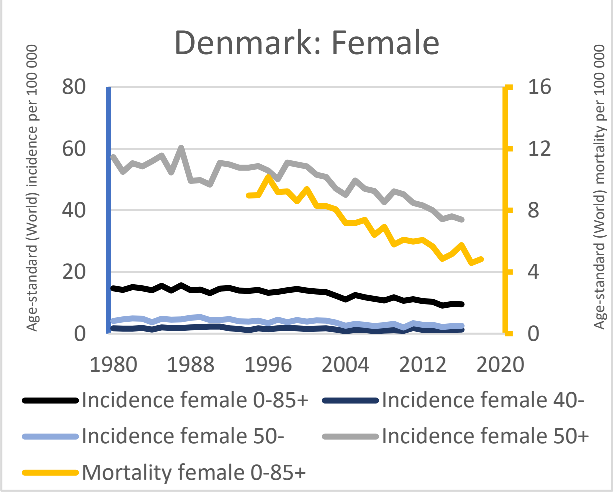

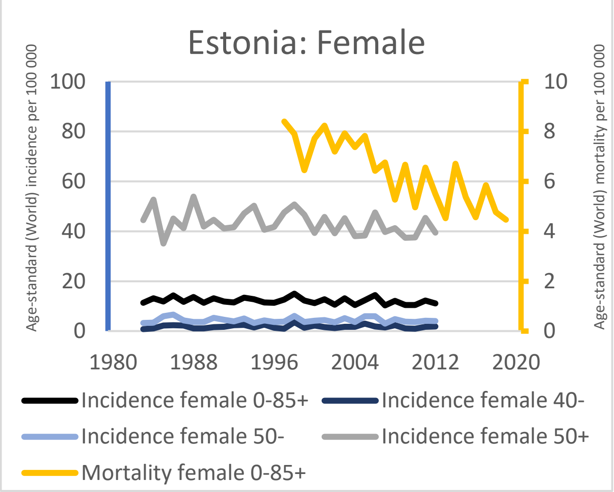

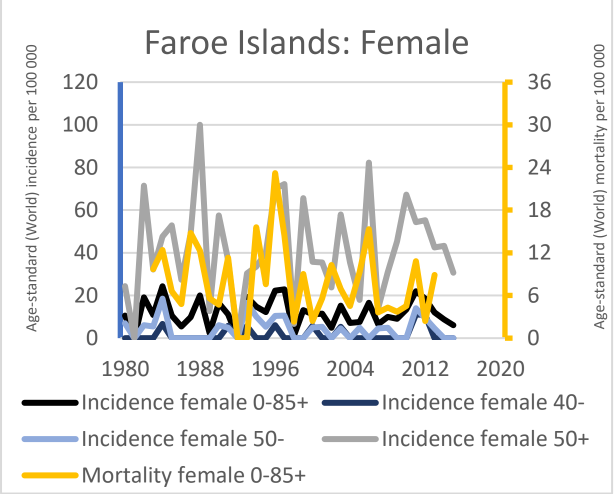

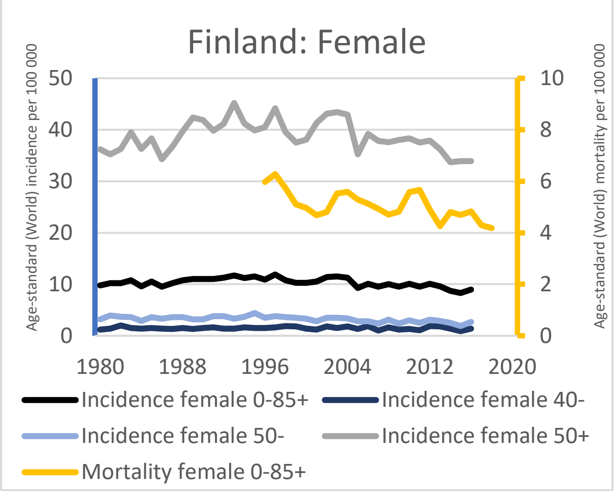

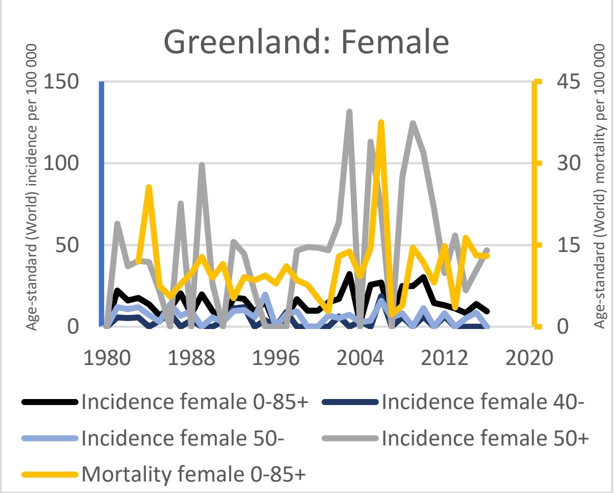

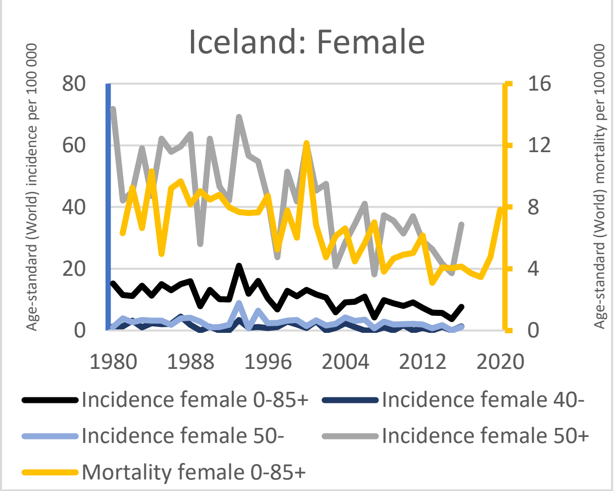

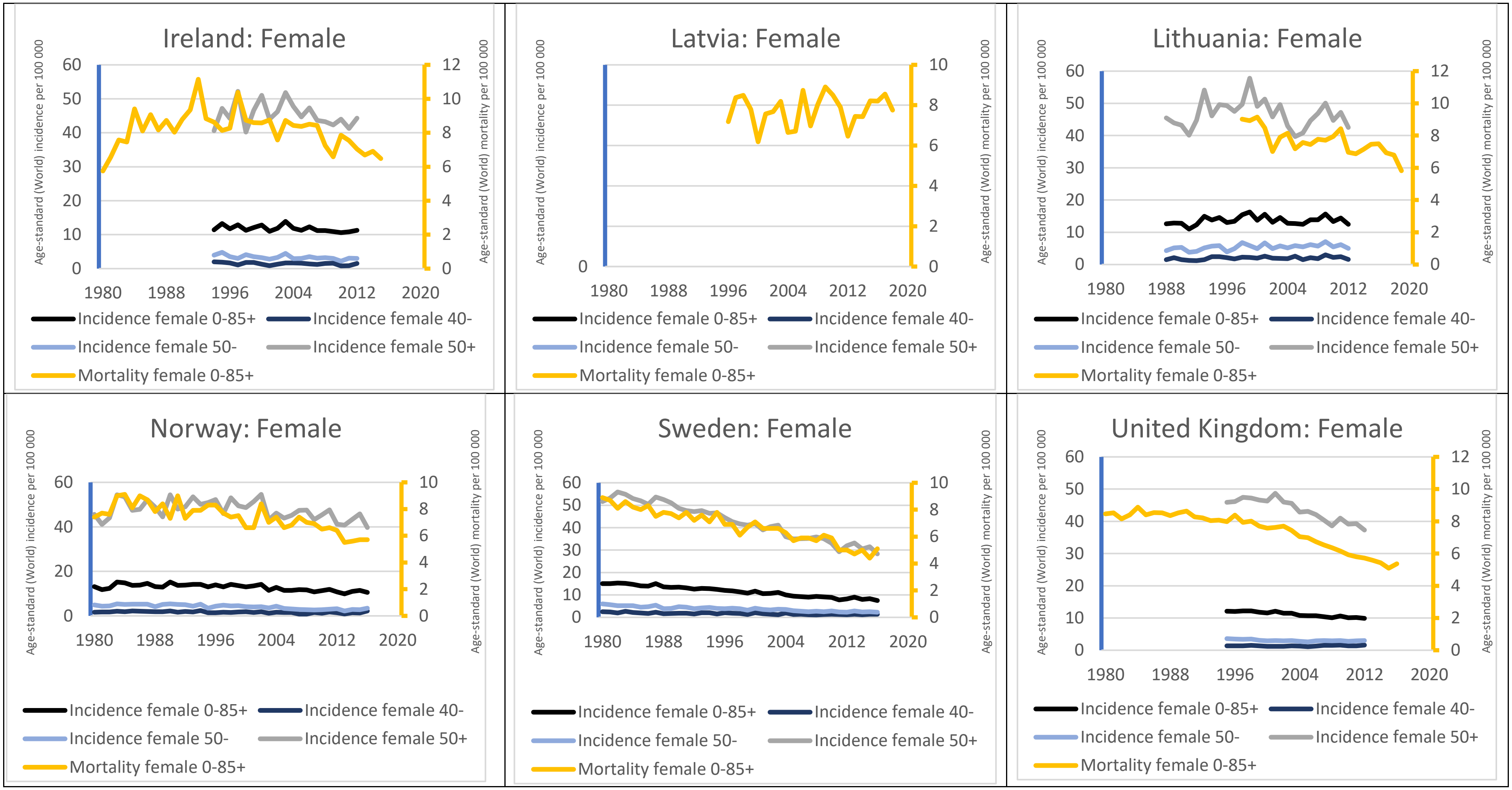

Ireland: Female
Latvia: Female
Lithuania: Female
Norway: Female
Sweden: Female
United Kingdom: Female
Age-standard (World) incidence per 100 000
Age-standard (World) mortality per 100 000
1980
1988
1996
2004
2012
2020
Incidence female 0-85+
Incidence female 40-
Incidence female 50-
Incidence female 50+
Mortality female 0-85+

Western Europe

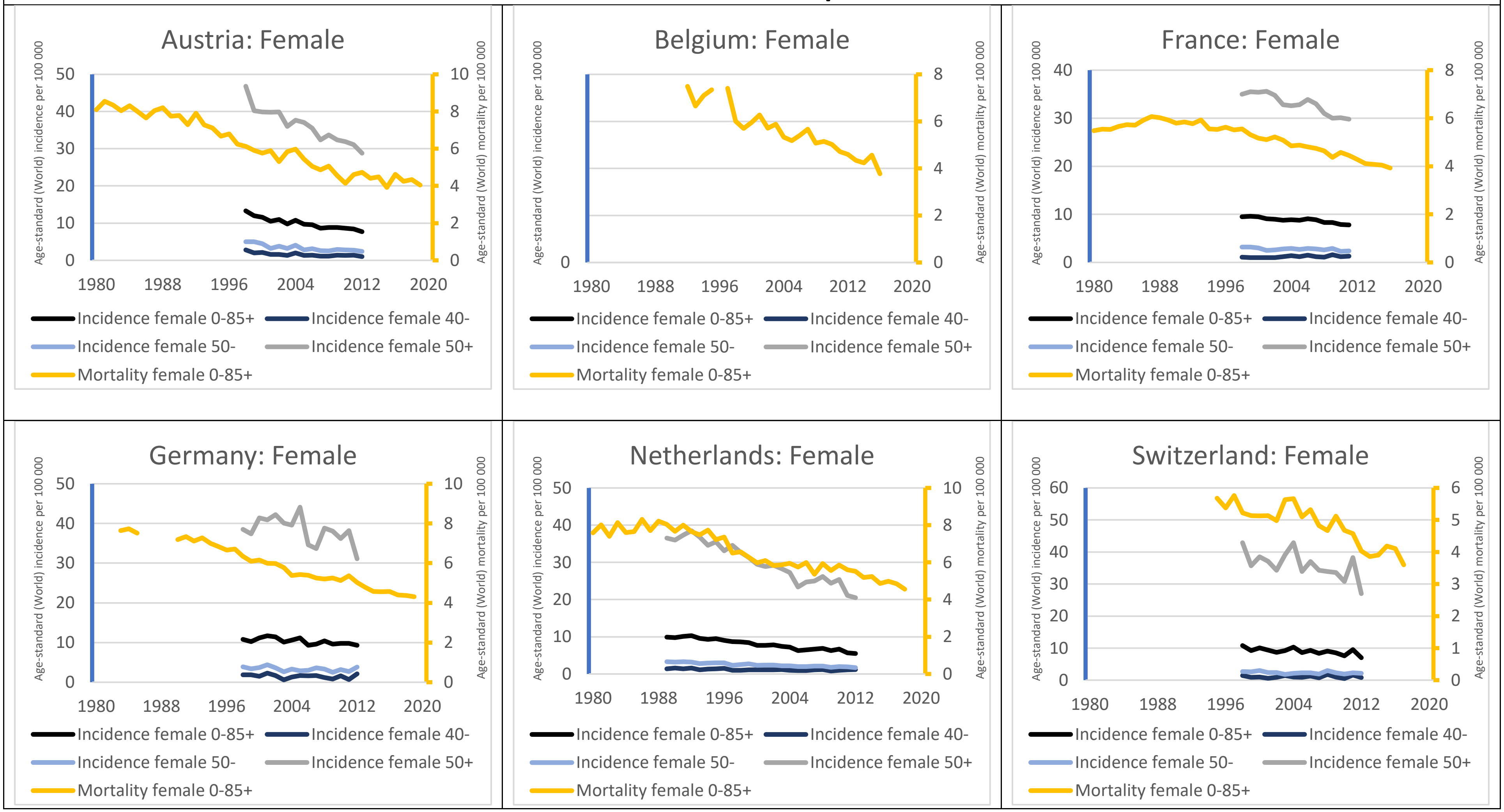

Southern Europe

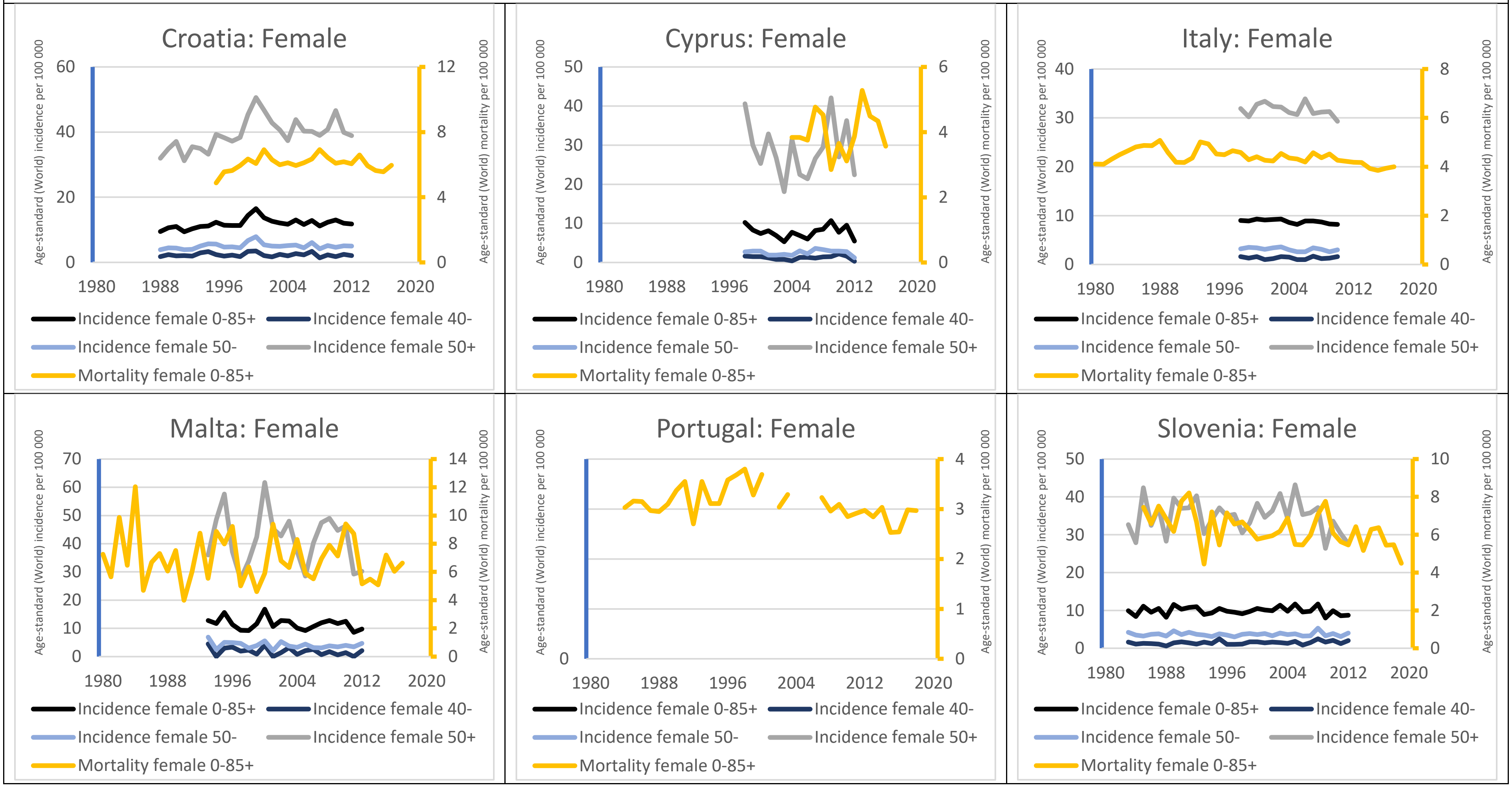

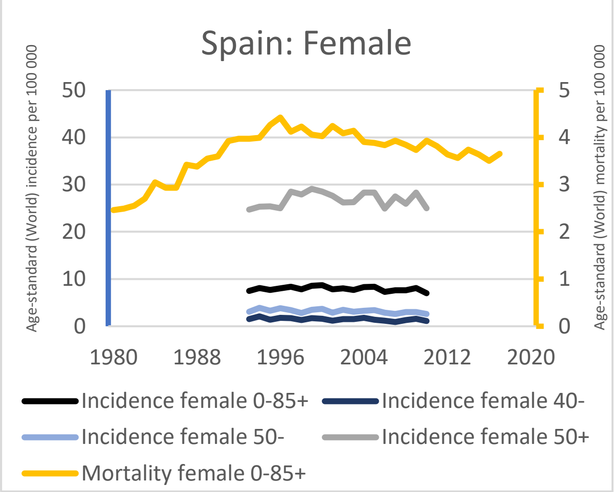
Spain: Female
Age-standard (World) incidence per 100 000
50
40
30
20
10
0
Age-standard (World) mortality per 100 000
5
4
3
2
1
0
1980
1988
1996
2004
2012
2020
Incidence female 0-85+
Incidence female 40-
Incidence female 50-
Incidence female 50+
Mortality female 0-85+

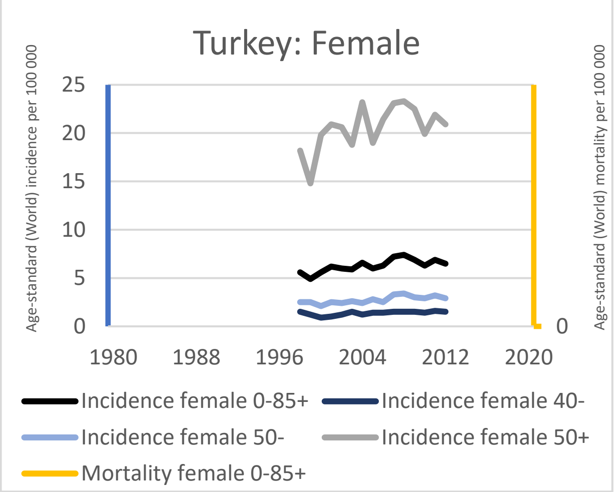
Turkey: Female
Age-standard (World) incidence per 100 000
25
20
15
10
5
0
Age-standard (World) mortality per 100 000
0
1980
1988
1996
2004
2012
2020
Incidence female 0-85+
Incidence female 40-
Incidence female 50-
Incidence female 50+
Mortality female 0-85+

Eastern Europe

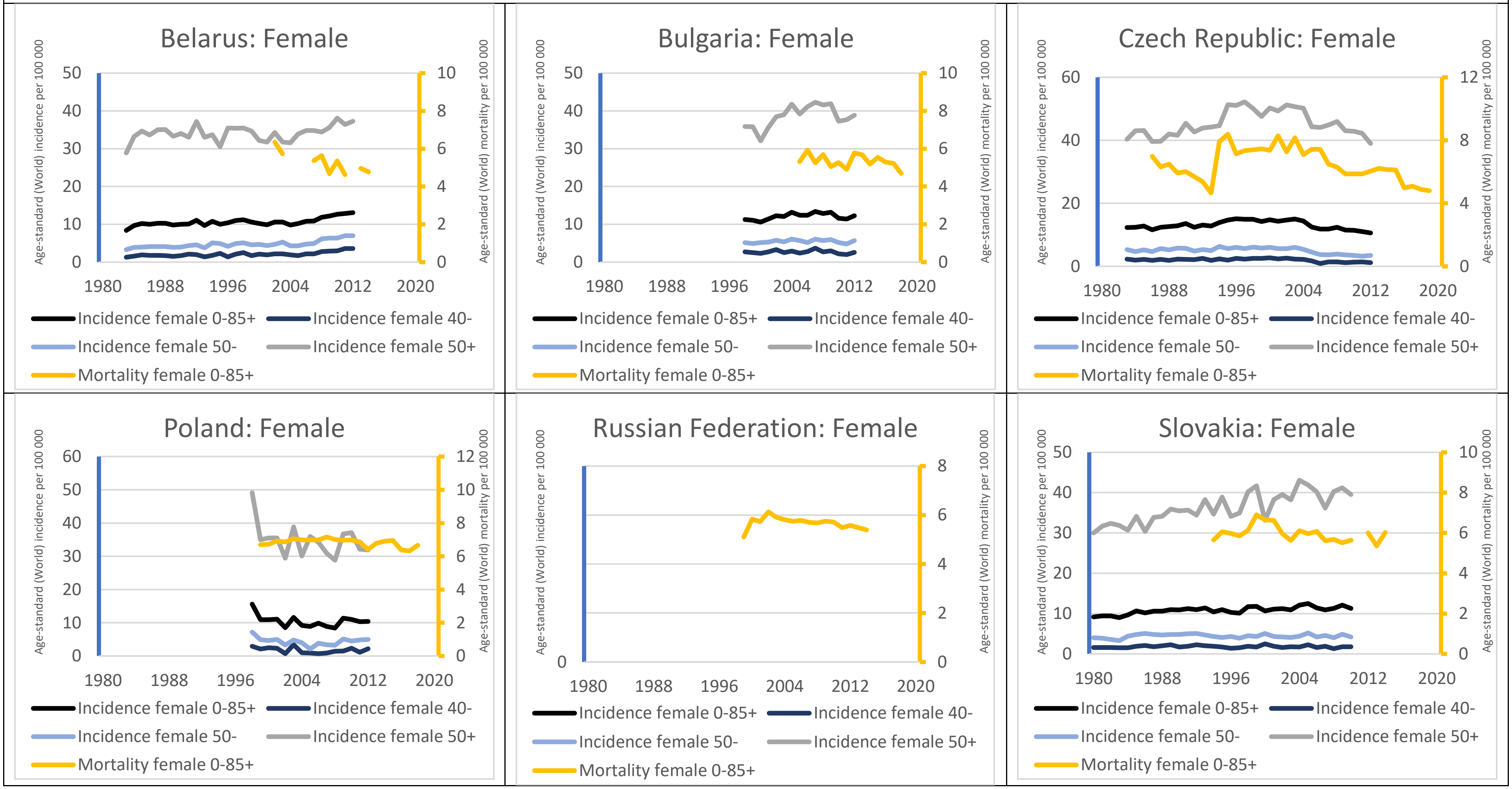

Africa

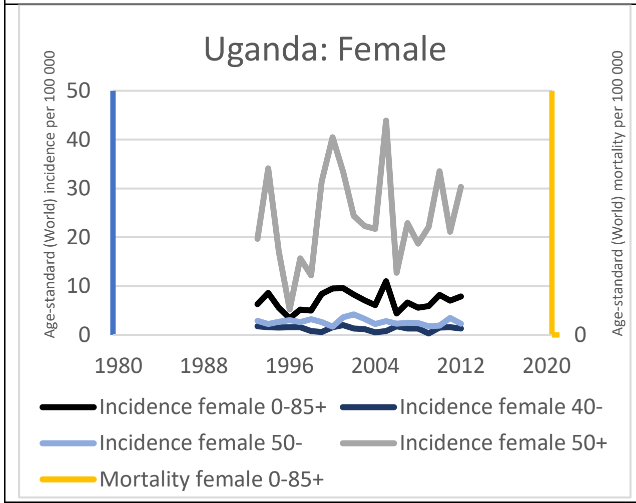
Uganda: Female
Age-standard (World) incidence per 100 000
Age-standard (World) mortality per 100 000
50
40
30
20
10
0
0
1980
1988
1996
2004
2012
2020
Incidence female 0-85+
Incidence female 40-
Incidence female 50-
Incidence female 50+
Mortality female 0-85+

Supplementary Figure S2: The graphs of the joinpoint regression output a.) Incidence female all ages

Asia

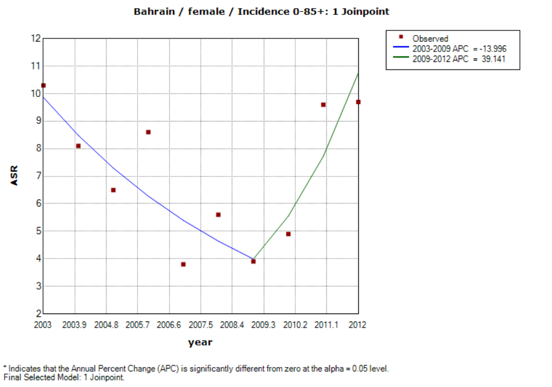

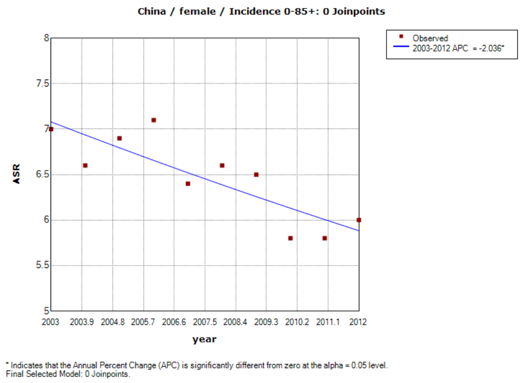

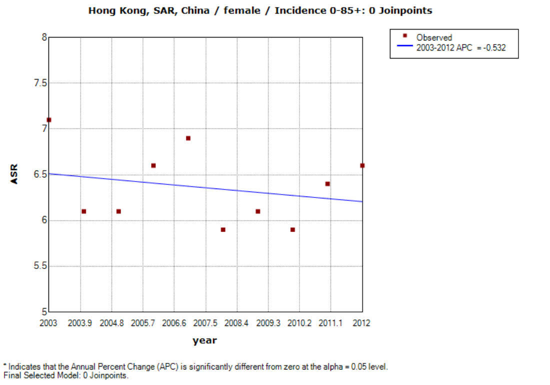

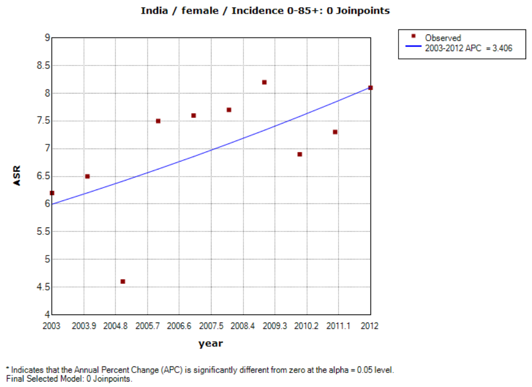

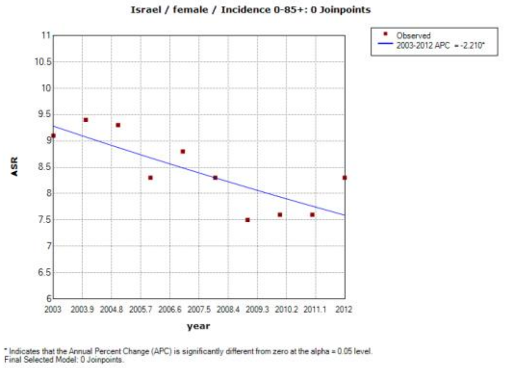

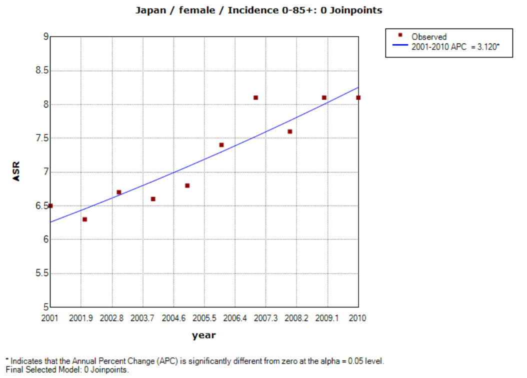

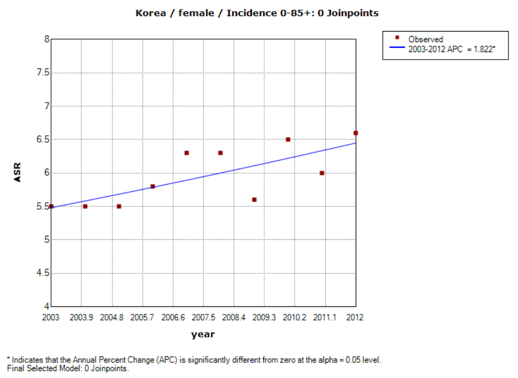

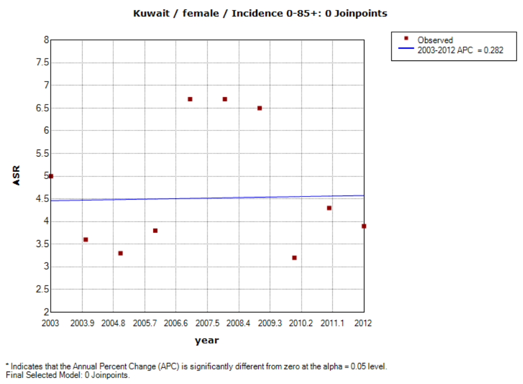

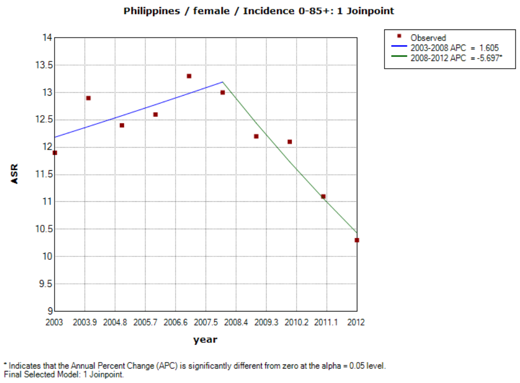

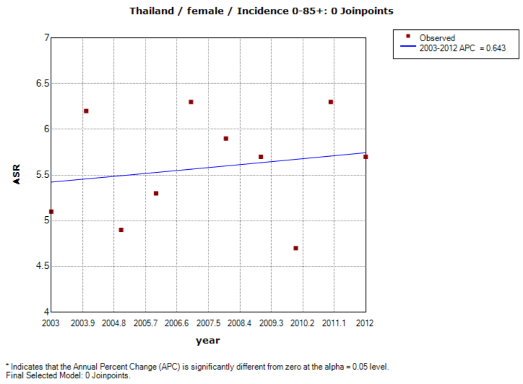

Oceania

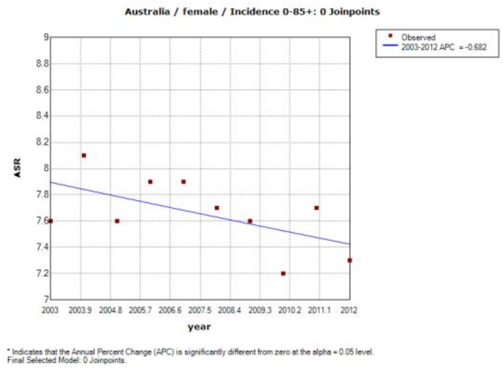

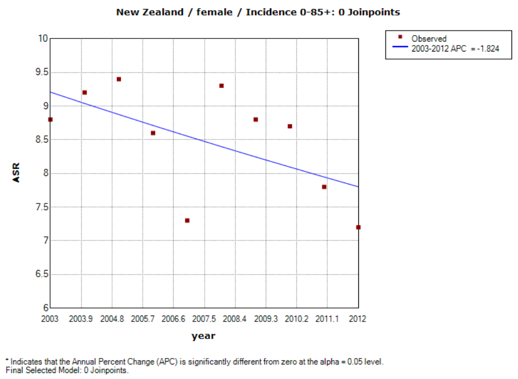

Northern America

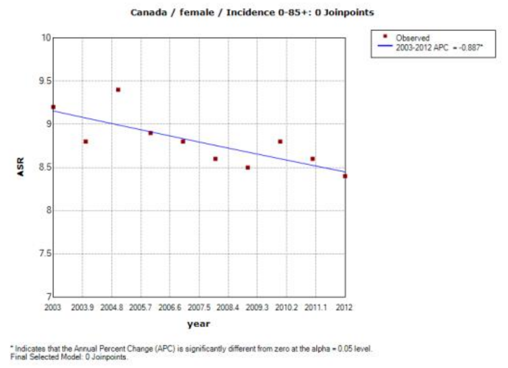

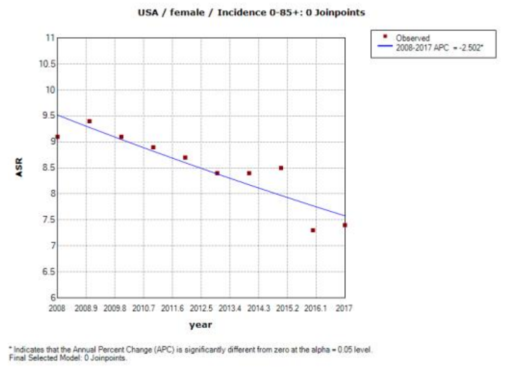

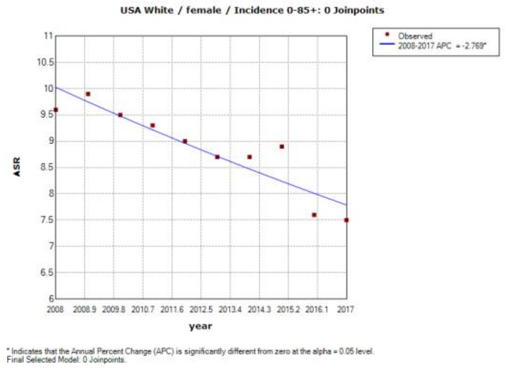

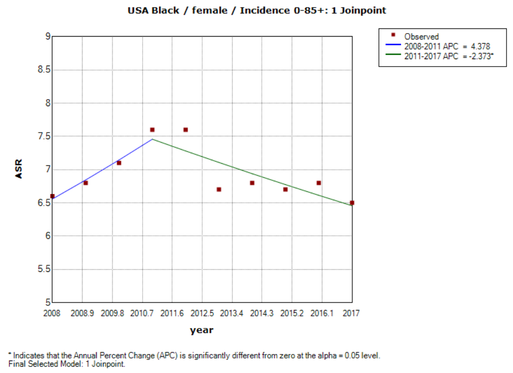

Southern America

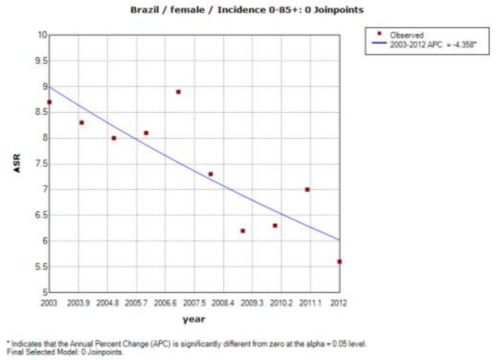

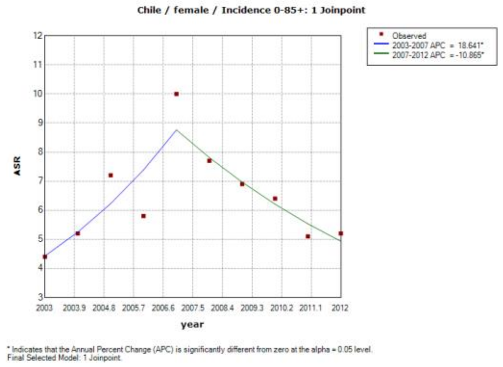

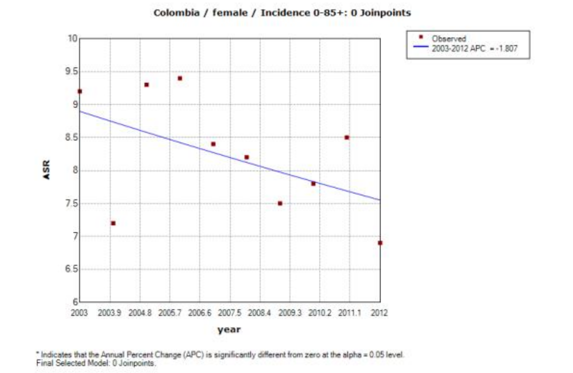

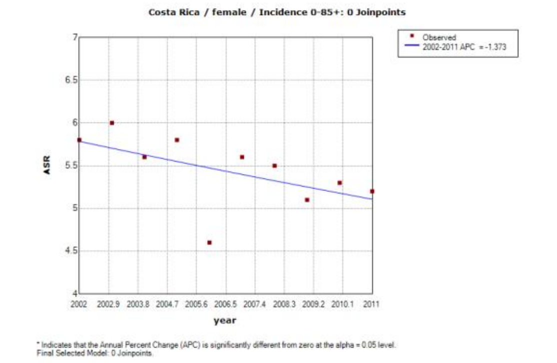

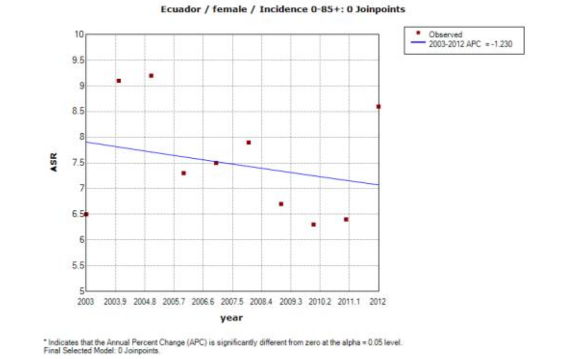

Northern Europe

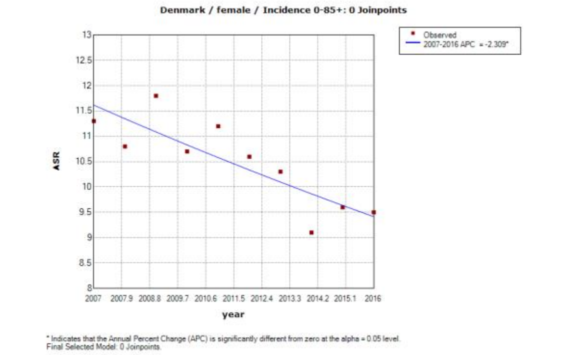

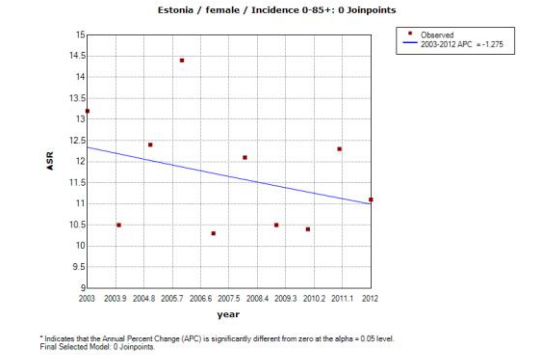

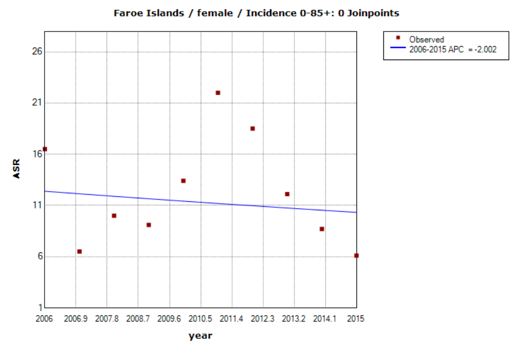

* Indicates that the Annual Percent Change (APC) is significantly different from zero at the alpha = 0.05 level.
Final Selected Model: 0 Joinpoints.

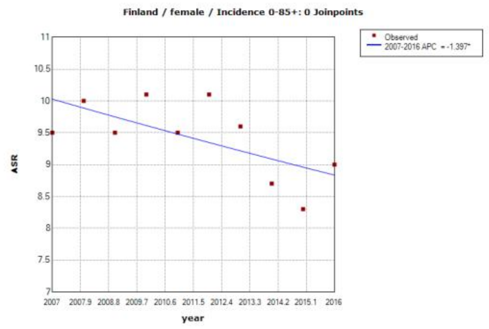

* Indicates that the Annual Percent Change (APC) is significantly different from zero at the alpha = 0.05 level.
Final Selected Model: 0 Joinpoints.

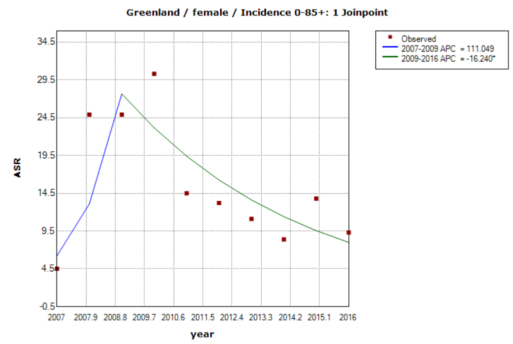

* Indicates that the Annual Percent Change (APC) is significantly different from zero at the alpha = 0.05 level.
Final Selected Model: 1 Joinpoint.

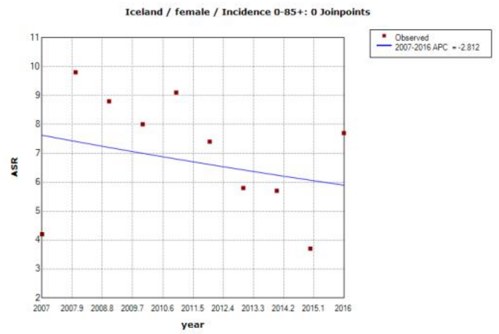

* Indicates that the Annual Percent Change (APC) is significantly different from zero at the alpha = 0.05 level.
Final Selected Model: 0 Joinpoints.

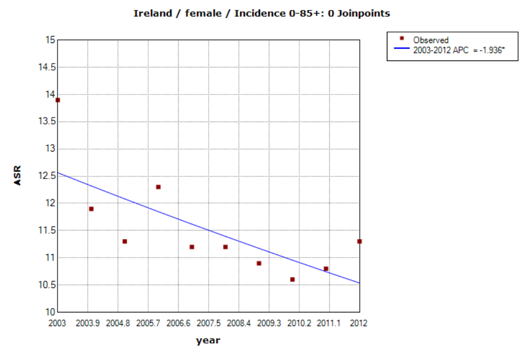

* Indicates that the Annual Percent Change (APC) is significantly different from zero at the alpha = 0.05 level.
Final Selected Model: 0 Joinpoints.

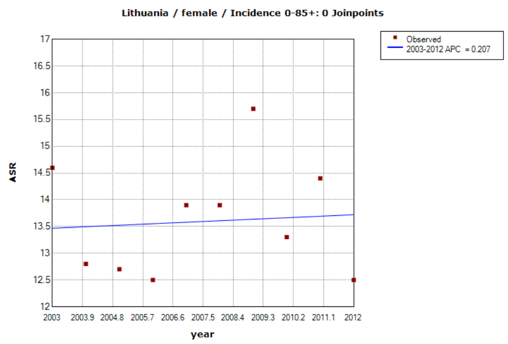

* Indicates that the Annual Percent Change (APC) is significantly different from zero at the alpha = 0.05 level.
Final Selected Model: 0 Joinpoints.

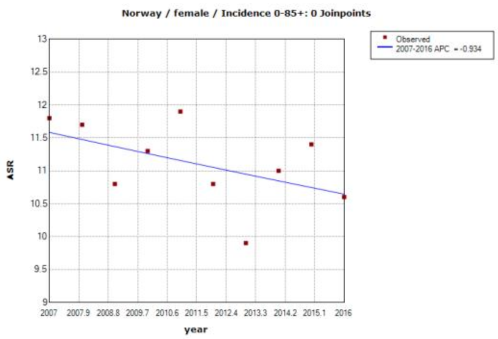

* Indicates that the Annual Percent Change (APC) is significantly different from zero at the alpha = 0.05 level.
Final Selected Model: 0 Joinpoints.

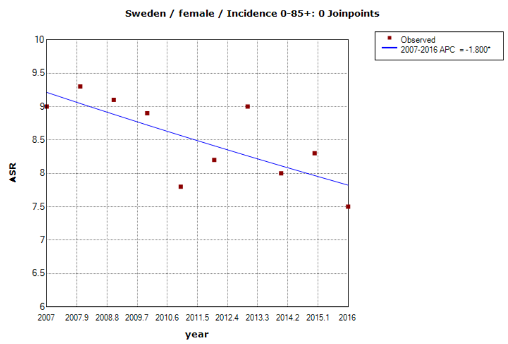

* Indicates that the Annual Percent Change (APC) is significantly different from zero at the alpha = 0.05 level.
Final Selected Model: 0 Joinpoints.

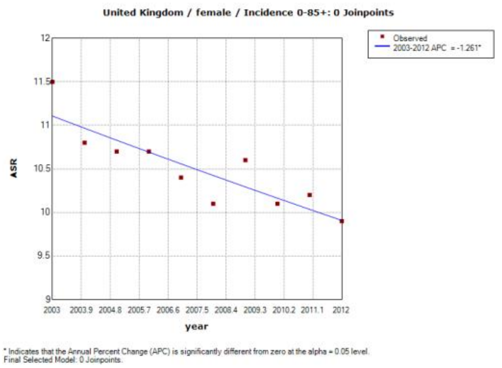

Western Europe

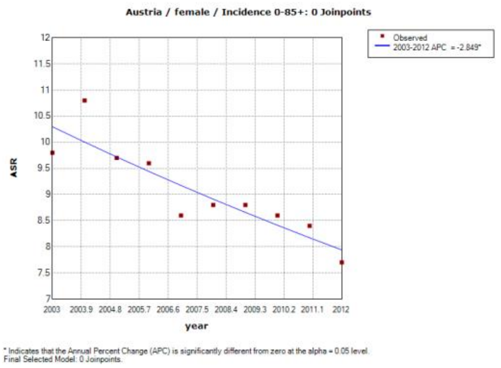

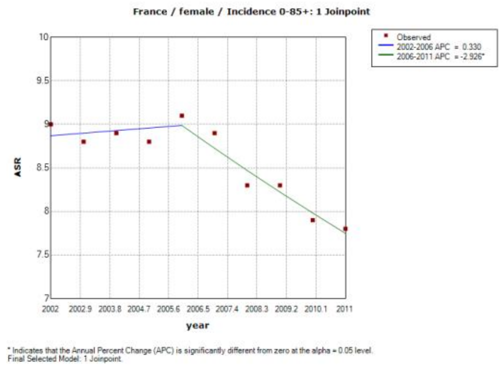

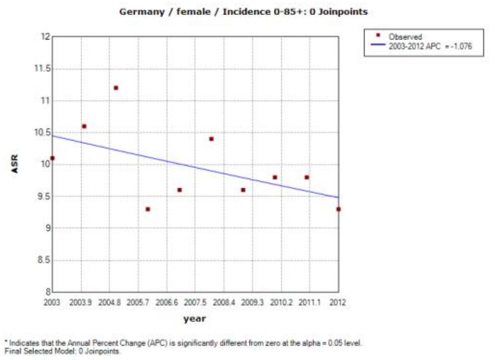

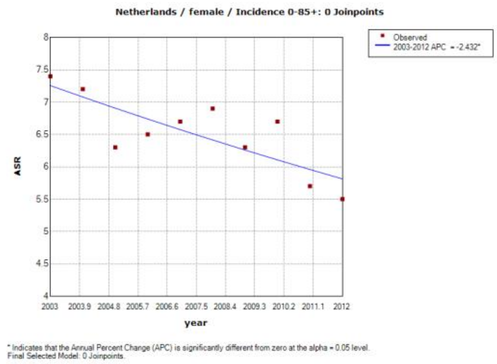

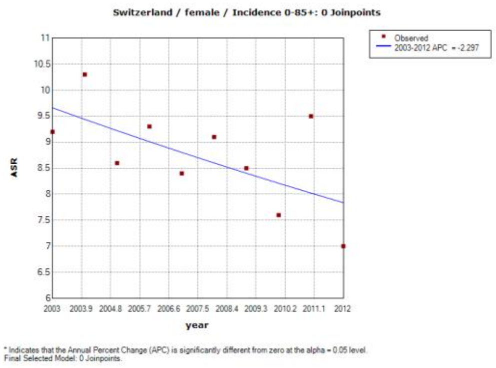

Southern Europe

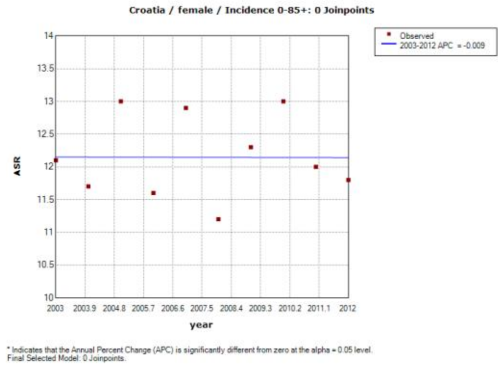

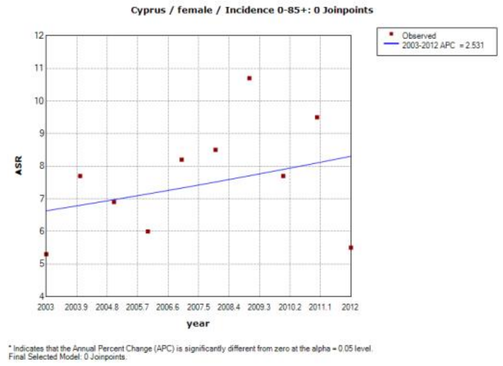

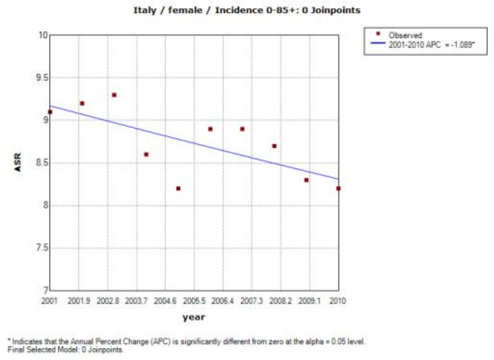

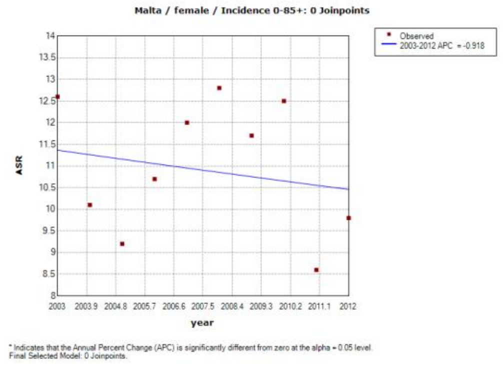

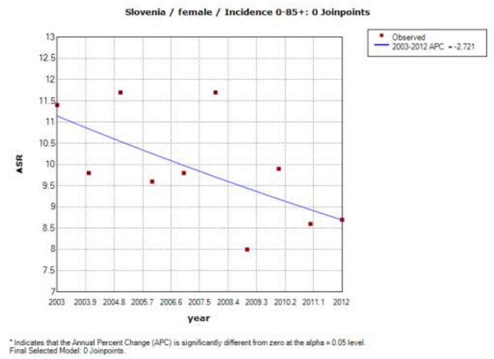

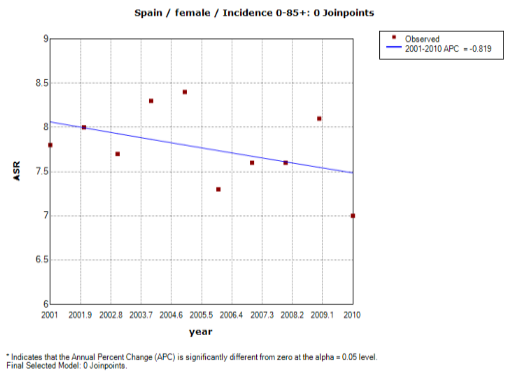

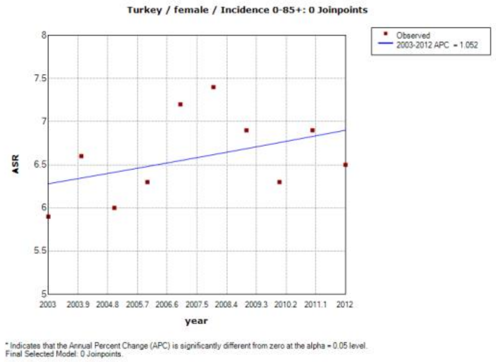

Eastern Europe

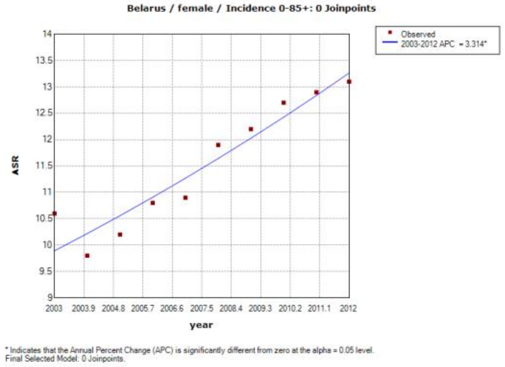

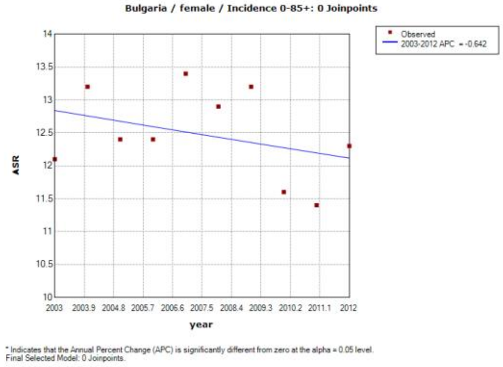

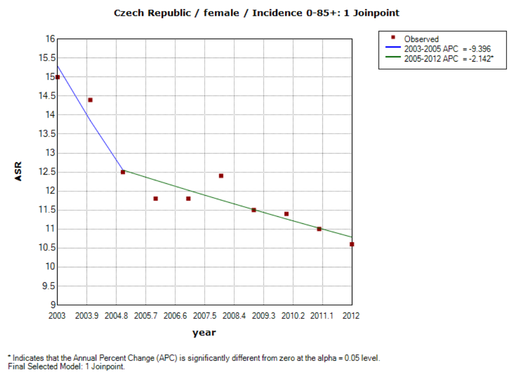
Czech Republic / female / Incidence 0-85+: 1 Joinpoint
Observed
2003-2005 APC = -9.396
2005-2012 APC = -2.142*
ASR
year
* Indicates that the Annual Percent Change (APC) is significantly different from zero at the alpha = 0.05 level.
Final Selected Model: 1 Joinpoint.

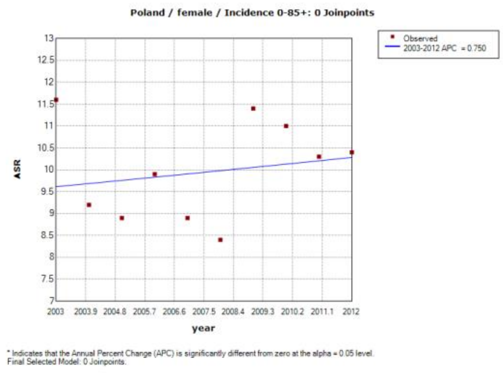
Poland / female / Incidence 0-85+: 0 Joinpoints
Observed
2003-2012 APC = 0.750
ASR
year
* Indicates that the Annual Percent Change (APC) is significantly different from zero at the alpha = 0.05 level.
Final Selected Model: 0 Joinpoints.

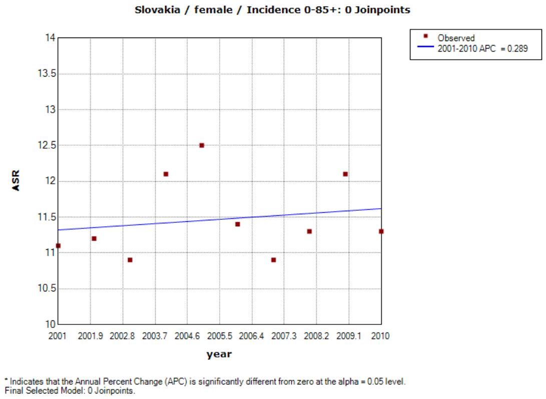
Slovakia / female / Incidence 0-85+: 0 Joinpoints
Observed
2001-2010 APC = 0.289
ASR
year
* Indicates that the Annual Percent Change (APC) is significantly different from zero at the alpha = 0.05 level.
Final Selected Model: 0 Joinpoints.

Africa

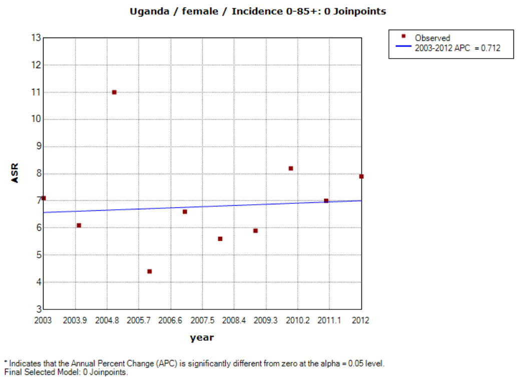
Uganda / female / Incidence 0-85+: 0 Joinpoints
Observed
2003-2012 APC = 0.712
ASR
year
* Indicates that the Annual Percent Change (APC) is significantly different from zero at the alpha = 0.05 level.
Final Selected Model: 0 Joinpoints.

b.) Incidence female below 40 years old

Asia

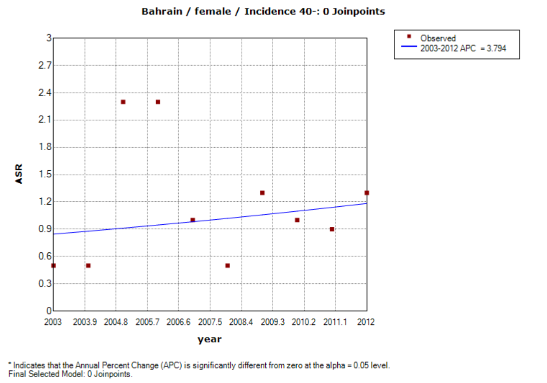

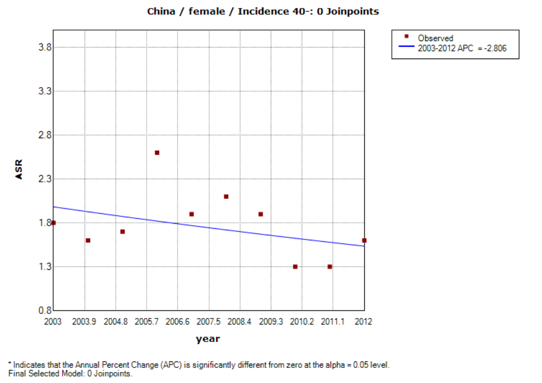

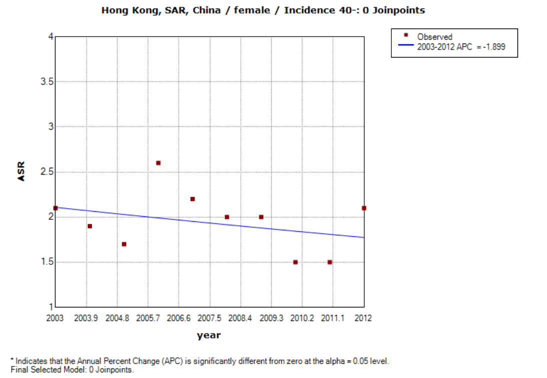

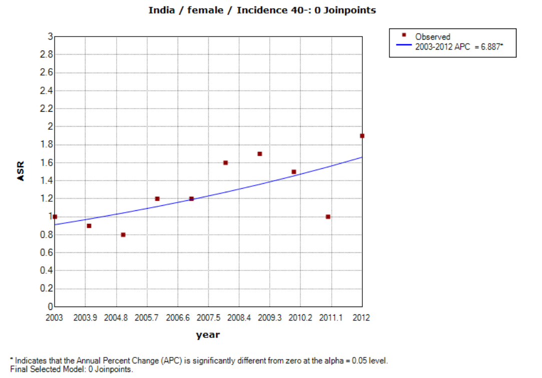

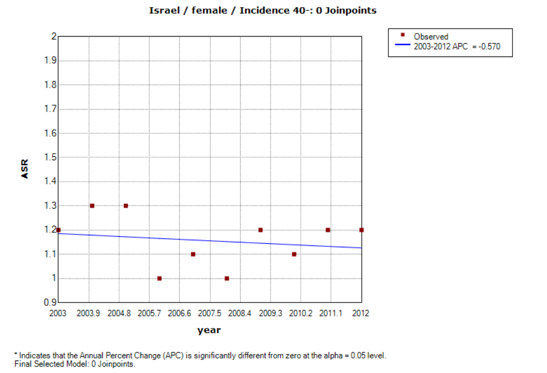

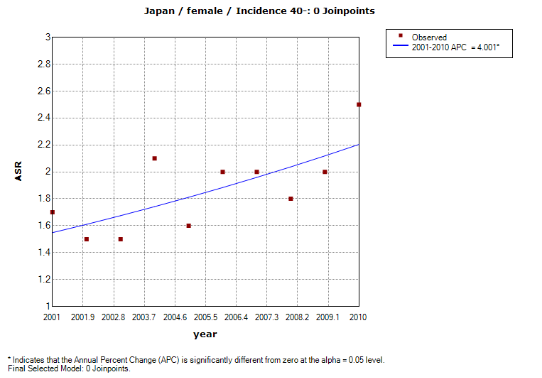

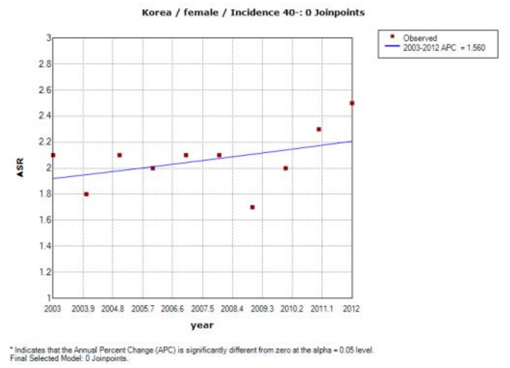

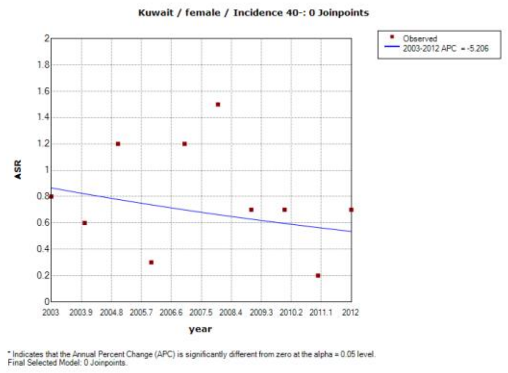

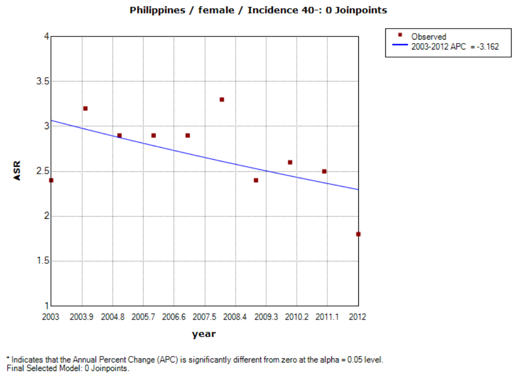

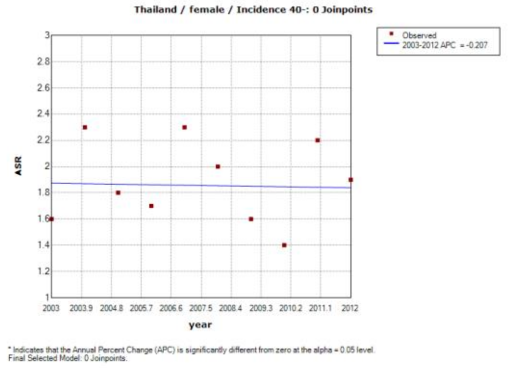

Oceania

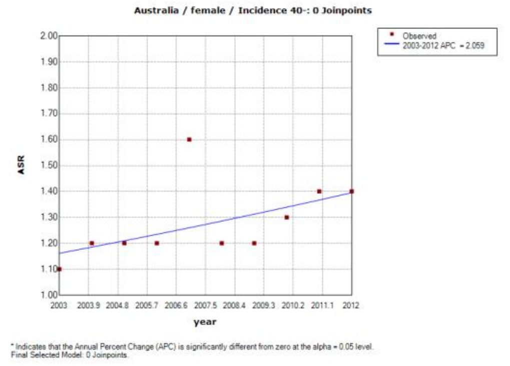

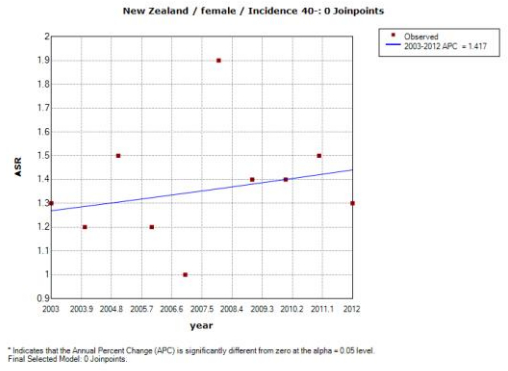

Northern America

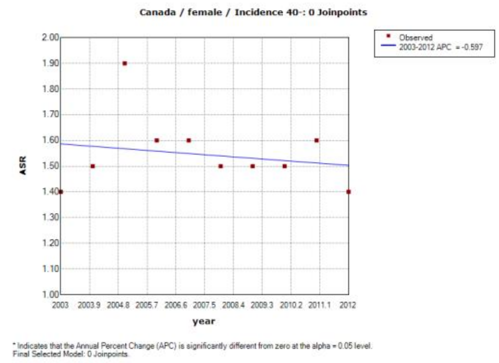

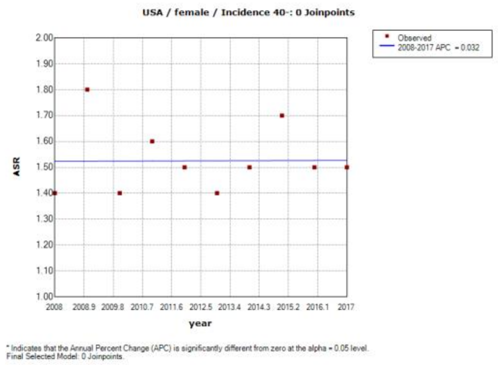

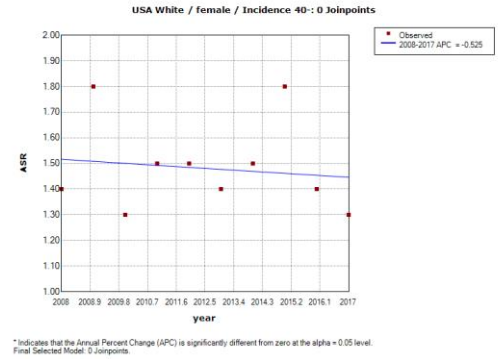

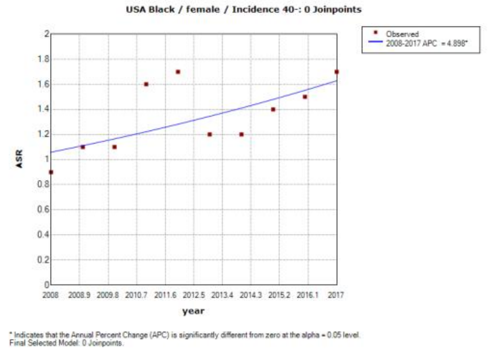

Southern America

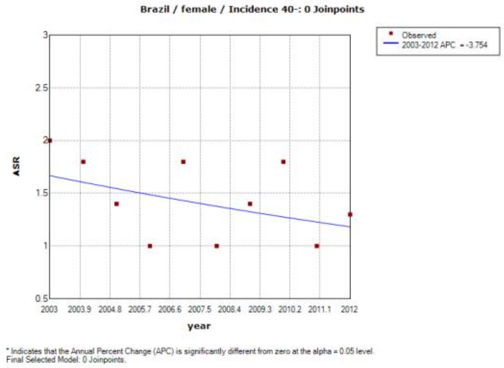

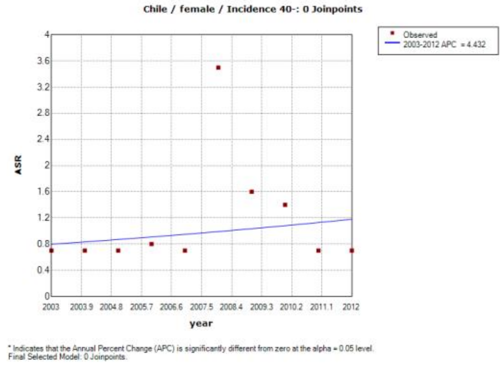

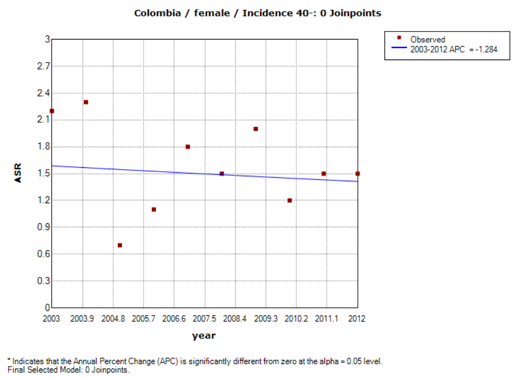

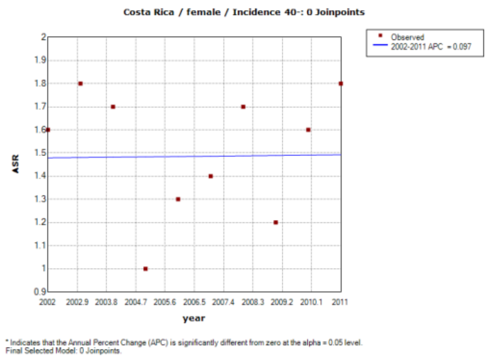

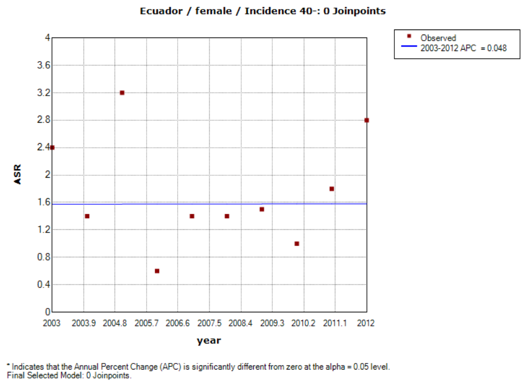

Northern Europe

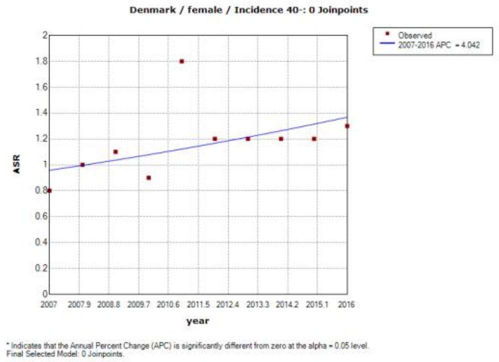

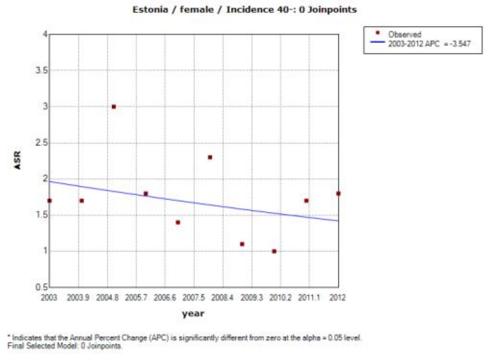

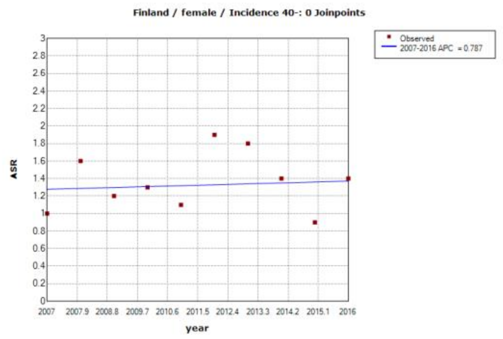

* Indicates that the Annual Percent Change (APC) is significantly different from zero at the alpha = 0.05 level.
Final Selected Model: 0 Joinpoints.

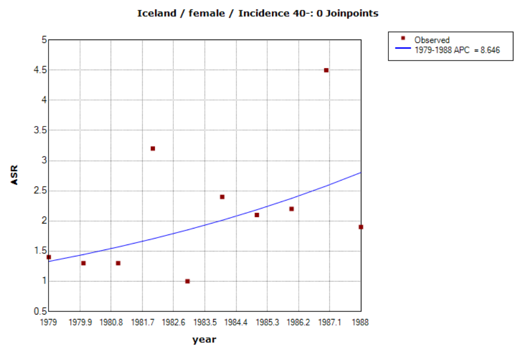

* Indicates that the Annual Percent Change (APC) is significantly different from zero at the alpha = 0.05 level.
Final Selected Model: 0 Joinpoints.

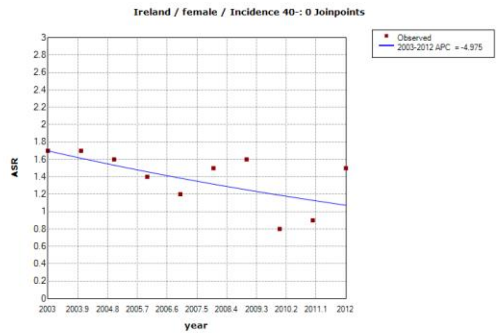

* Indicates that the Annual Percent Change (APC) is significantly different from zero at the alpha = 0.05 level.
Final Selected Model: 0 Joinpoints.

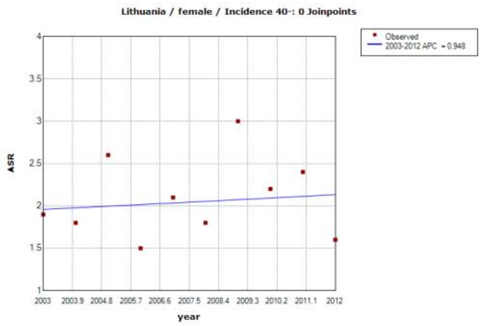

* Indicates that the Annual Percent Change (APC) is significantly different from zero at the alpha = 0.05 level.
Final Selected Model: 0 Joinpoints.

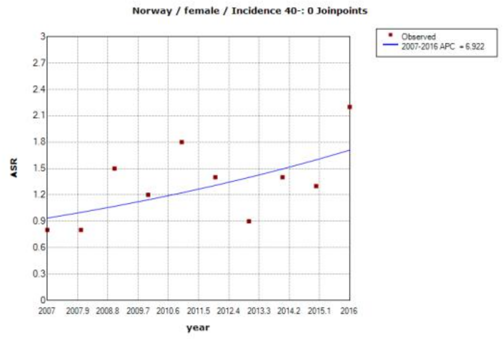

* Indicates that the Annual Percent Change (APC) is significantly different from zero at the alpha = 0.05 level.
Final Selected Model: 0 Joinpoints.

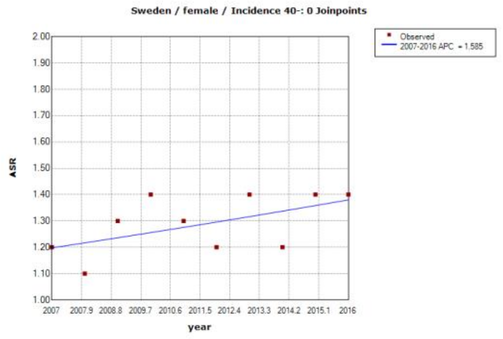

* Indicates that the Annual Percent Change (APC) is significantly different from zero at the alpha = 0.05 level.
Final Selected Model: 0 Joinpoints.

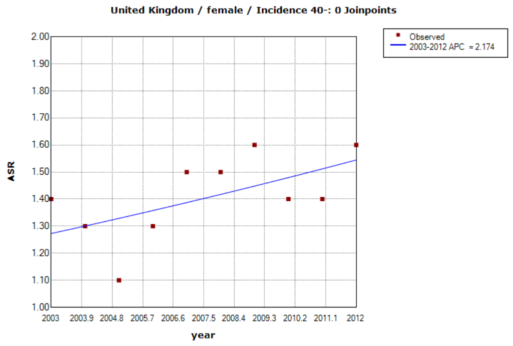

* Indicates that the Annual Percent Change (APC) is significantly different from zero at the alpha = 0.05 level.
Final Selected Model: 0 Joinpoints.

Western Europe

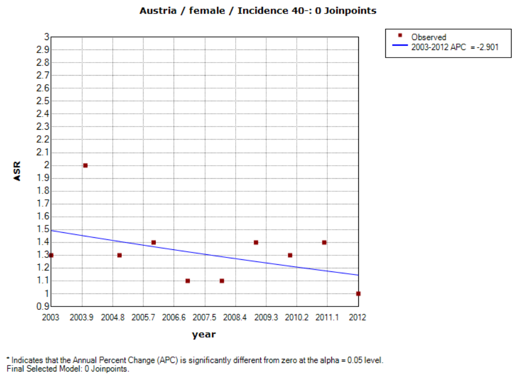

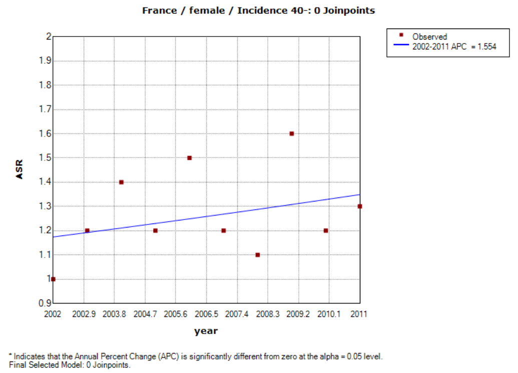

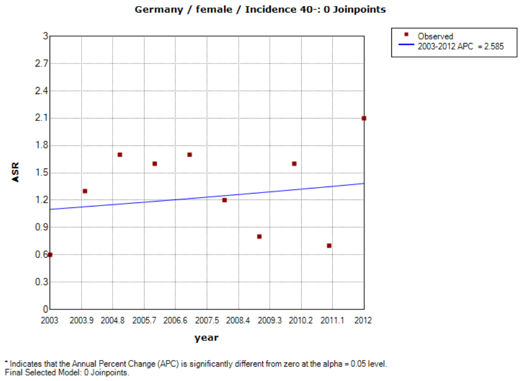

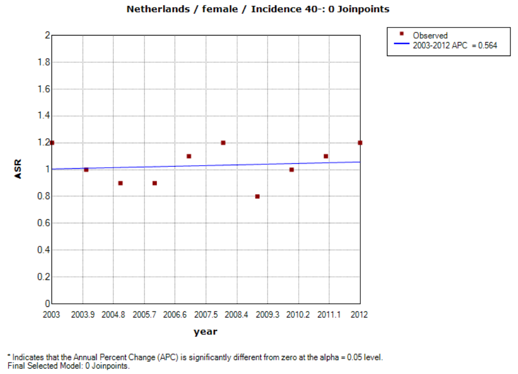

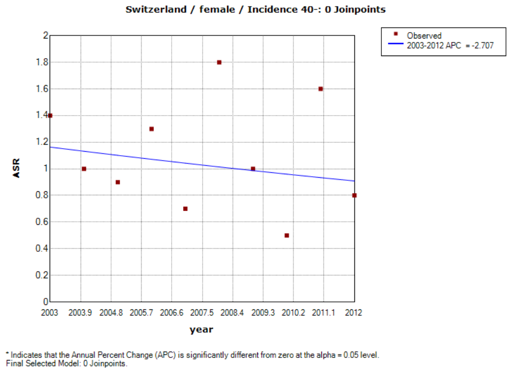

Southern Europe

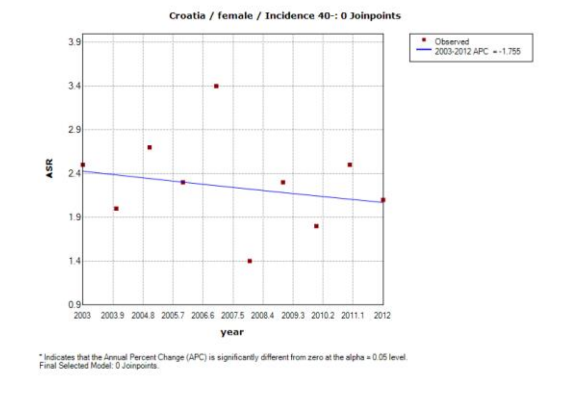

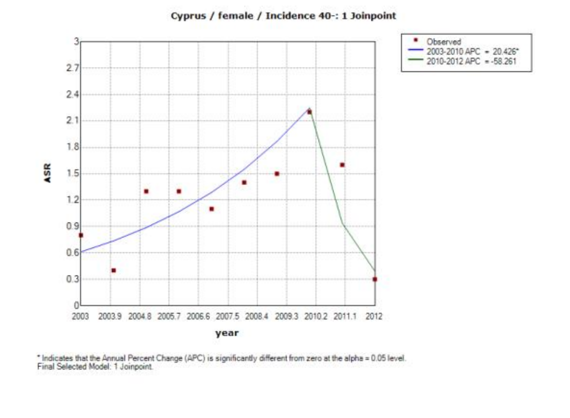

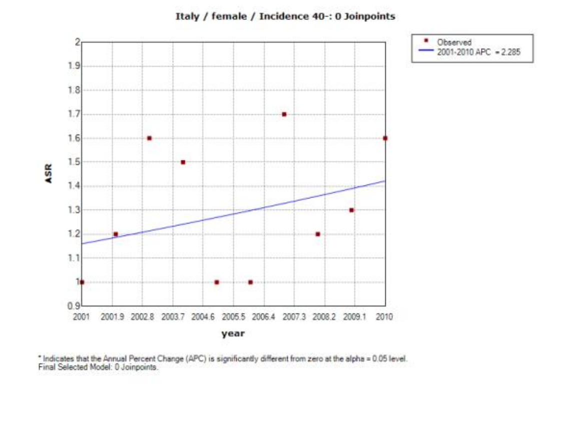

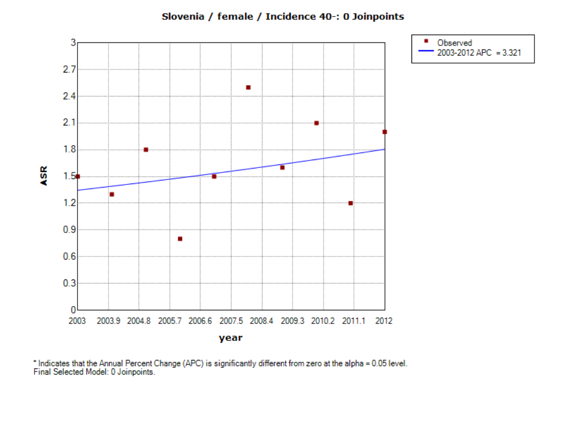

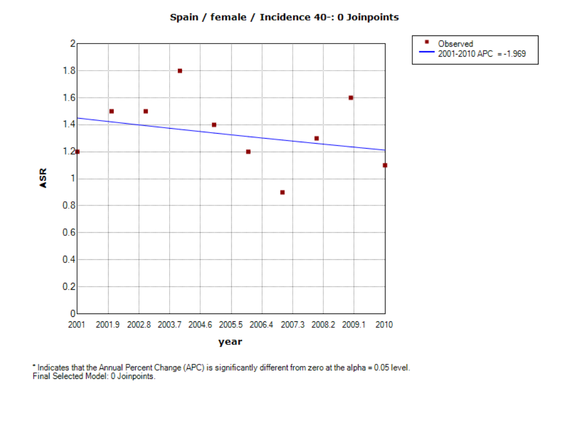

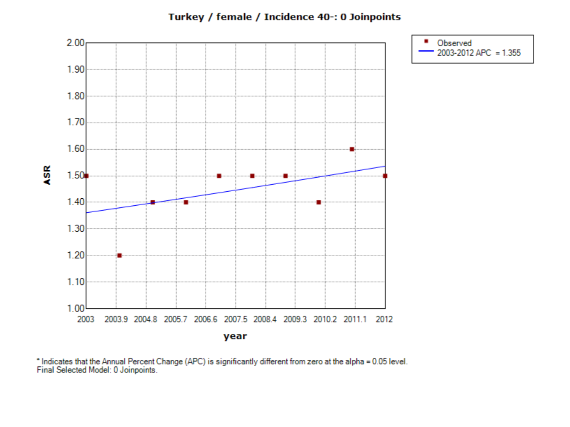

Eastern Europe

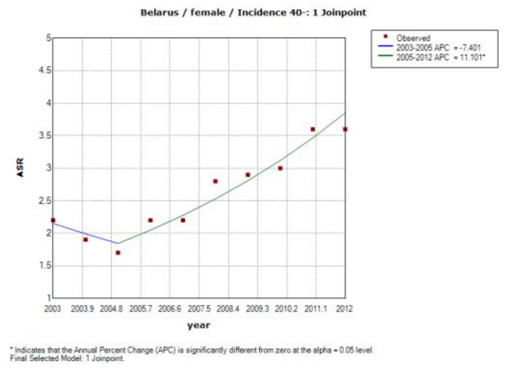
Belarus / female / Incidence 40-: 1 Joinpoint
Observed
2003-2005 APC = -7.401
2005-2012 APC = 11.101*
ASR
year
* Indicates that the Annual Percent Change (APC) is significantly different from zero at the alpha = 0.05 level.
Final Selected Model: 1 Joinpoint.

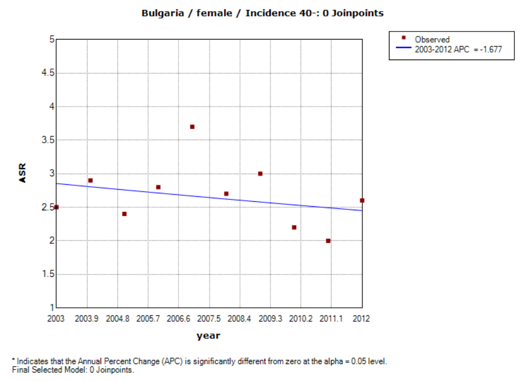
Bulgaria / female / Incidence 40-: 0 Joinpoints
Observed
2003-2012 APC = -1.677
ASR
year
* Indicates that the Annual Percent Change (APC) is significantly different from zero at the alpha = 0.05 level.
Final Selected Model: 0 Joinpoints.

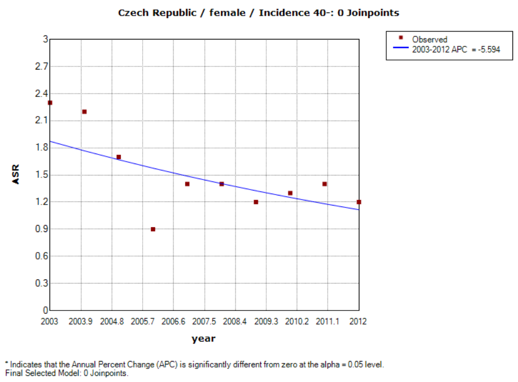
Czech Republic / female / Incidence 40-: 0 Joinpoints
Observed
2003-2012 APC = -5.594
ASR
year
* Indicates that the Annual Percent Change (APC) is significantly different from zero at the alpha = 0.05 level.
Final Selected Model: 0 Joinpoints.

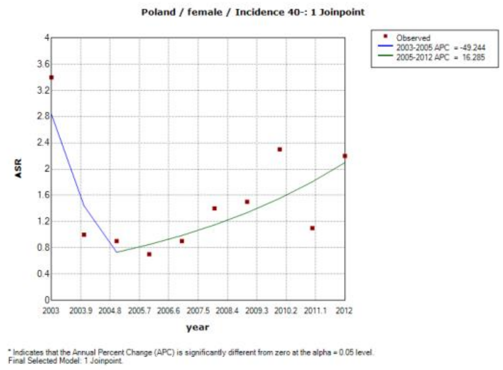
Poland / female / Incidence 40-: 1 Joinpoint
Observed
2003-2005 APC = -49.244
2005-2012 APC = 16.285
ASR
year
* Indicates that the Annual Percent Change (APC) is significantly different from zero at the alpha = 0.05 level.
Final Selected Model: 1 Joinpoint.

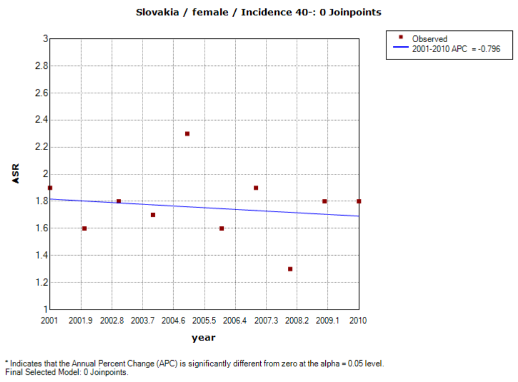
Slovakia / female / Incidence 40-: 0 Joinpoints
Observed
2001-2010 APC = -0.796
ASR
year
* Indicates that the Annual Percent Change (APC) is significantly different from zero at the alpha = 0.05 level.
Final Selected Model: 0 Joinpoints.

Africa

Uganda / female / Incidence 40-: 0 Joinpoints

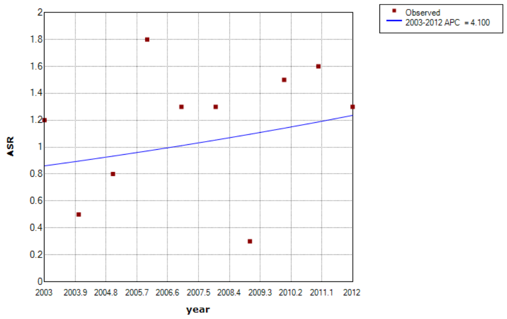

* Indicates that the Annual Percent Change (APC) is significantly different from zero at the alpha = 0.05 level.
Final Selected Model: 0 Joinpoints.

c.) Incidence female below 50 years old

Asia

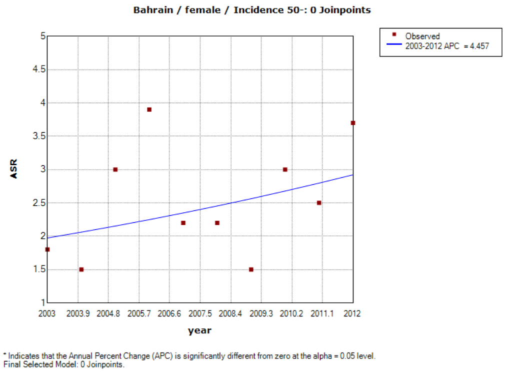

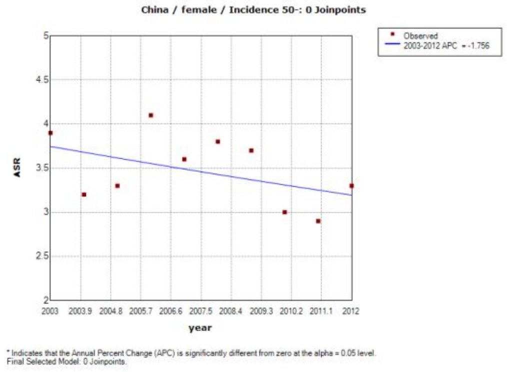

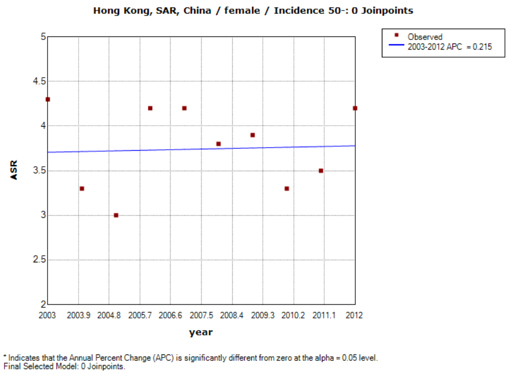

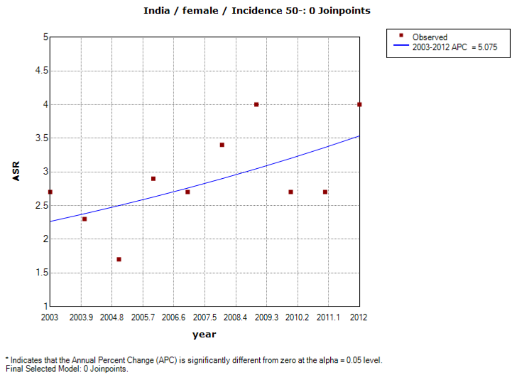

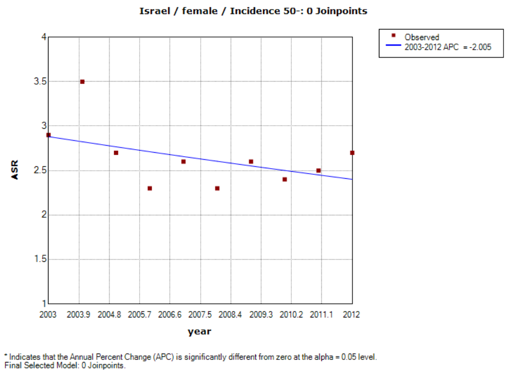

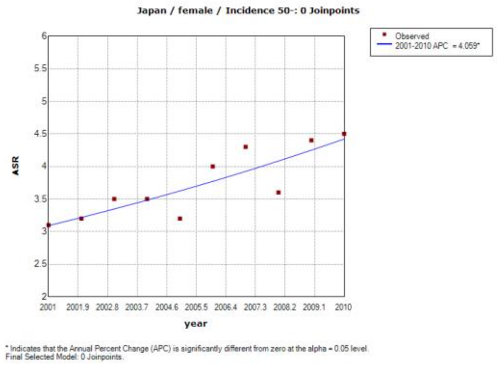

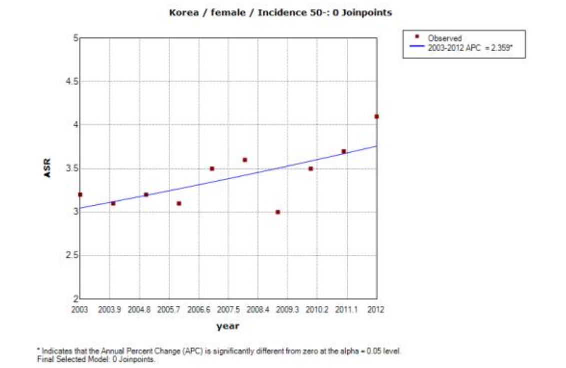

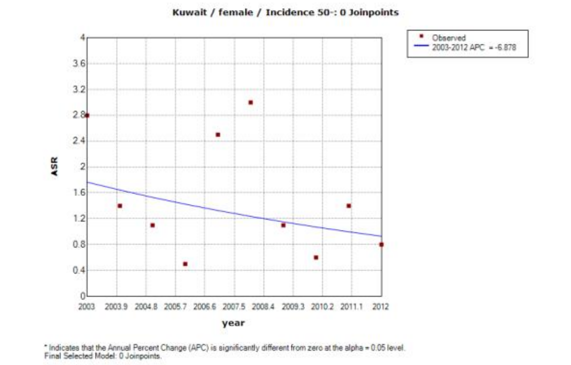

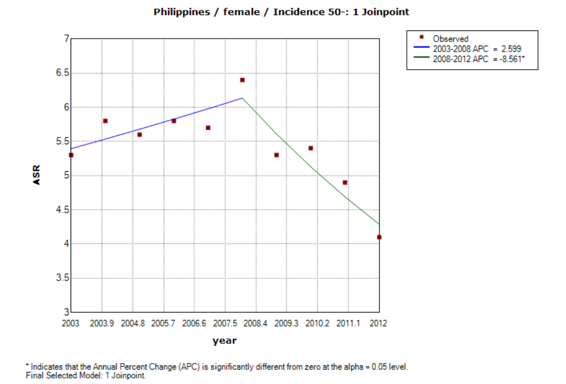

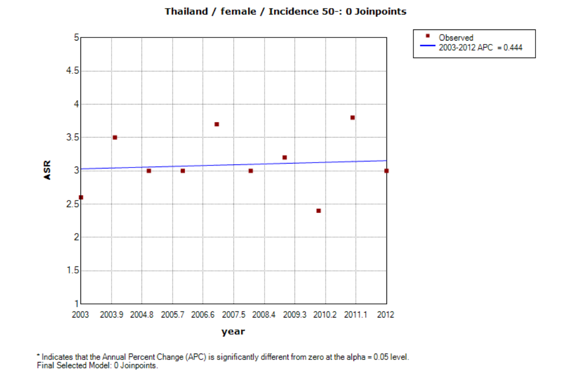

Oceania

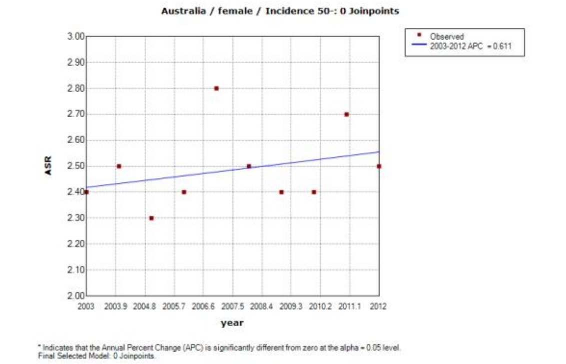

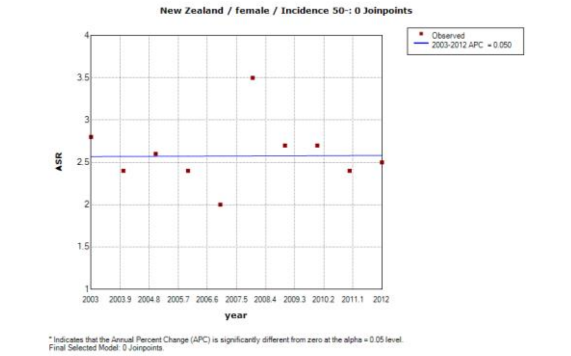

Northern America

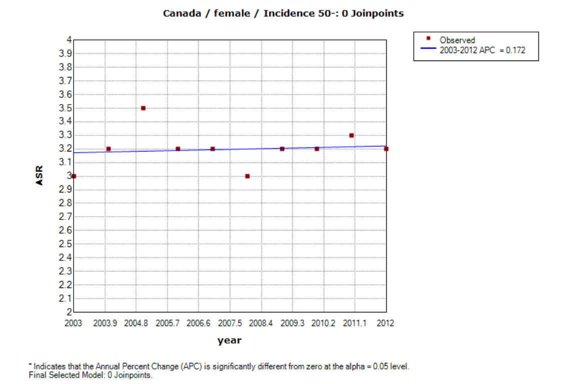

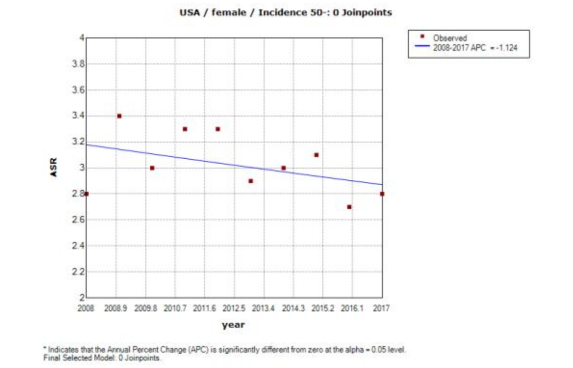

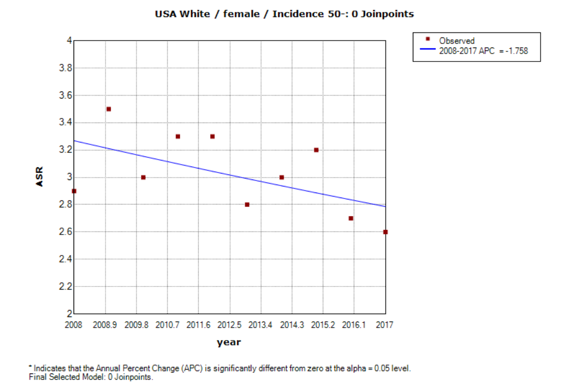

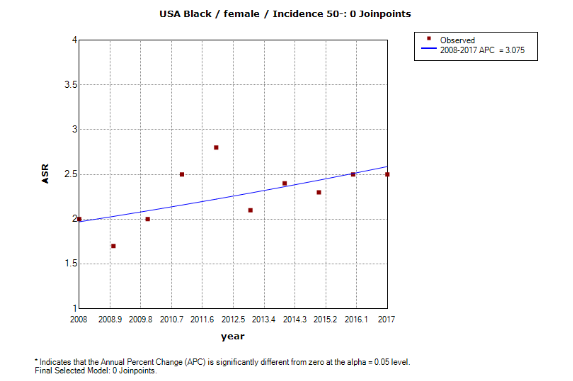

Southern America

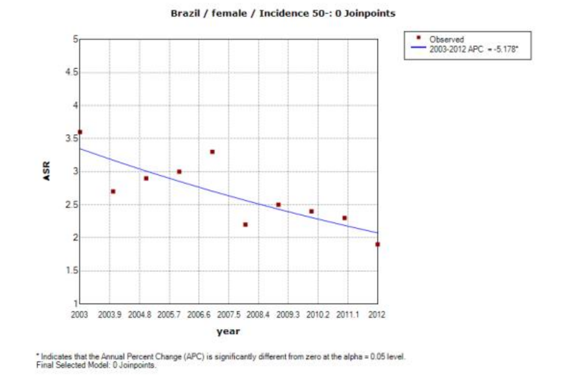

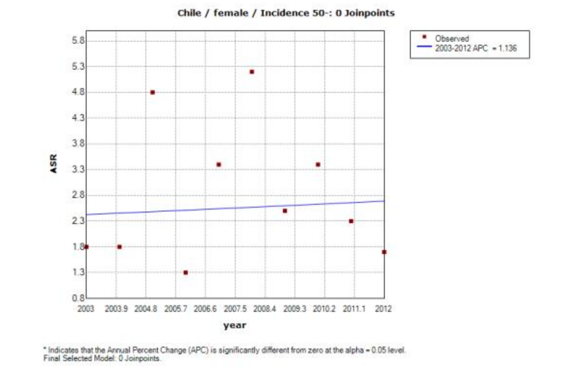

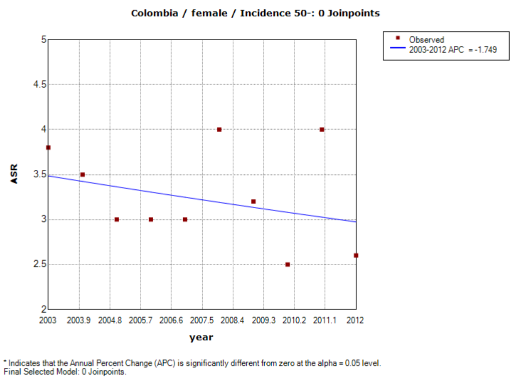

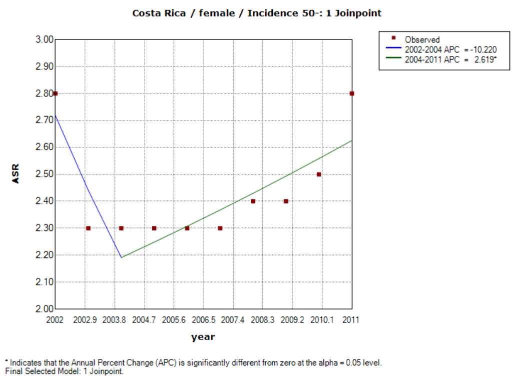

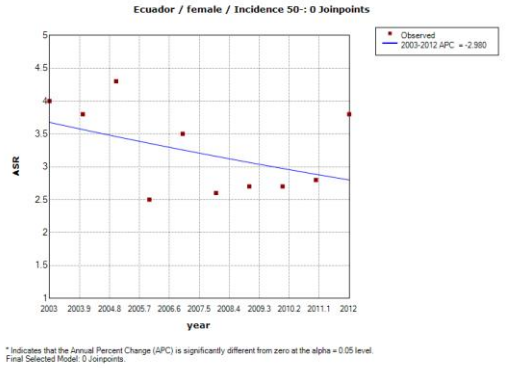

Northern Europe

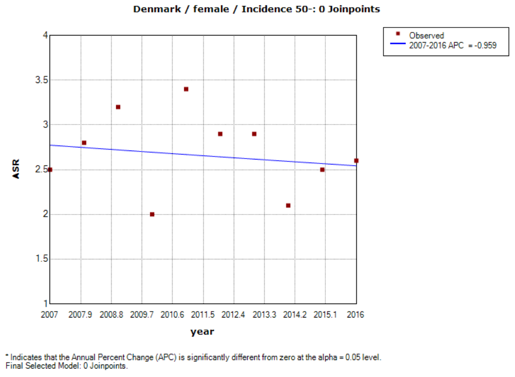

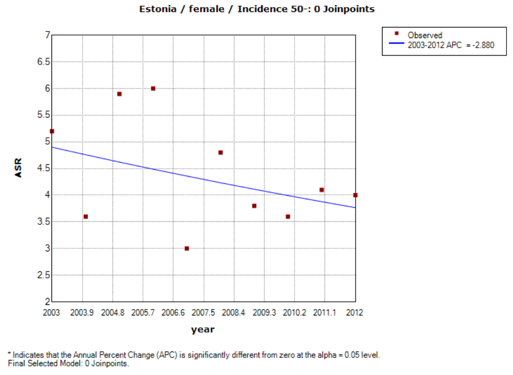

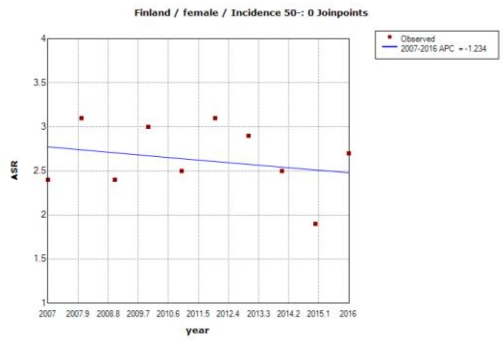

* Indicates that the Annual Percent Change (APC) is significantly different from zero at the alpha = 0.05 level.
Final Selected Model: 0 Joinpoints.

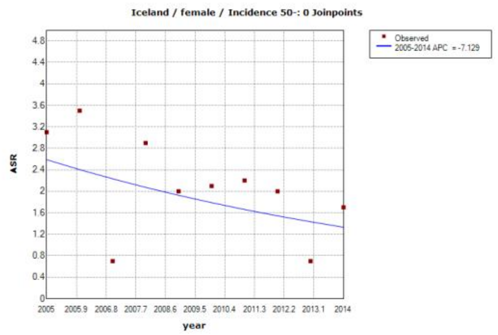

* Indicates that the Annual Percent Change (APC) is significantly different from zero at the alpha = 0.05 level.
Final Selected Model: 0 Joinpoints.

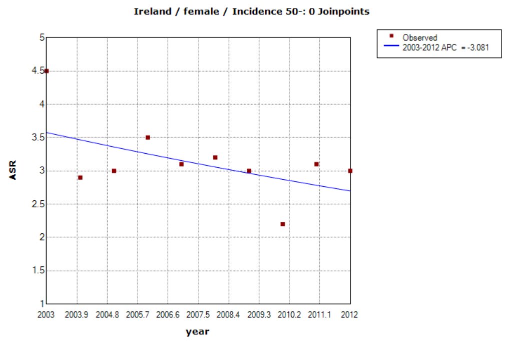

* Indicates that the Annual Percent Change (APC) is significantly different from zero at the alpha = 0.05 level.
Final Selected Model: 0 Joinpoints.

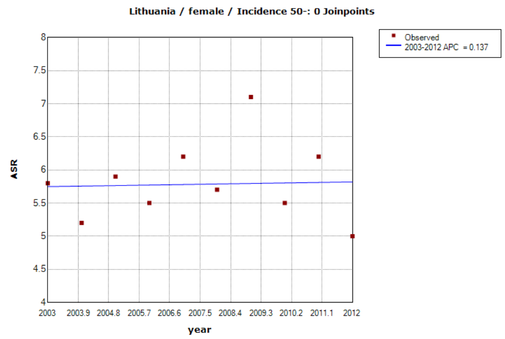

* Indicates that the Annual Percent Change (APC) is significantly different from zero at the alpha = 0.05 level.
Final Selected Model: 0 Joinpoints.

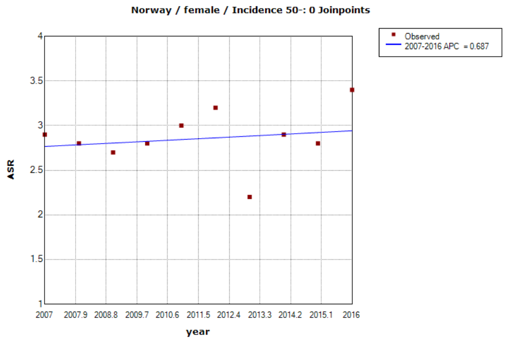

* Indicates that the Annual Percent Change (APC) is significantly different from zero at the alpha = 0.05 level.
Final Selected Model: 0 Joinpoints.

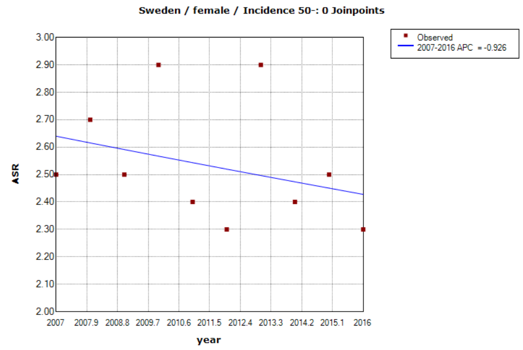

* Indicates that the Annual Percent Change (APC) is significantly different from zero at the alpha = 0.05 level.
Final Selected Model: 0 Joinpoints.

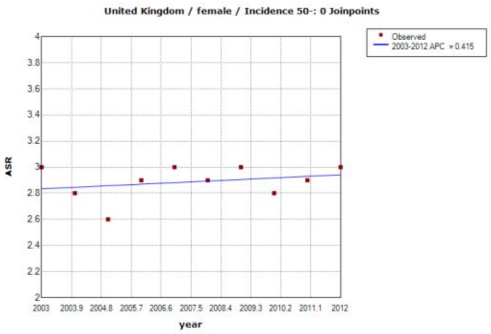

* Indicates that the Annual Percent Change (APC) is significantly different from zero at the alpha = 0.05 level.
Final Selected Model: 0 Joinpoints.

Western Europe

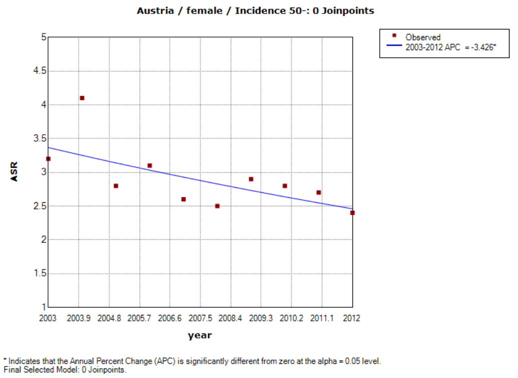

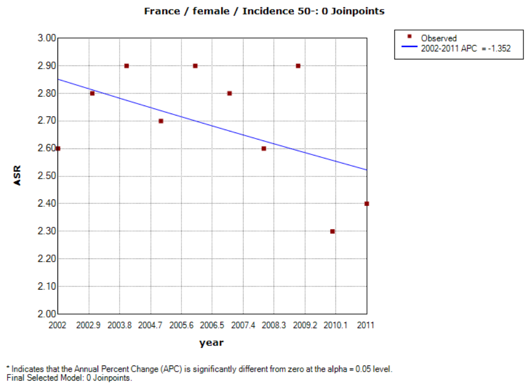

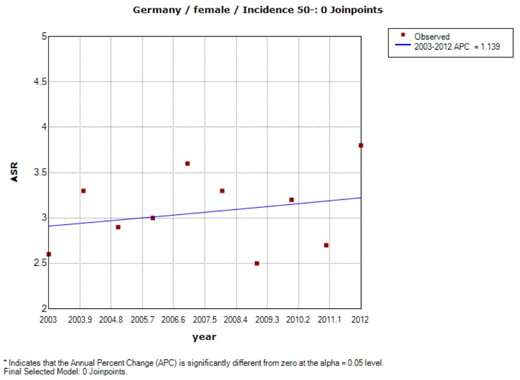

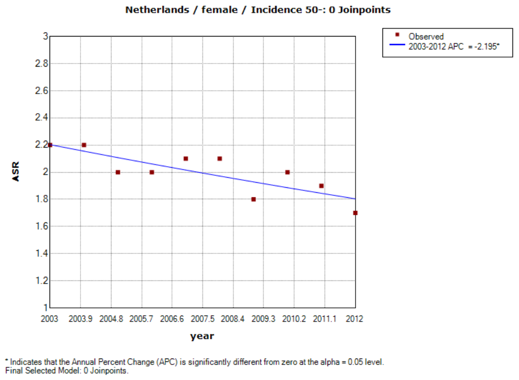

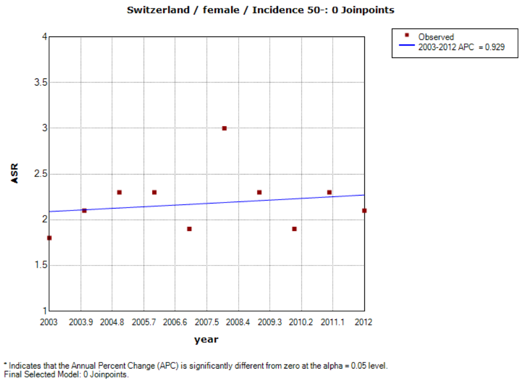

Southern Europe

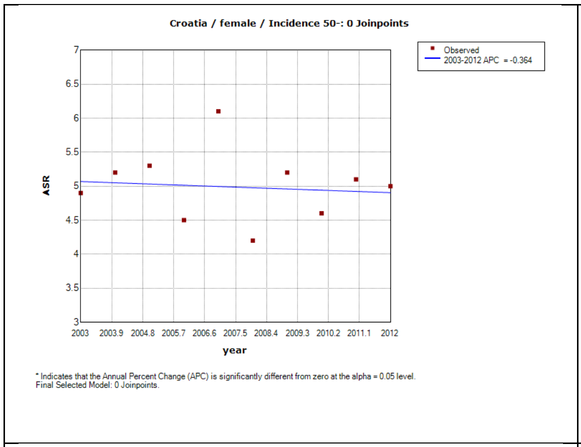
Croatia / female / Incidence 50-: 0 Joinpoints
Observed
2003-2012 APC = -0.364
ASR
year
* Indicates that the Annual Percent Change (APC) is significantly different from zero at the alpha = 0.05 level.
Final Selected Model: 0 Joinpoints.

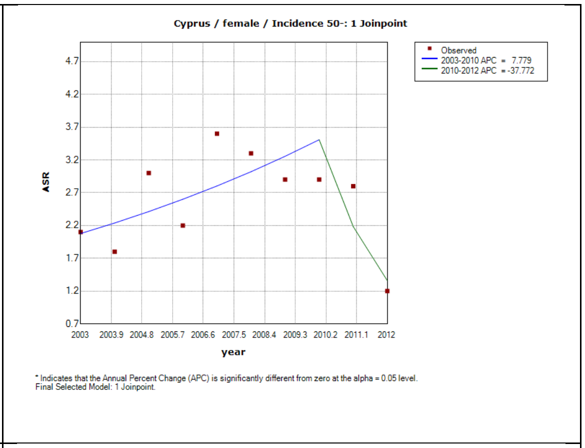
Cyprus / female / Incidence 50-: 1 Joinpoint
Observed
2003-2010 APC = 7.779
2010-2012 APC = -37.772
ASR
year
* Indicates that the Annual Percent Change (APC) is significantly different from zero at the alpha = 0.05 level.
Final Selected Model: 1 Joinpoint.

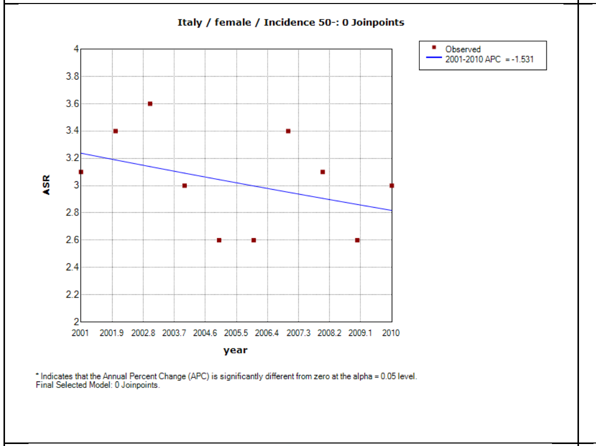
Italy / female / Incidence 50-: 0 Joinpoints
Observed
2001-2010 APC = -1.531
ASR
year
* Indicates that the Annual Percent Change (APC) is significantly different from zero at the alpha = 0.05 level.
Final Selected Model: 0 Joinpoints.

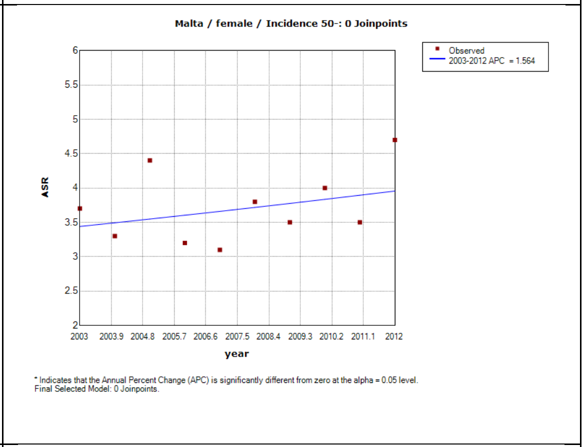
Malta / female / Incidence 50-: 0 Joinpoints
Observed
2003-2012 APC = 1.564
ASR
year
* Indicates that the Annual Percent Change (APC) is significantly different from zero at the alpha = 0.05 level.
Final Selected Model: 0 Joinpoints.

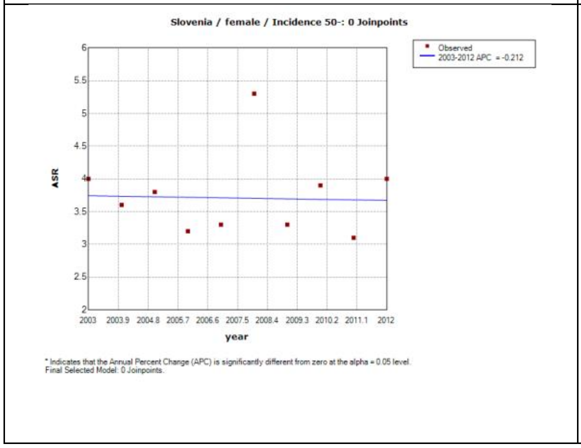
Slovenia / female / Incidence 50-: 0 Joinpoints
Observed
2003-2012 APC = -0.212
ASR
year
* Indicates that the Annual Percent Change (APC) is significantly different from zero at the alpha = 0.05 level.
Final Selected Model: 0 Joinpoints.

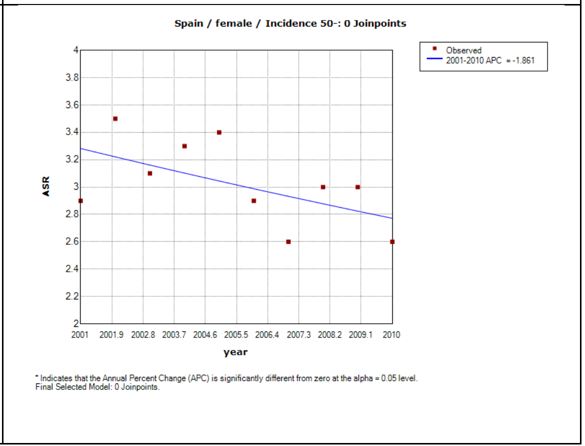
Spain / female / Incidence 50-: 0 Joinpoints
Observed
2001-2010 APC = -1.861
ASR
year
* Indicates that the Annual Percent Change (APC) is significantly different from zero at the alpha = 0.05 level.
Final Selected Model: 0 Joinpoints.

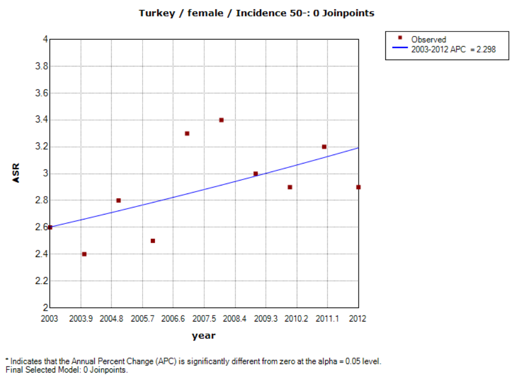

Eastern Europe

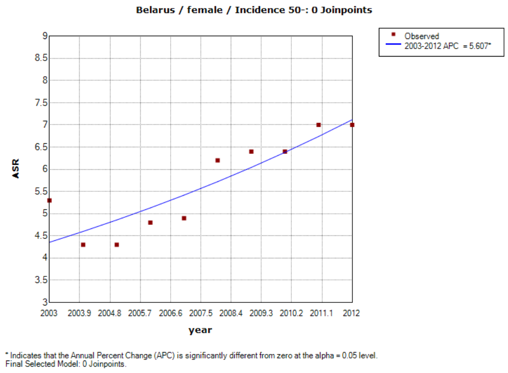

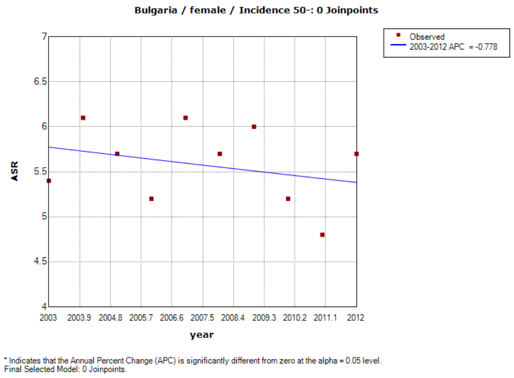

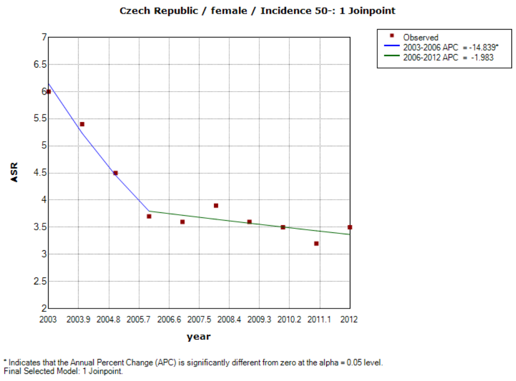

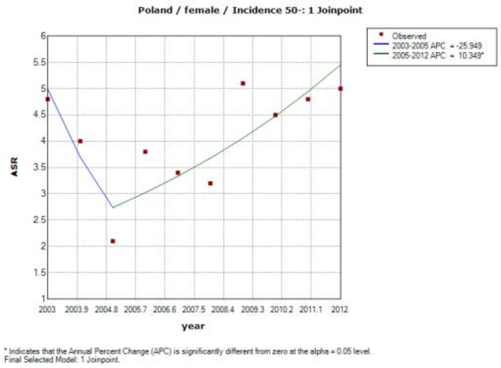

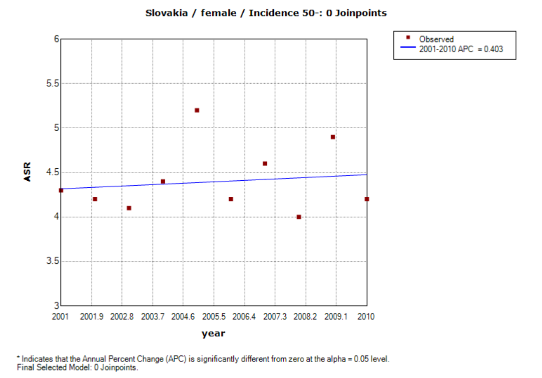
Slovakia / female / Incidence 50-: 0 Joinpoints
Observed
2001-2010 APC = 0.403
ASR
year
* Indicates that the Annual Percent Change (APC) is significantly different from zero at the alpha = 0.05 level.
Final Selected Model: 0 Joinpoints.

Africa

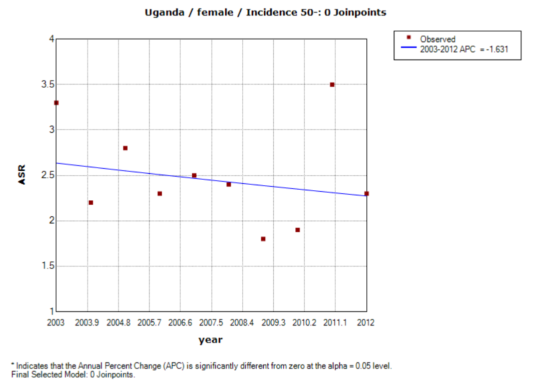
Uganda / female / Incidence 50-: 0 Joinpoints
Observed
2003-2012 APC = -1.631
ASR
year
* Indicates that the Annual Percent Change (APC) is significantly different from zero at the alpha = 0.05 level.
Final Selected Model: 0 Joinpoints.

d.) Incidence female above 50 years old

Asia

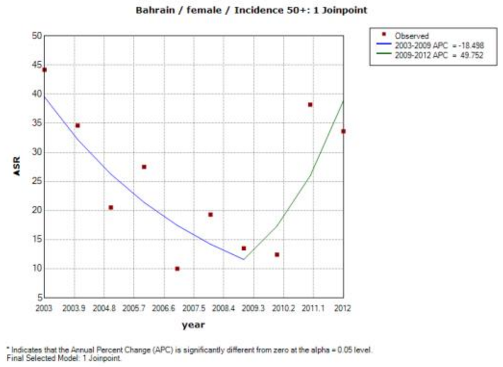

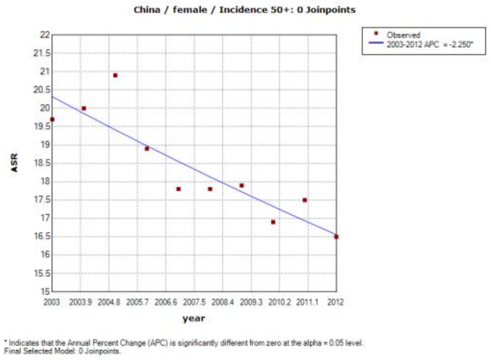

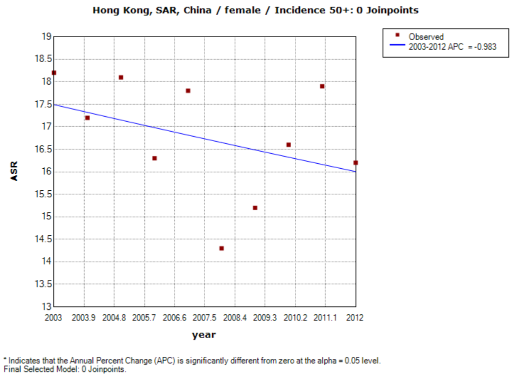

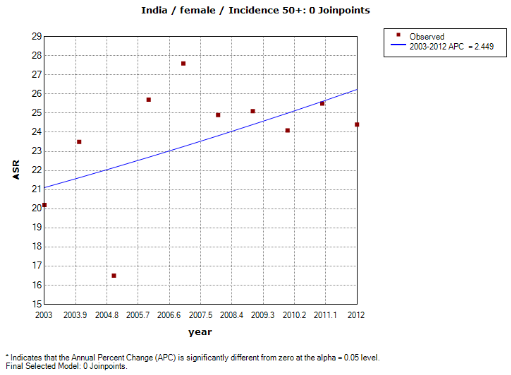

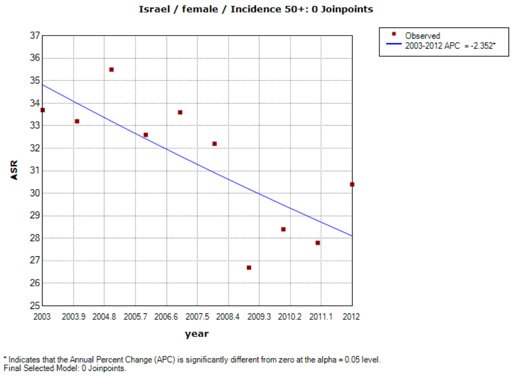

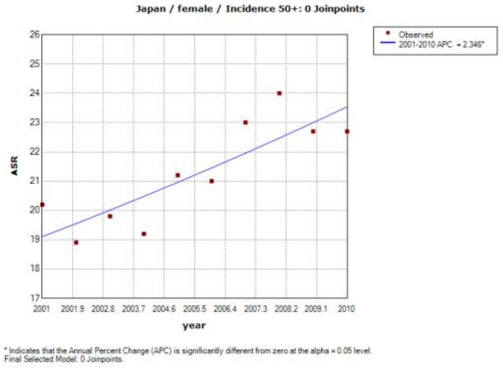

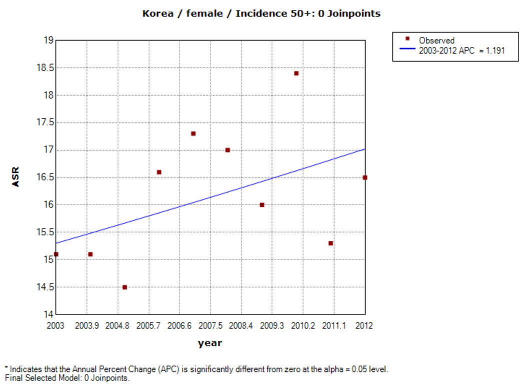

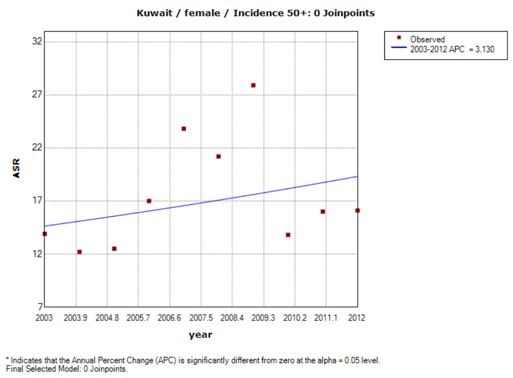

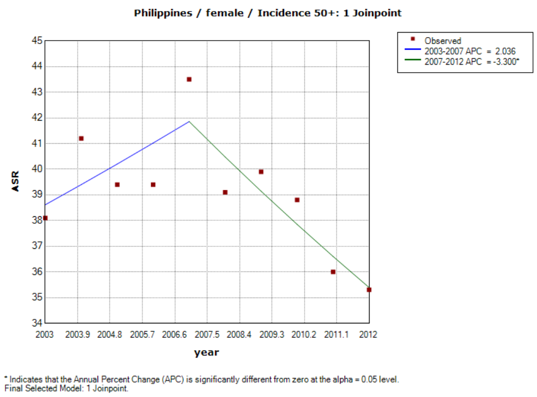

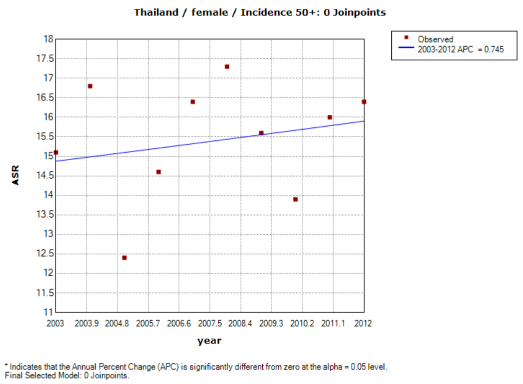

Oceania

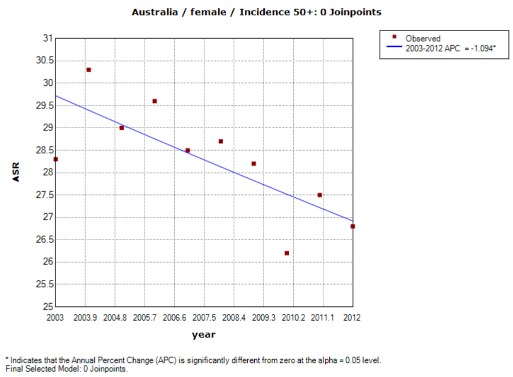

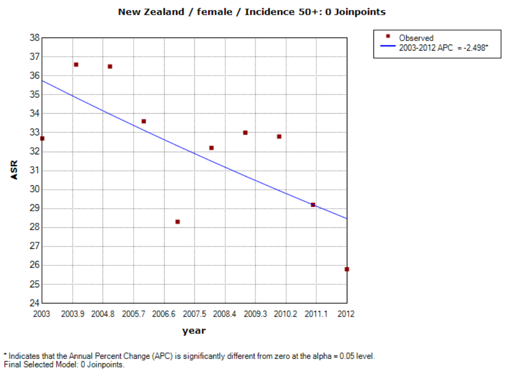

Northern America

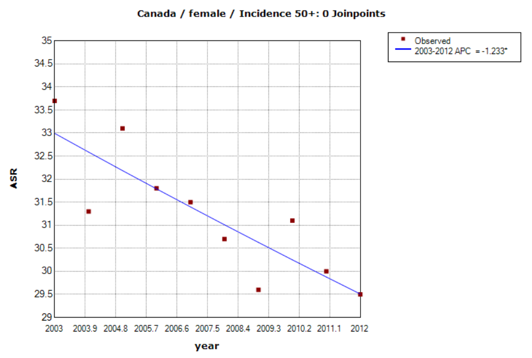

* Indicates that the Annual Percent Change (APC) is significantly different from zero at the alpha = 0.05 level.
Final Selected Model: 0 Joinpoints.

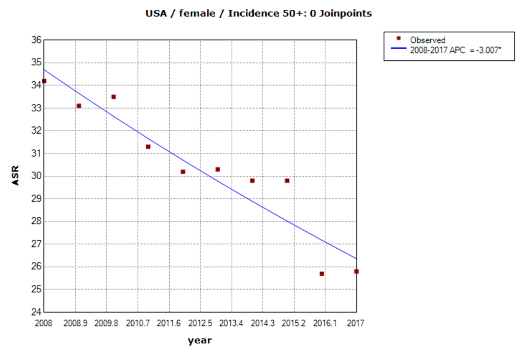

* Indicates that the Annual Percent Change (APC) is significantly different from zero at the alpha = 0.05 level.
Final Selected Model: 0 Joinpoints.

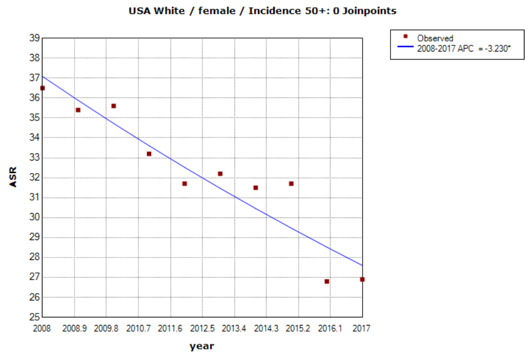

* Indicates that the Annual Percent Change (APC) is significantly different from zero at the alpha = 0.05 level.
Final Selected Model: 0 Joinpoints.

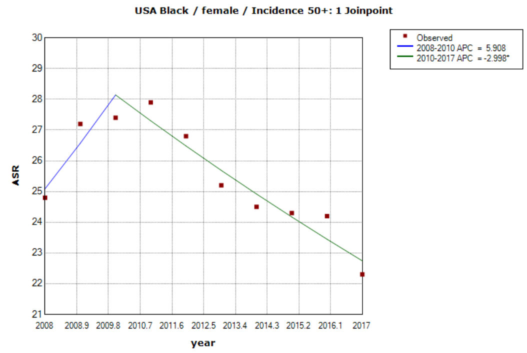

* Indicates that the Annual Percent Change (APC) is significantly different from zero at the alpha = 0.05 level.
Final Selected Model: 1 Joinpoint.

Southern America

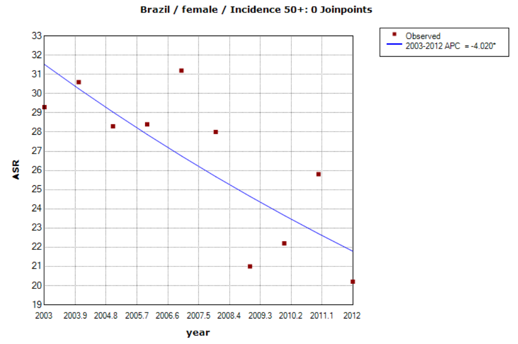

* Indicates that the Annual Percent Change (APC) is significantly different from zero at the alpha = 0.05 level.
Final Selected Model: 0 Joinpoints.

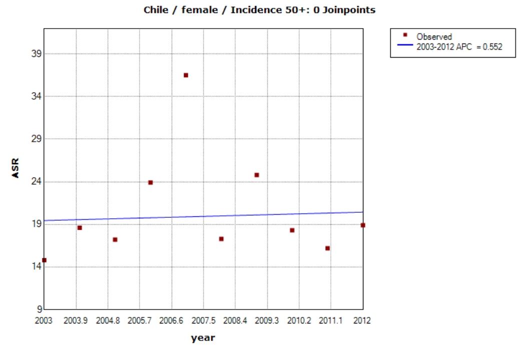

* Indicates that the Annual Percent Change (APC) is significantly different from zero at the alpha = 0.05 level.
Final Selected Model: 0 Joinpoints.

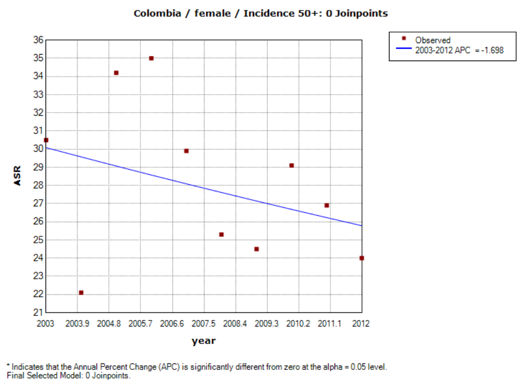

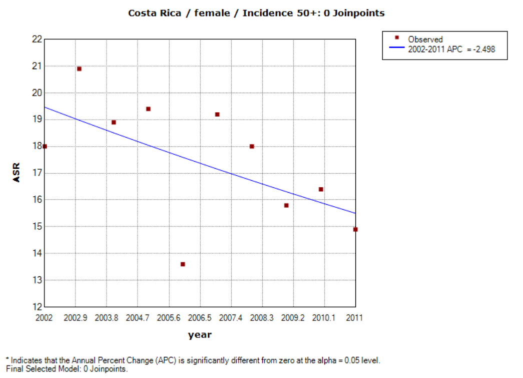

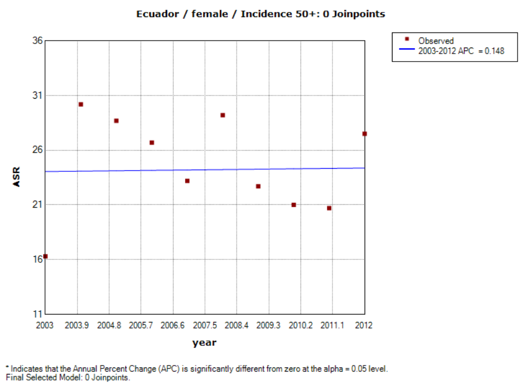

Northern Europe

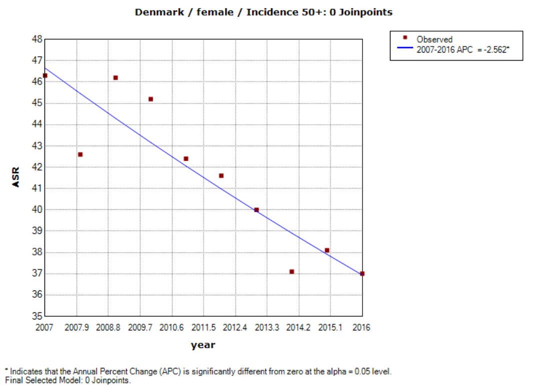

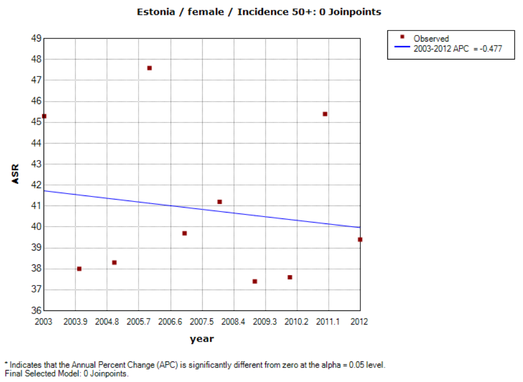

Faroe Islands / female / Incidence 50+: 0 Joinpoints

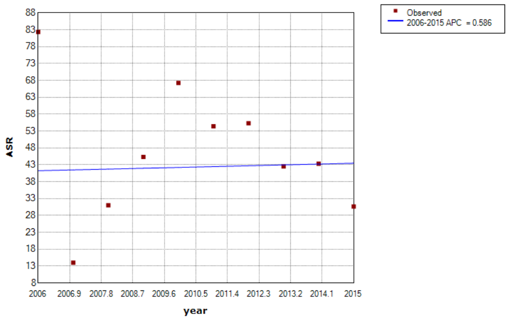

* Indicates that the Annual Percent Change (APC) is significantly different from zero at the alpha = 0.05 level.
Final Selected Model: 0 Joinpoints.

Finland / female / Incidence 50+: 1 Joinpoint

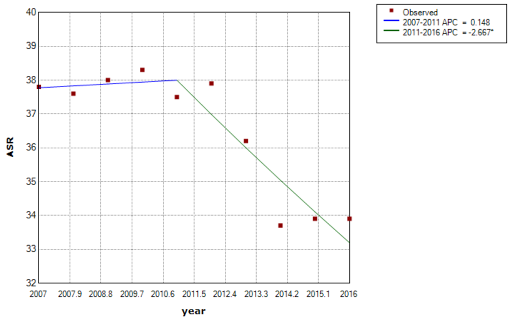

* Indicates that the Annual Percent Change (APC) is significantly different from zero at the alpha = 0.05 level.
Final Selected Model: 1 Joinpoint.

Iceland / female / Incidence 50+: 0 Joinpoints

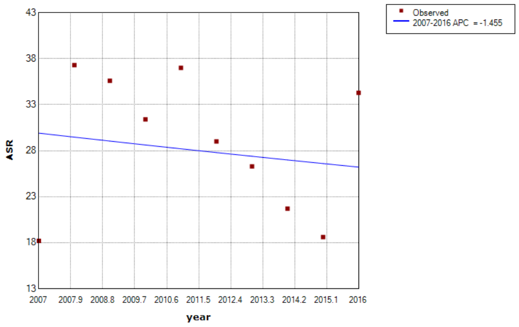

* Indicates that the Annual Percent Change (APC) is significantly different from zero at the alpha = 0.05 level.
Final Selected Model: 0 Joinpoints.

Ireland / female / Incidence 50+: 0 Joinpoints

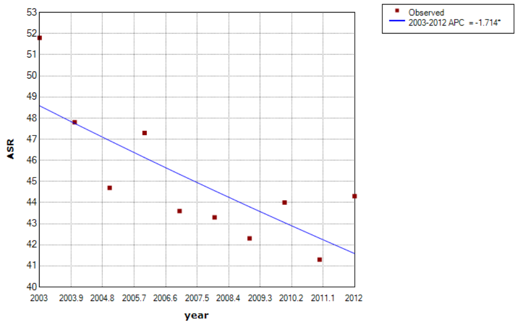

* Indicates that the Annual Percent Change (APC) is significantly different from zero at the alpha = 0.05 level.
Final Selected Model: 0 Joinpoints.

Lithuania / female / Incidence 50+: 0 Joinpoints

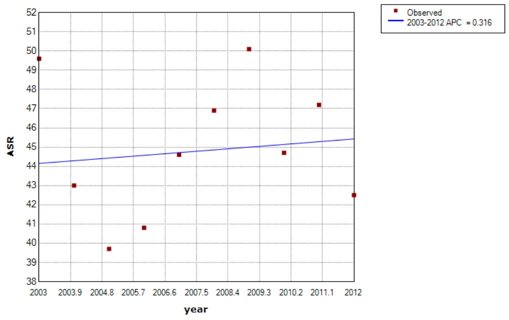

* Indicates that the Annual Percent Change (APC) is significantly different from zero at the alpha = 0.05 level.
Final Selected Model: 0 Joinpoints.

Norway / female / Incidence 50+: 0 Joinpoints

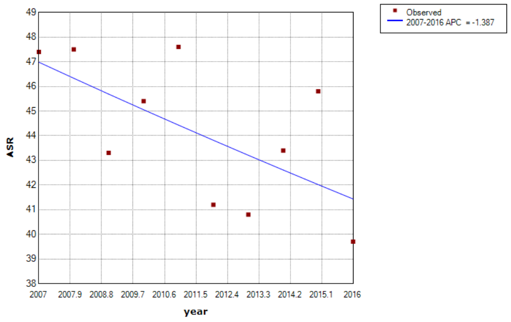

* Indicates that the Annual Percent Change (APC) is significantly different from zero at the alpha = 0.05 level.
Final Selected Model: 0 Joinpoints.

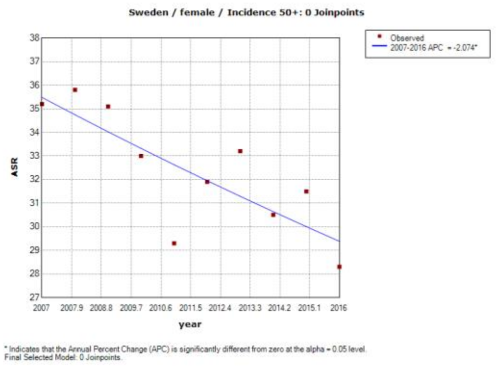

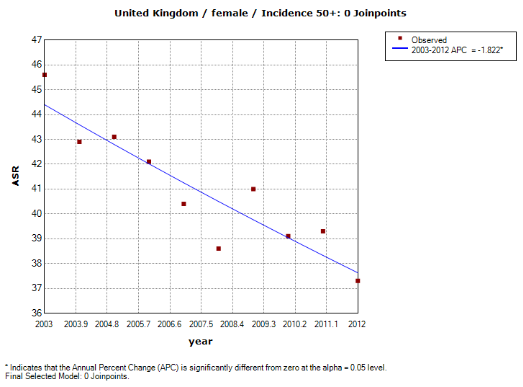

Western Europe

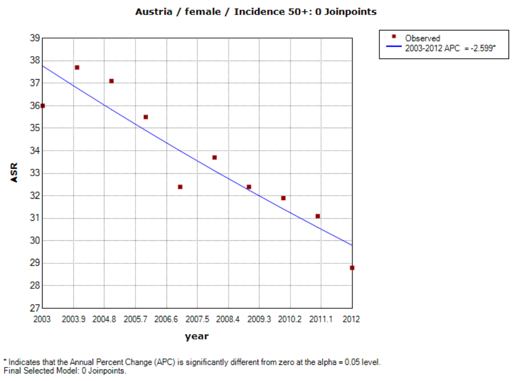

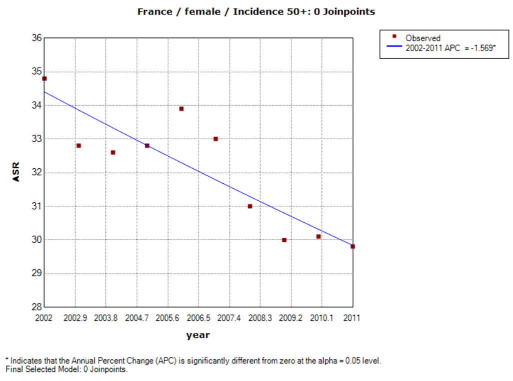

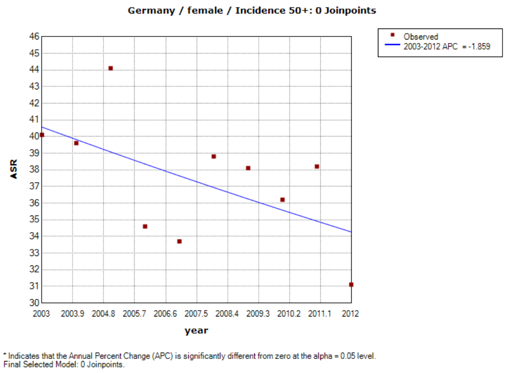

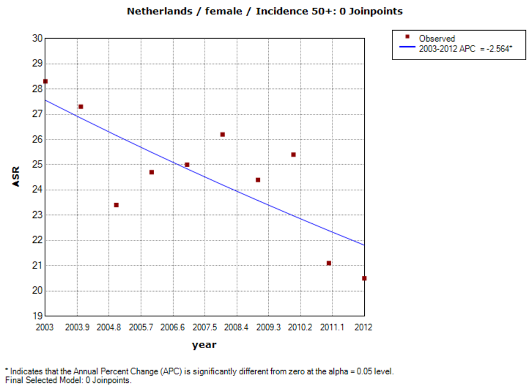

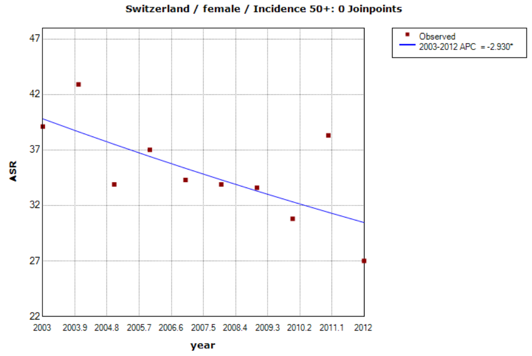

* Indicates that the Annual Percent Change (APC) is significantly different from zero at the alpha = 0.05 level.
Final Selected Model: 0 Joinpoints.

Southern Europe

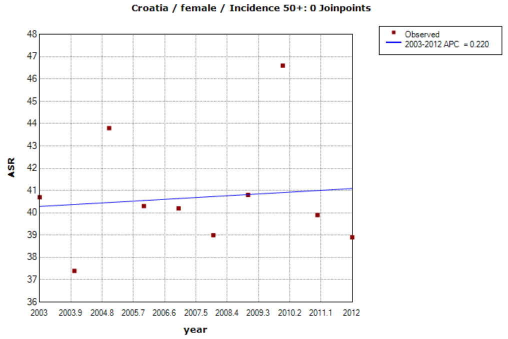

* Indicates that the Annual Percent Change (APC) is significantly different from zero at the alpha = 0.05 level.
Final Selected Model: 0 Joinpoints.

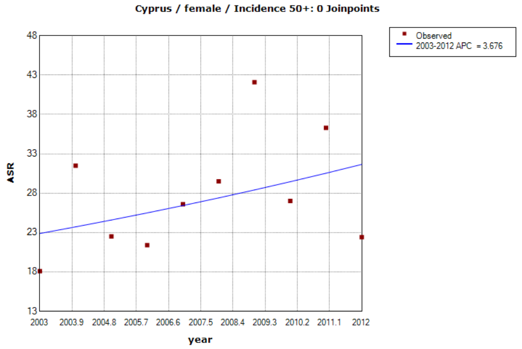

* Indicates that the Annual Percent Change (APC) is significantly different from zero at the alpha = 0.05 level.
Final Selected Model: 0 Joinpoints.

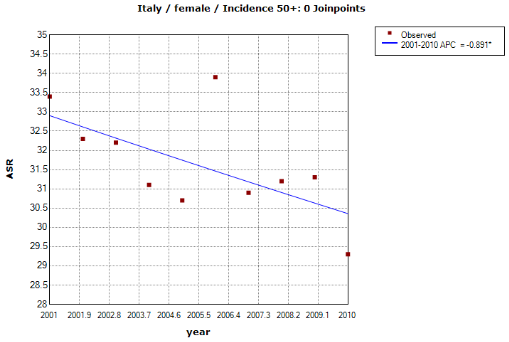

* Indicates that the Annual Percent Change (APC) is significantly different from zero at the alpha = 0.05 level.
Final Selected Model: 0 Joinpoints.

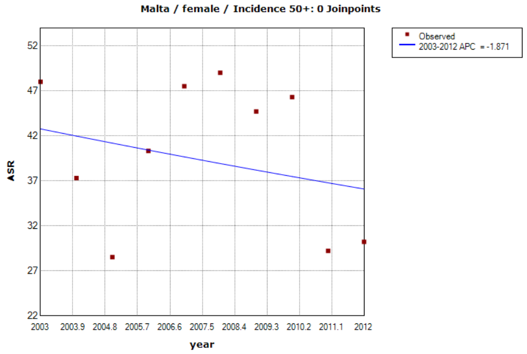

* Indicates that the Annual Percent Change (APC) is significantly different from zero at the alpha = 0.05 level.
Final Selected Model: 0 Joinpoints.

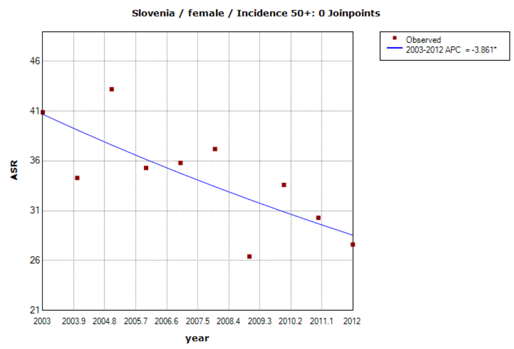

* Indicates that the Annual Percent Change (APC) is significantly different from zero at the alpha = 0.05 level.
Final Selected Model: 0 Joinpoints.

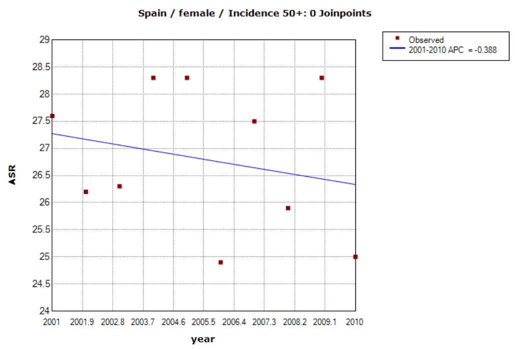

* Indicates that the Annual Percent Change (APC) is significantly different from zero at the alpha = 0.05 level.
Final Selected Model: 0 Joinpoints.

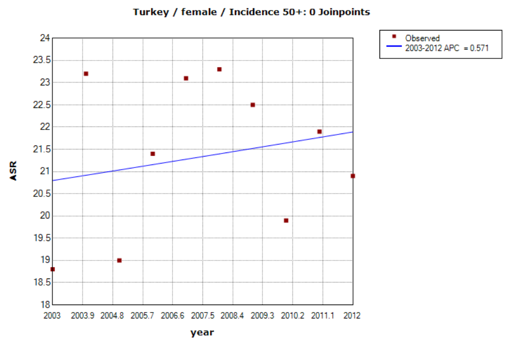

* Indicates that the Annual Percent Change (APC) is significantly different from zero at the alpha = 0.05 level.
Final Selected Model: 0 Joinpoints.

Eastern Europe

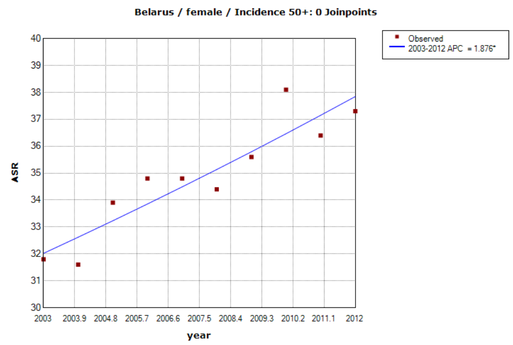

* Indicates that the Annual Percent Change (APC) is significantly different from zero at the alpha = 0.05 level.
Final Selected Model: 0 Joinpoints.

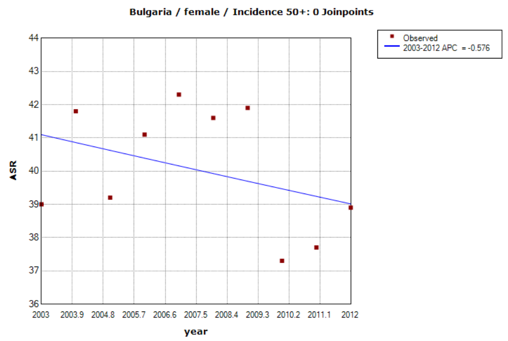

* Indicates that the Annual Percent Change (APC) is significantly different from zero at the alpha = 0.05 level.
Final Selected Model: 0 Joinpoints.

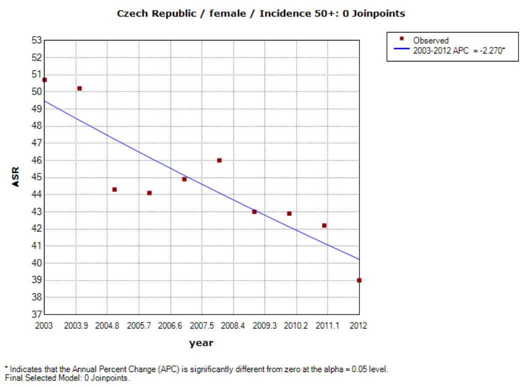
Czech Republic / female / Incidence 50+: 0 Joinpoints
Observed
2003-2012 APC = -2.270*
ASR
year
* Indicates that the Annual Percent Change (APC) is significantly different from zero at the alpha = 0.05 level.
Final Selected Model: 0 Joinpoints.

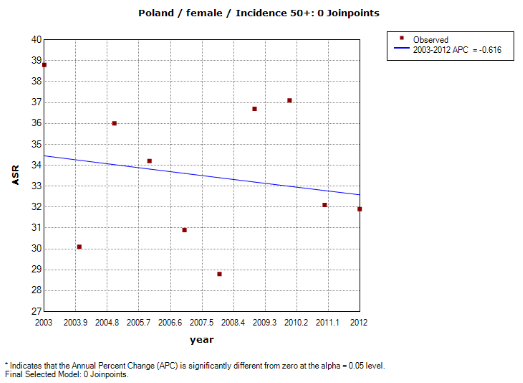
Poland / female / Incidence 50+: 0 Joinpoints
Observed
2003-2012 APC = -0.616
ASR
year
* Indicates that the Annual Percent Change (APC) is significantly different from zero at the alpha = 0.05 level.
Final Selected Model: 0 Joinpoints.

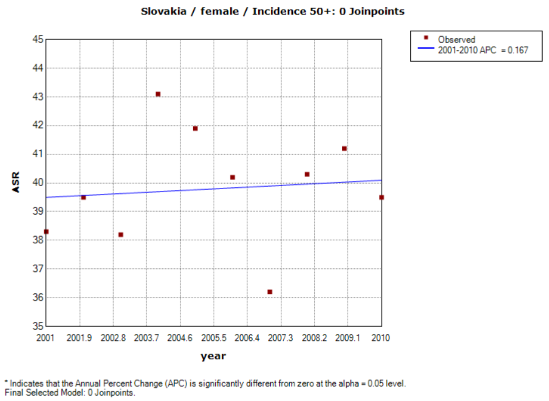
Slovakia / female / Incidence 50+: 0 Joinpoints
Observed
2001-2010 APC = 0.167
ASR
year
* Indicates that the Annual Percent Change (APC) is significantly different from zero at the alpha = 0.05 level.
Final Selected Model: 0 Joinpoints.

Africa

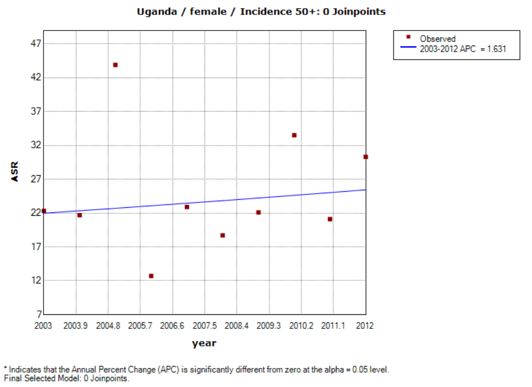
Uganda / female / Incidence 50+: 0 Joinpoints
Observed
2003-2012 APC = 1.631
ASR
year
* Indicates that the Annual Percent Change (APC) is significantly different from zero at the alpha = 0.05 level.
Final Selected Model: 0 Joinpoints.

e.) Mortality female all ages

Asia

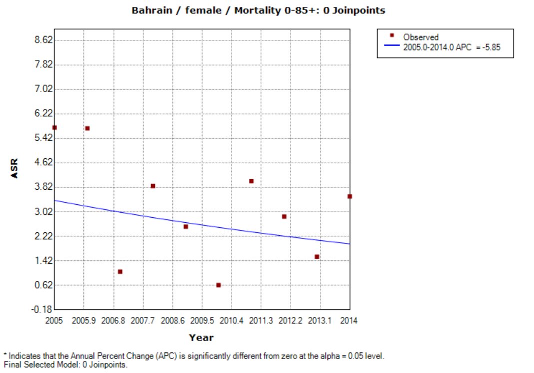

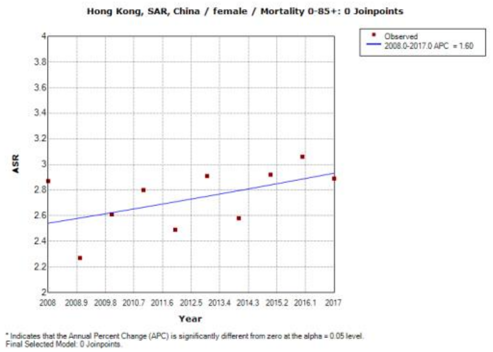

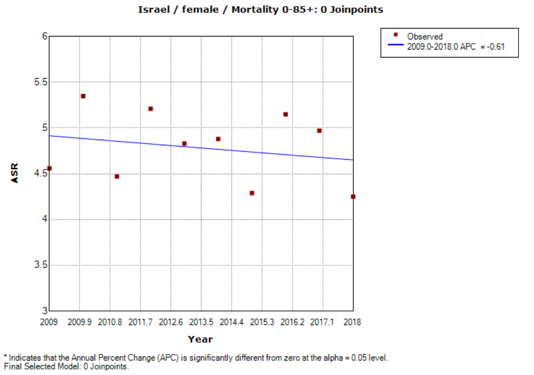

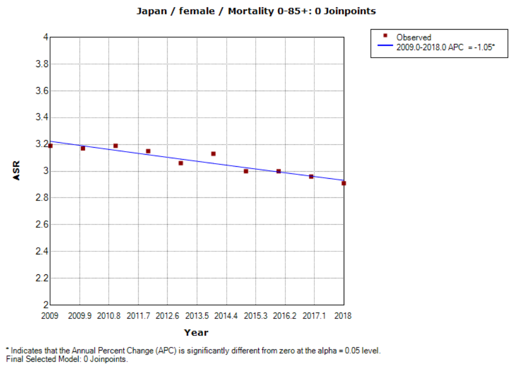

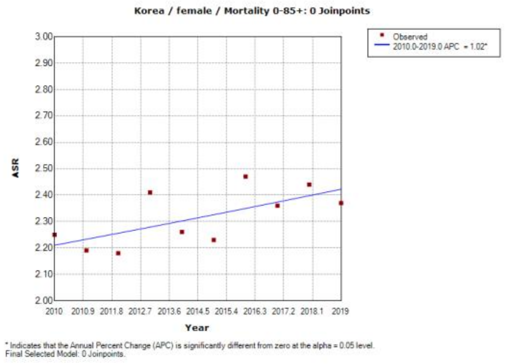

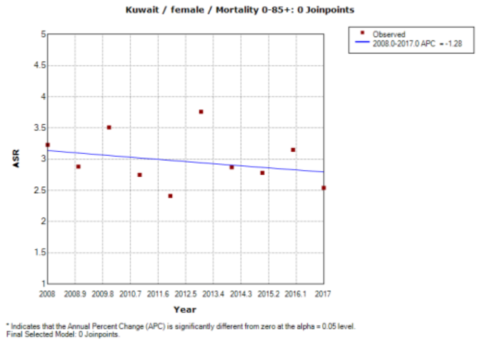

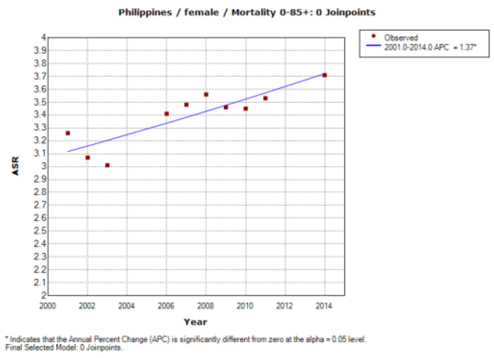

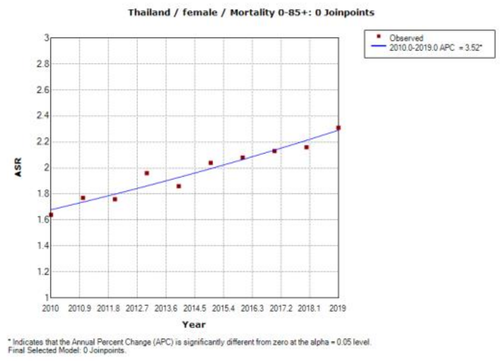

Oceania

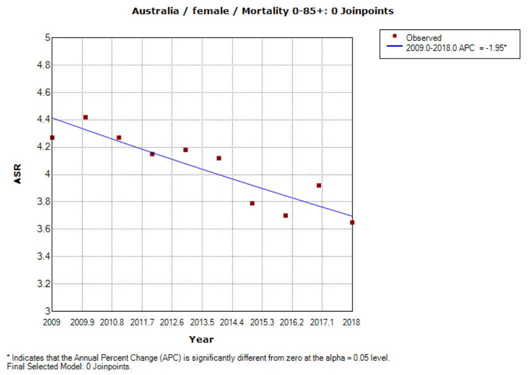

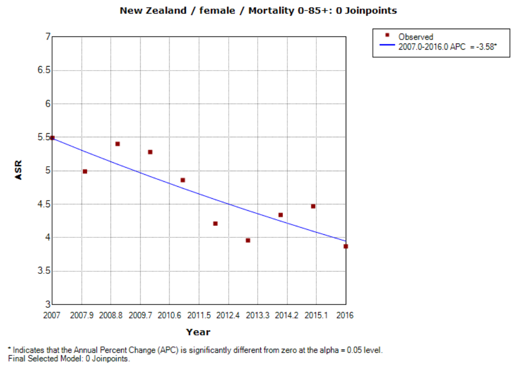

Northern America

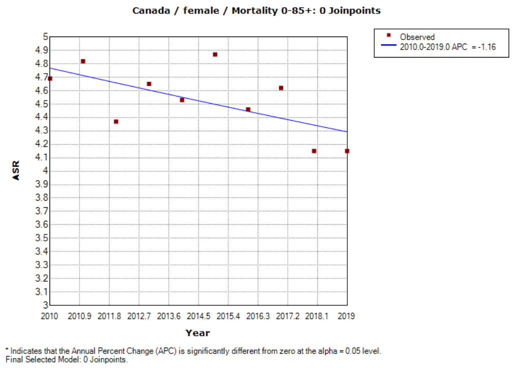

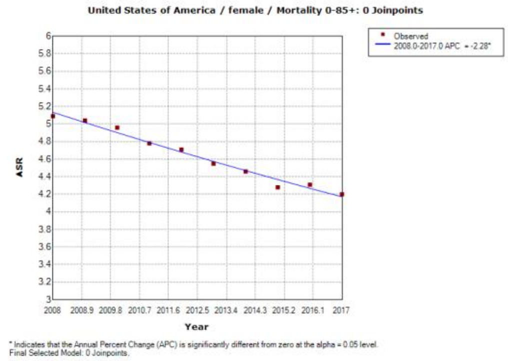

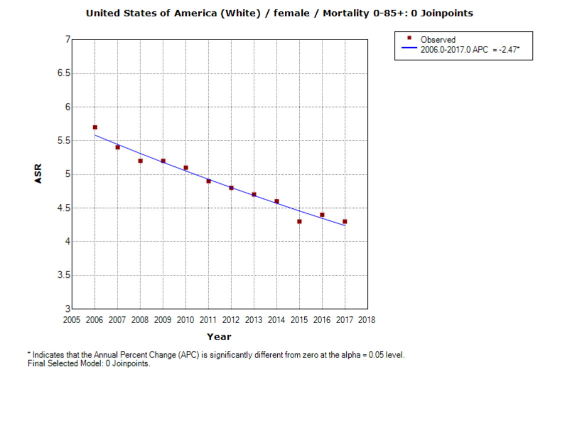

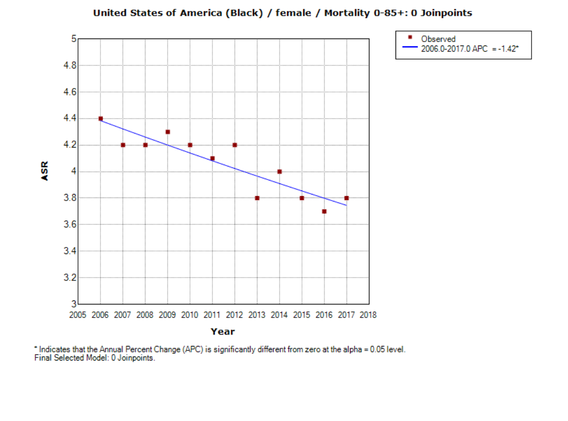

Southern America

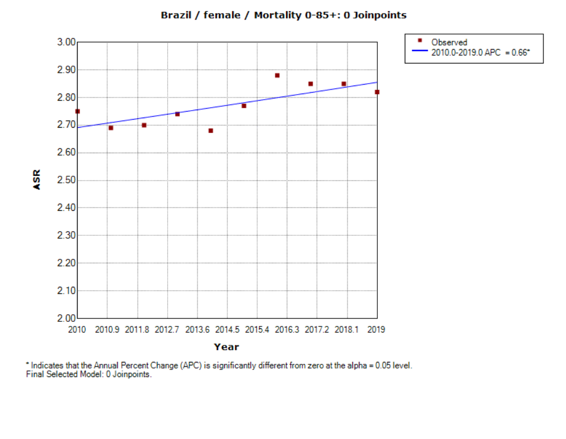

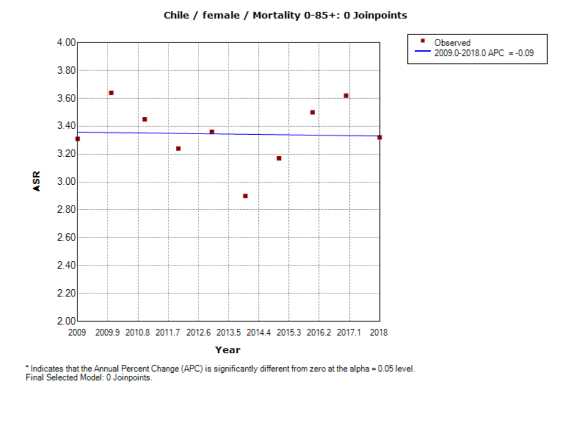

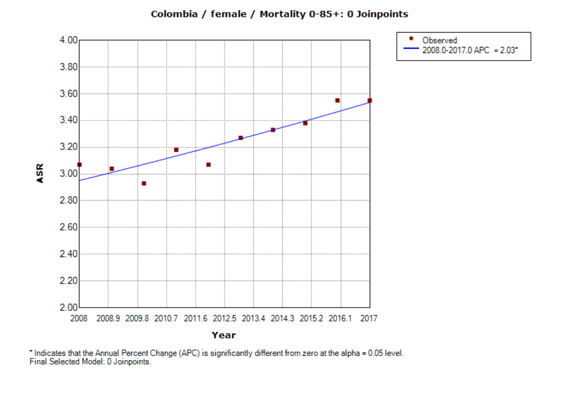

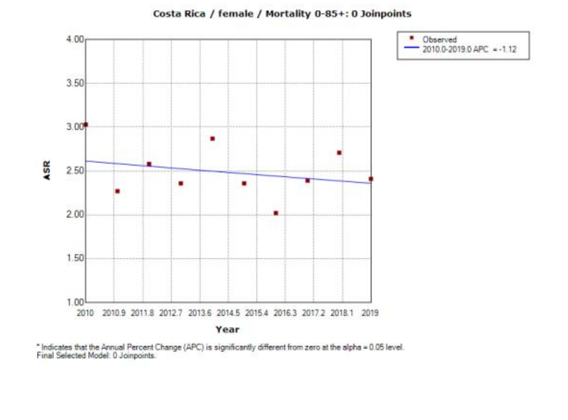

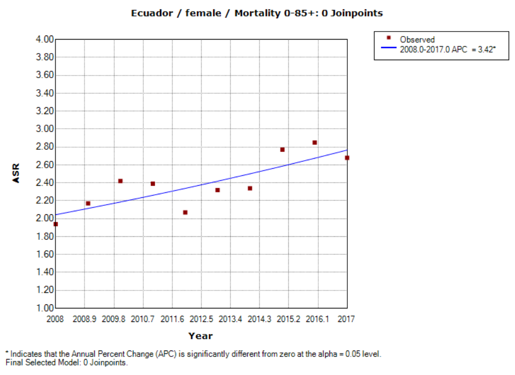

Northern Europe

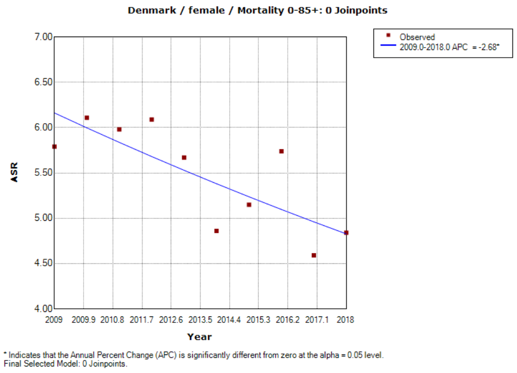

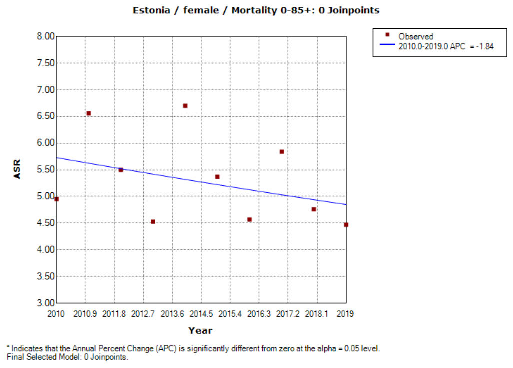

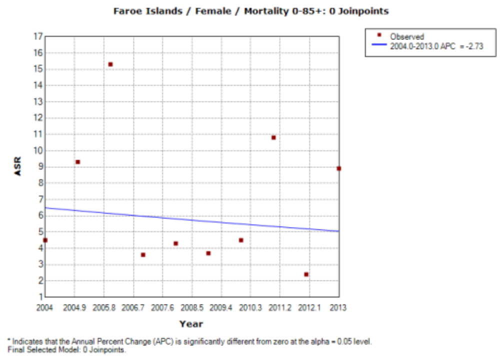

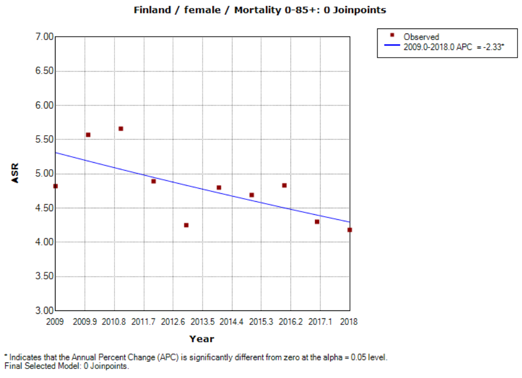

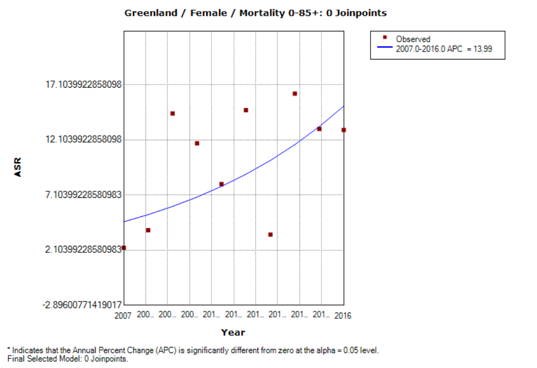
Greenland / Female / Mortality 0-85+: 0 Joinpoints
Observed
2007.0-2016.0 APC = 13.99
ASR
17.1039922858098
12.1039922858098
7.10399228580983
2.10399228580983
-2.89600771419017
2007
2016
Year
* Indicates that the Annual Percent Change (APC) is significantly different from zero at the alpha = 0.05 level.
Final Selected Model: 0 Joinpoints.

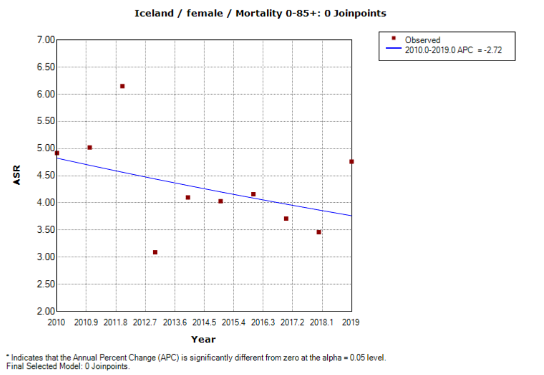
Iceland / female / Mortality 0-85+: 0 Joinpoints
Observed
2010.0-2019.0 APC = -2.72
ASR
7.00
6.50
6.00
5.50
5.00
4.50
4.00
3.50
3.00
2.50
2.00
2010 2010.9 2011.8 2012.7 2013.6 2014.5 2015.4 2016.3 2017.2 2018.1 2019
Year
* Indicates that the Annual Percent Change (APC) is significantly different from zero at the alpha = 0.05 level.
Final Selected Model: 0 Joinpoints.

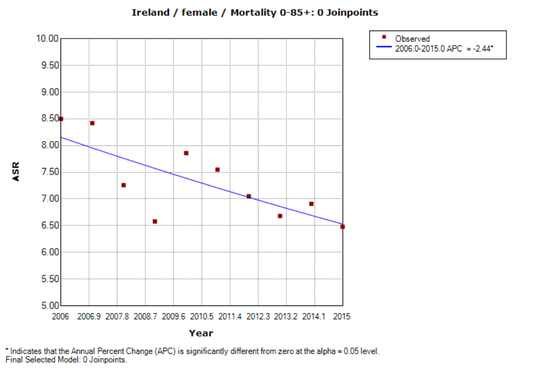
Ireland / female / Mortality 0-85+: 0 Joinpoints
Observed
2006.0-2015.0 APC = -2.44*
ASR
10.00
9.50
9.00
8.50
8.00
7.50
7.00
6.50
6.00
5.50
5.00
2006 2006.9 2007.8 2008.7 2009.6 2010.5 2011.4 2012.3 2013.2 2014.1 2015
Year
* Indicates that the Annual Percent Change (APC) is significantly different from zero at the alpha = 0.05 level.
Final Selected Model: 0 Joinpoints.

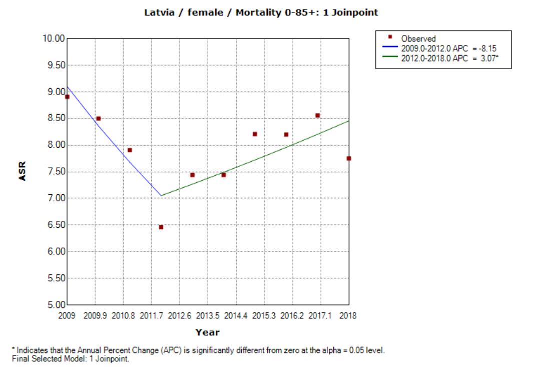
Latvia / female / Mortality 0-85+: 1 Joinpoint
Observed
2009.0-2012.0 APC = -8.15
2012.0-2018.0 APC = 3.07*
ASR
10.00
9.50
9.00
8.50
8.00
7.50
7.00
6.50
6.00
5.50
5.00
2009 2009.9 2010.8 2011.7 2012.6 2013.5 2014.4 2015.3 2016.2 2017.1 2018
Year
* Indicates that the Annual Percent Change (APC) is significantly different from zero at the alpha = 0.05 level.
Final Selected Model: 1 Joinpoint.

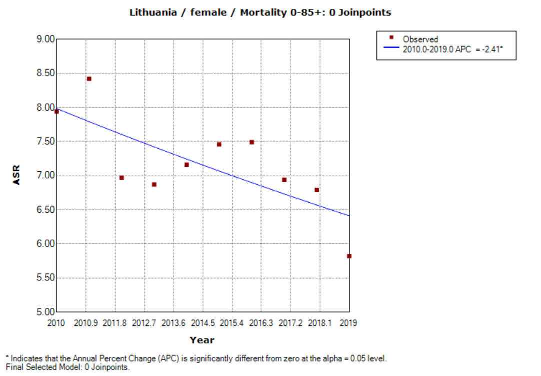
Lithuania / female / Mortality 0-85+: 0 Joinpoints
Observed
2010.0-2019.0 APC = -2.41*
ASR
9.00
8.50
8.00
7.50
7.00
6.50
6.00
5.50
5.00
2010 2010.9 2011.8 2012.7 2013.6 2014.5 2015.4 2016.3 2017.2 2018.1 2019
Year
* Indicates that the Annual Percent Change (APC) is significantly different from zero at the alpha = 0.05 level.
Final Selected Model: 0 Joinpoints.

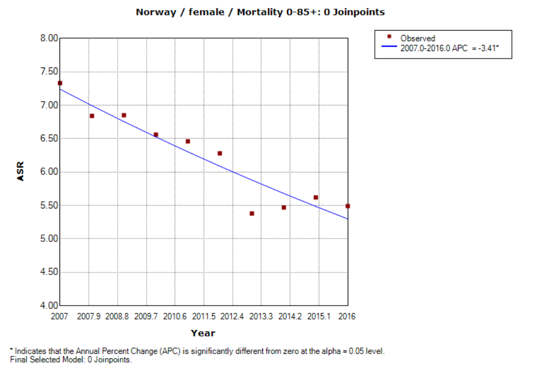
Norway / female / Mortality 0-85+: 0 Joinpoints
Observed
2007.0-2016.0 APC = -3.41*
ASR
8.00
7.50
7.00
6.50
6.00
5.50
5.00
4.50
4.00
2007 2007.9 2008.8 2009.7 2010.6 2011.5 2012.4 2013.3 2014.2 2015.1 2016
Year
* Indicates that the Annual Percent Change (APC) is significantly different from zero at the alpha = 0.05 level.
Final Selected Model: 0 Joinpoints.

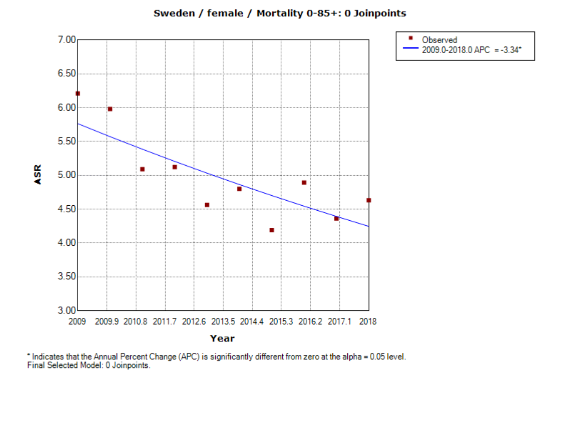

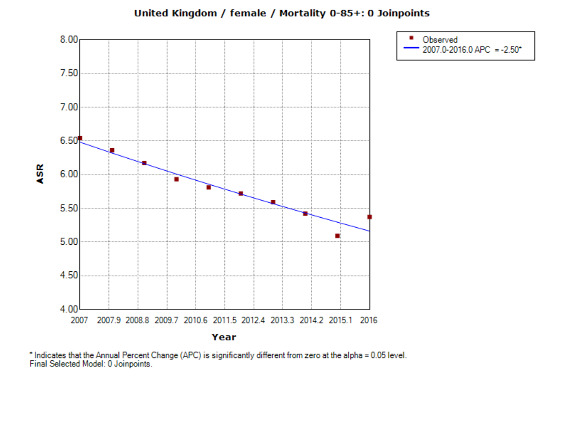

Western Europe

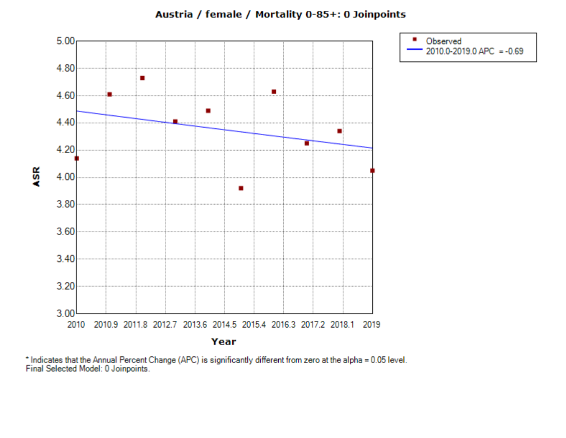

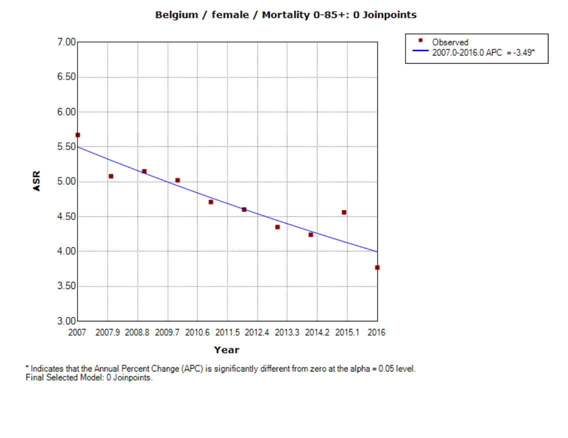

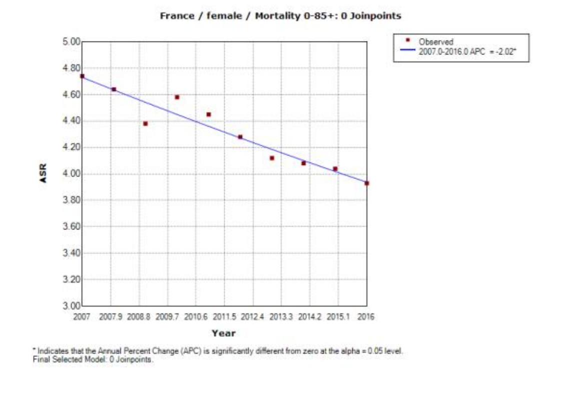

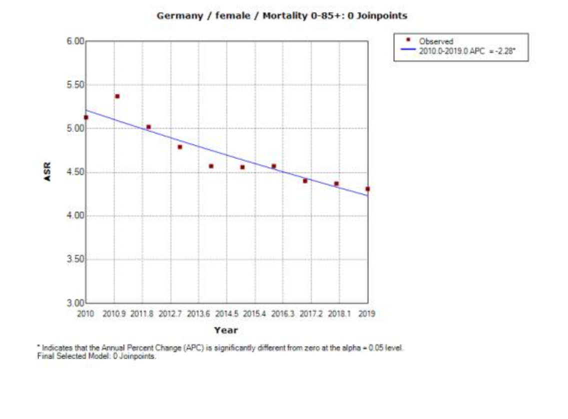

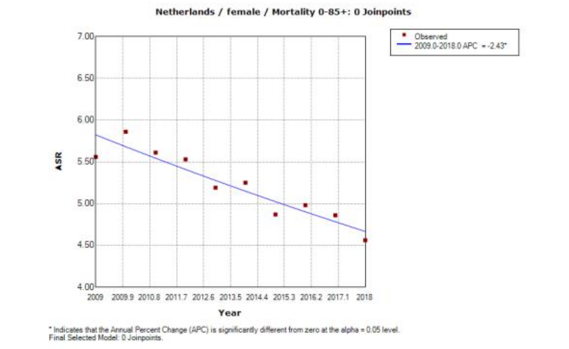

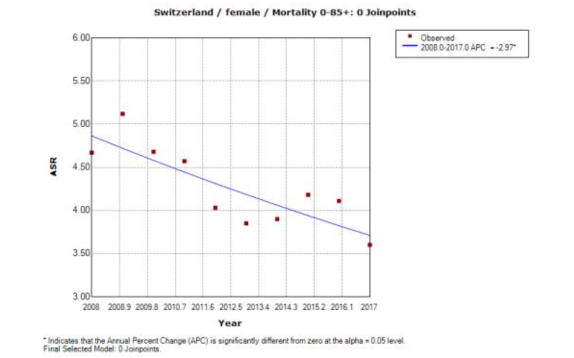

Southern Europe

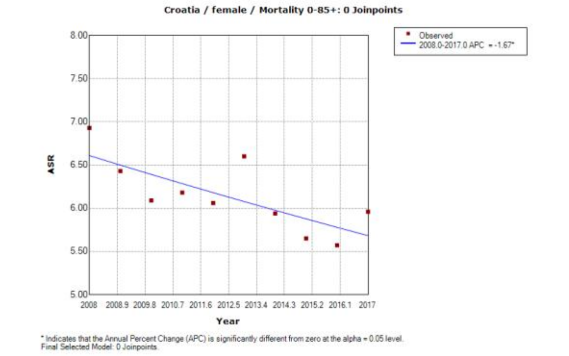

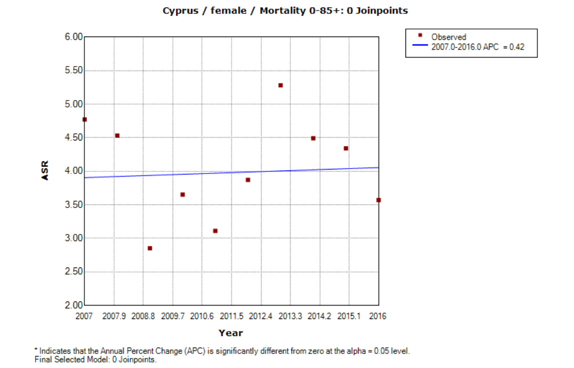

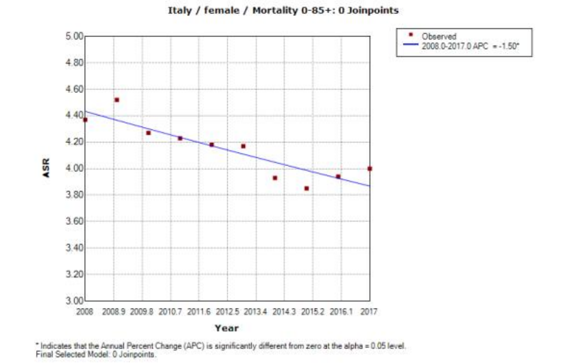

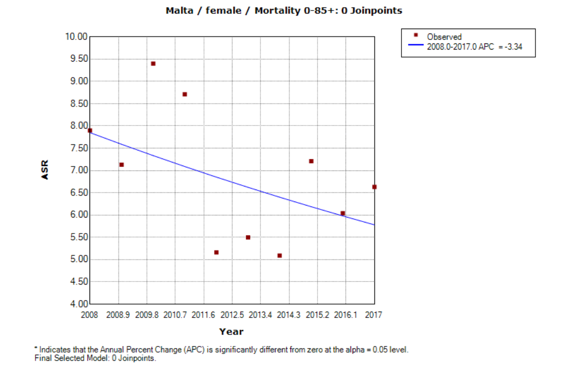

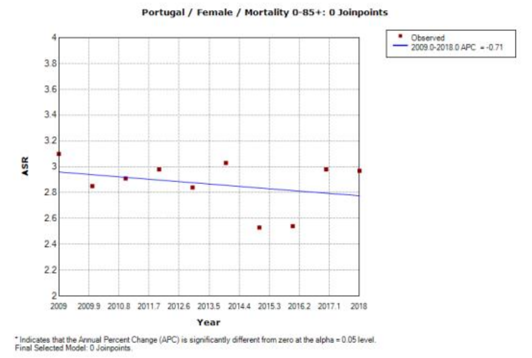

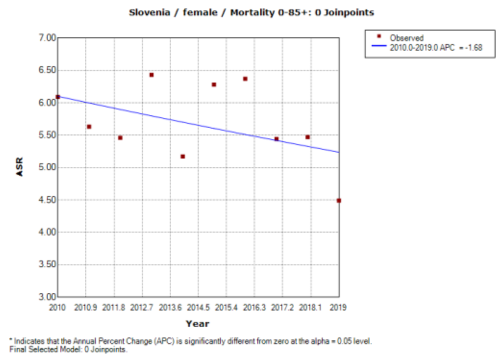

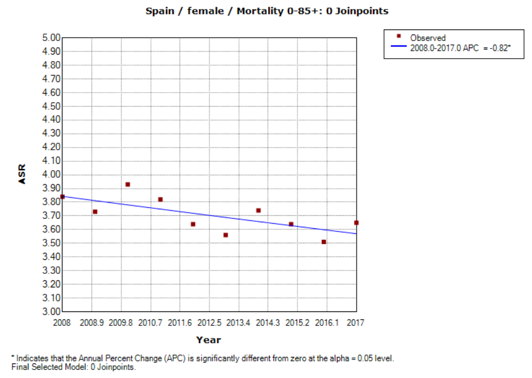

Eastern Europe

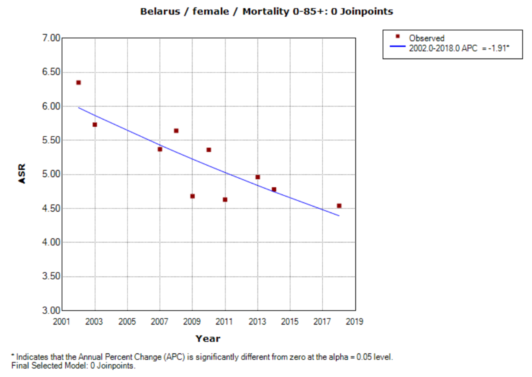

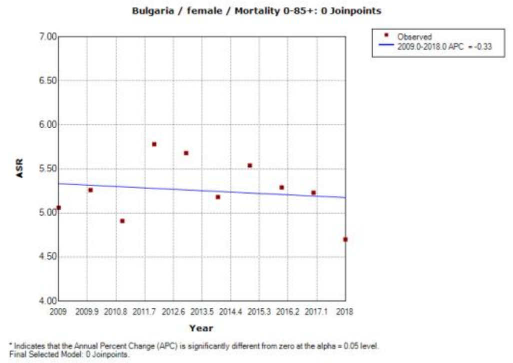

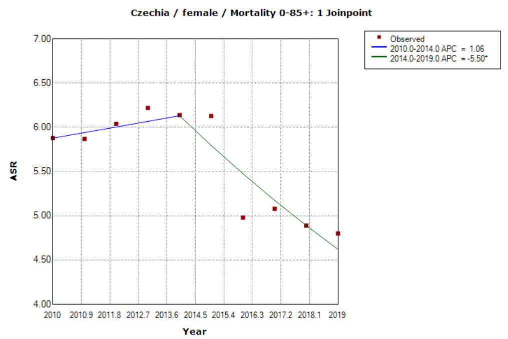

* Indicates that the Annual Percent Change (APC) is significantly different from zero at the alpha = 0.05 level.
Final Selected Model: 1 Joinpoint.

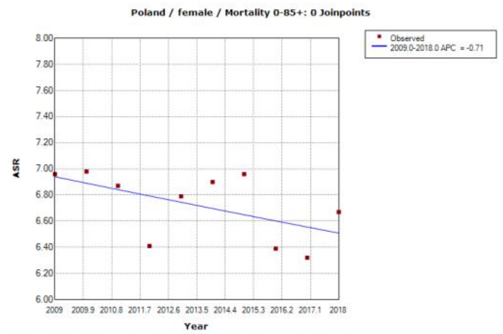

* Indicates that the Annual Percent Change (APC) is significantly different from zero at the alpha = 0.05 level.
Final Selected Model: 0 Joinpoints.

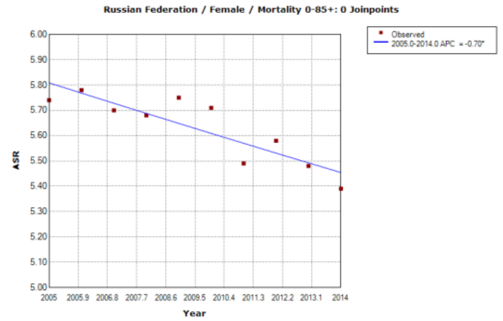

* Indicates that the Annual Percent Change (APC) is significantly different from zero at the alpha = 0.05 level.
Final Selected Model: 0 Joinpoints.

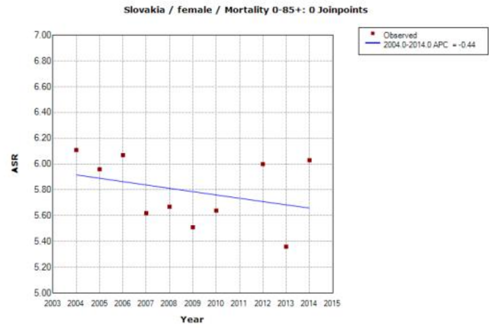

* Indicates that the Annual Percent Change (APC) is significantly different from zero at the alpha = 0.05 level.
Final Selected Model: 0 Joinpoints.

Supplementary Figure S3. AAPC of incidence of ovarian cancer aged 50 years and older

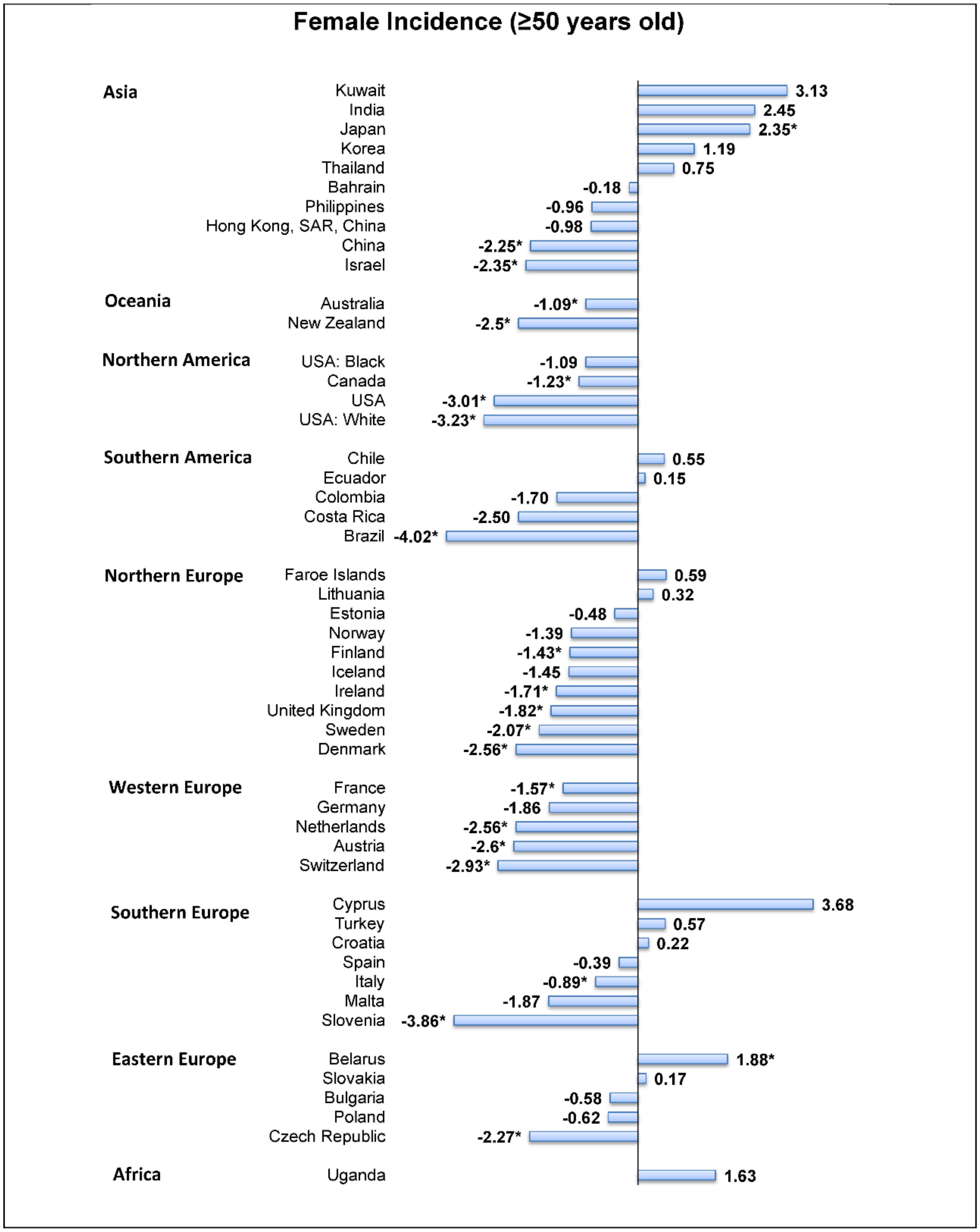

Supplementary Figure S4. AAPC of incidence of ovarian cancer aged < 50 years old

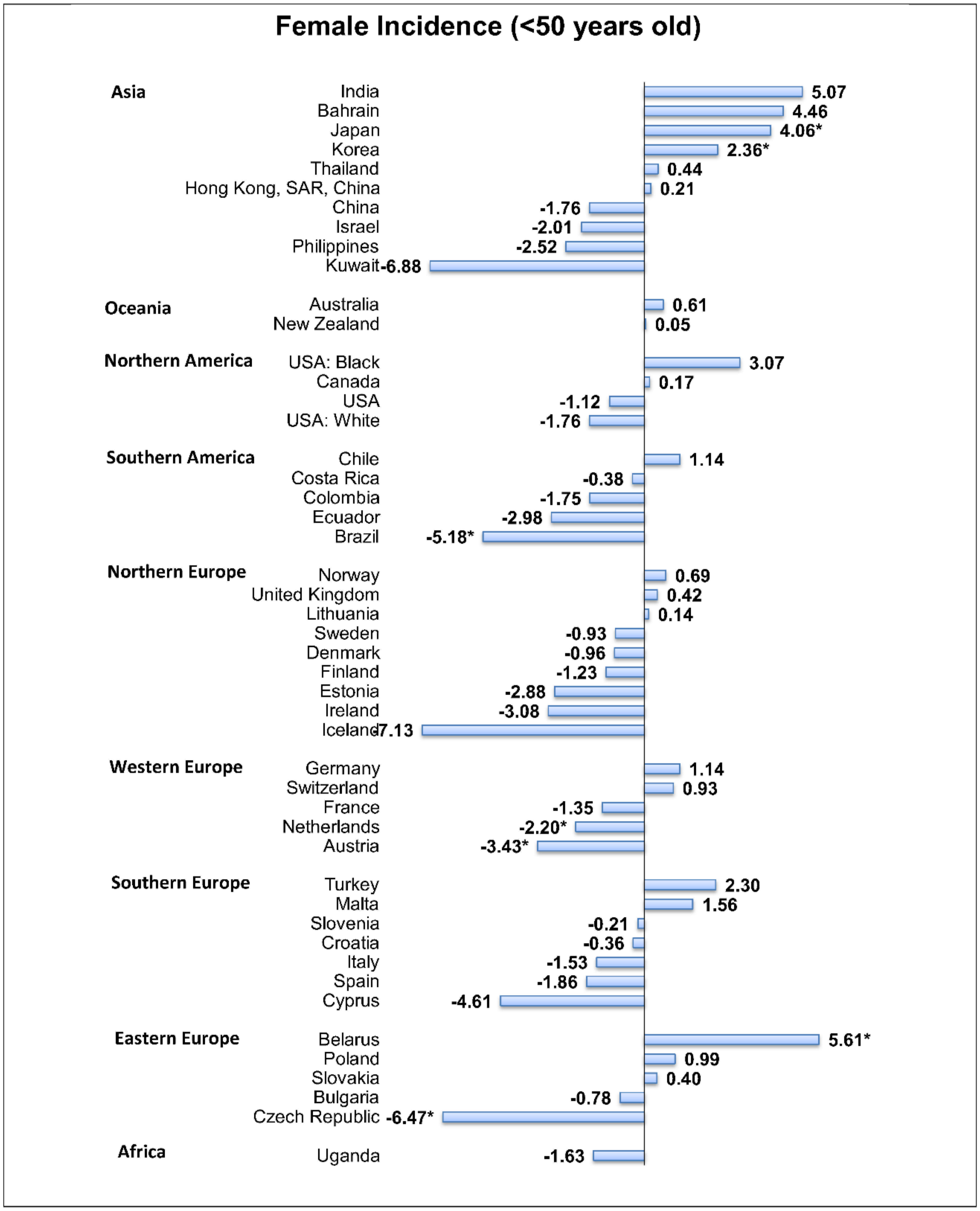

Supplementary Figure S5. AAPC of incidence of ovarian cancer aged < 40 years old

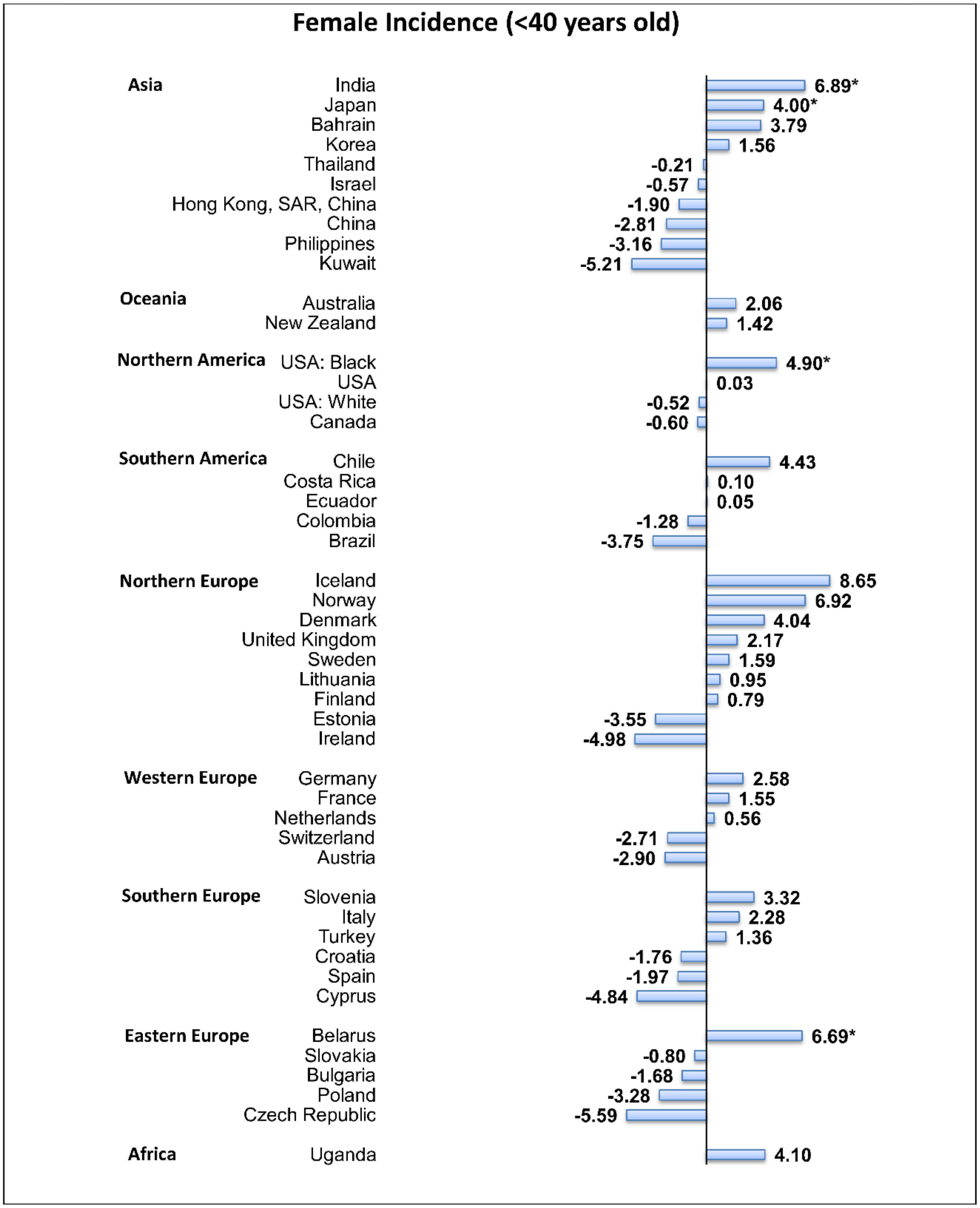

Supplement: Supplementary file 1 [file cancers-14-02230-s001.zip › cancers-1686744-supplementary.pdf]
